# Supramolecular assembly of hypervalent iodine macrocycles and alkali metals

**DOI:** 10.3762/bjoc.21.87

**Published:** 2025-05-30

**Authors:** Krishna Pandey, Lucas X Orton, Grayson Venus, Waseem A Hussain, Toby Woods, Lichang Wang, Kyle N Plunkett

**Affiliations:** 1 School of Chemical and Biomolecular Sciences, Southern Illinois University, Carbondale, IL 62901, United States; 2 George L. Clark X-ray Facility and 3M Materials Laboratory, University of Illinois at Urbana-Champaign, Urbana, IL, 61801, United States

**Keywords:** hypervalent iodine, macrocycle, metal coordination, supramolecular

## Abstract

This study explores the solution- and solid-state assembly of phenylalanine-based hypervalent iodine macrocycles (HIMs) with lithium and sodium cations. The metal cation binding of HIMs was evaluated by addition of lithium tetrakis(pentafluorophenyl)borate ethyl etherate LiBArF_20_ and sodium tetrakis[3,5-bis(trifluoromethyl)phenyl]borate NaBArF_24_. The electron-rich, outwardly projected carbonyl oxygens of the HIM co-crystalize with the cations into bent supramolecular architectures. Both crystal structures show a pattern of assembly between HIM and metal cation in 2:1 ratio. While association with sodium leads to a polymer-like network, the lithium crystal structure was limited to dimeric assemblies of HIM. In the lithium-coordinating complex, the oxygen–lithium–oxygen bond angle is approximately 98.83°, displaying a closer arrangement of two HIMs. In contrast, the sodium complex exhibits a more open orientation of two HIMs with an oxygen–sodium–oxygen bond angle close to 167.98°. Lastly, a comparative study of association constants and binding energies for phenylalanine-based HIM with LiBArF_20_ and NaBArF_24_ are presented.

## Introduction

**Figure 1:**
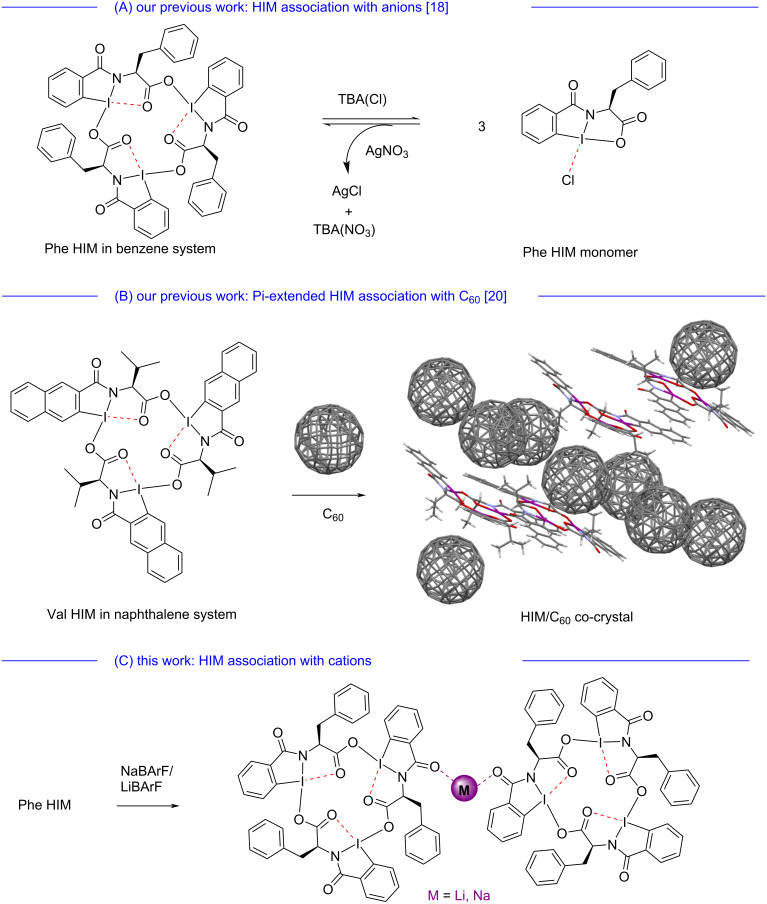
(A) Our previous work: Assembly and disassembly of phenylalanine hypervalent iodine macrocycles (Phe HIM) through anion coordination. (B) Another work on π-extended hypervalent iodine macrocycle and their supramolecular assembly with Buckminster fullerene. (C) This work: Association of Phe HIM with first group alkali metal cations. Secondary bonds are highlighted in red.

Supramolecular chemistry is emerging as a pivotal area of research in both medicinal and materials chemistry that opens the avenue for new functionalized materials for their use in medical devices or therapeutics [1], drug delivery [2], supramolecular sensing [3], purification [4], and separation [5–6]. The interactions in supramolecular assemblies are driven by well-known hydrogen-bonding, hydrophobic interactions, electrostatic interactions and π–π stacking [7–8]. The supramolecular chemistries of hypervalent iodine systems that involve secondary bonding to form higher order molecular assemblies are yet to be fully explored. In general, the atom’s capacity to extend its valence shell beyond the usual limitations of a Lewis octet is known as hypervalency [9]. The modern periodic table demonstrates several atoms (e.g., Cl, I, S, P etc.) that exhibit hypervalent properties. Among these atoms, iodine has gained significant attention, particularly in the form of hypervalent iodine reagents. In organic chemistry, these reagents are valued for their distinct reactivities, safe handling, high durability, low harmful, and convenient alternative to heavy-metal reagents [10–11]. Hypervalent iodine compounds exist as trivalent (λ^3^-iodanes) or pentavalent (λ^5^-iodanes) species. The λ^3^-iodanes have an T-shaped structure [12], with the energetically preferred arrangement of the two most electronegative heteroatoms at axial positions resulting in a three-center four-electron bond [13–14]. In this unique “T” configuration, the bond length between iodine and one of the heteroatoms is influenced by the bond distance of the other heteroatom and is responsible for the stabilization of the hypervalent iodine system [12]. In addition to these covalent bonding interactions, additional secondary bonding (e.g., red-dotted bonds between iodine and oxygen in phenylalanine-based hypervalent iodine macrocycle in benzene system in Figure 1A) can arise in these systems from neighboring atom lone pairs.

Secondary bonding is characterized as those interactions that involve intermolecular hypervalent connections with lengths shorter than the sum of the van der Waals radii between a heavy p-block element and an electron-pair donor (typically O, N, S, or halogen) [15]. Secondary I···O interactions have been found to help assemble higher order supramolecular hypervalent iodine macrocycles [16–18]. Examples of hypervalent iodine macrocycles (HIMs) include those synthesized by Ochiai [16], Tykwinski and Zhdankin [17], and our group’s extension of those works (Figure 1A and B) [18–19]. The main driving force for the assembly of HIM is attributed to the weak, yet additive, secondary bonding interactions between electron-deficient iodine atoms and electron-rich oxygen atoms. These examples highlight new possibilities in materials science owing to their unique assembly via secondary bonding as well as their dynamic assembly and disassembly.

Our previous study [18] demonstrates the synthesis of various HIM variants, including a new phenylalanine-based HIM, which show the dynamic behavior of HIM assemblies in solution via addition and removal of anions (Figure 1A). Moreover, due to secondary bonding in the HIM systems, these macrocycles display dynamic behavior even in the absence of extra anions, with monomers swapping between macrocycles to participate in dynamic covalent chemistry. As a demonstration of even higher supramolecular assemblies, we also showed π-extended HIMs enable the co-assembly of Buckminster fullerene into long range assembled structures (Figure 1B) [20].

In this contribution, we explored the cation binding abilities of HIMs with first group alkali metals such as lithium and sodium. The phenylalanine-based HIM **1** used in this study was re-synthesized following the previously reported protocol, and the characterization data are consistent with the original dataset [18]. Through a series of crystallographic experiments, we demonstrate that HIMs coordinate with alkali metals through the periphery carbonyl oxygens via metal–oxygen bonding to form a higher order metal-coordinated hypervalent iodine-based macrocyclic complex. Furthermore, we have experimentally and computationally compared the association constants of lithium tetrakis(pentafluorophenyl)borate ethyl etherate LiBArF_20_ and sodium tetrakis[3,5-bis(trifluoromethyl)phenyl]borate NaBArF_24_ in the phenylalanine HIM system.

## Results and Discussion

We have reported the synthesis of several variations of valine and phenylalanine-based HIMs that are based on benzene-, naphthalene-, and anthraquinone systems [18–19]. This study employed a phenylalanine and iodobenzoic acid-based HIM system owing to its simplicity in synthesis and ease of crystal growth. Following our previously established synthetic route [18], phenylalanine-based HIM **1** was synthesized in three steps by utilizing commercially available 2-iodobenzoic acid and ʟ-Phe-O*t*-Bu as starting materials (Supporting Information File 1).

**Figure 2:**
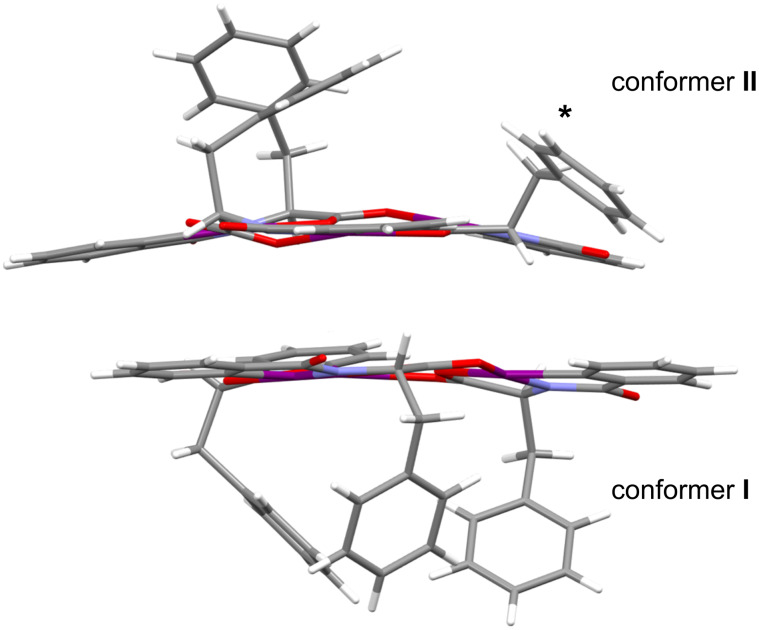
Two conformations of the HIM were found. One conformation projected all three benzyl groups in a vertical arrangement **I** (bottom) while the second conformation **II** projected only two vertically. One benzyl ring is pointed outside as highlighted by * (top). Oxygen, nitrogen, and iodine atoms are denoted by red, light blue, and purple color, respectively.

Phenylalanine HIM **1** was previously isolated in crystalline form in two different molecular conformations [18]. In conformer **I** (Figure 2, bottom), all three benzyl groups are oriented towards the interior of the macrocycle. In contrast, conformer **II** (Figure 2, top) features two benzyl groups projecting inward and one benzyl group projecting outward denoted by an asterisk. Furthermore, the resulting crystal structure reveals that **1** is also a distorted planar macrocyclic system consisting of the amino acids carbonyl oxygens facing inside the ring. All three benzyl groups are located above a single plane (more figures are provided in Supporting Information File 1, Figure S1).

**Figure 3:**
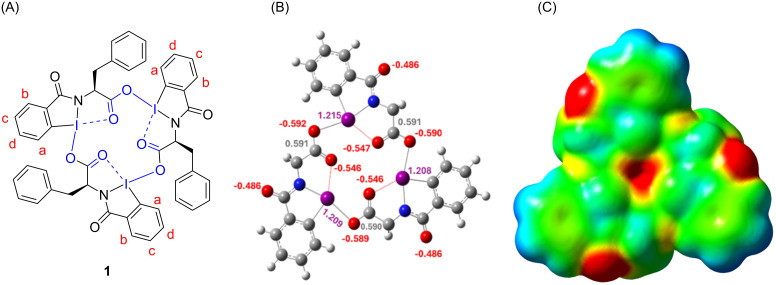
A) Chemical structure of HIM **1**: Three iodine atoms and three inward projected ester carbonyls curcumscribe the small cyclic core represented by blue color. Three carbonyl amides project outward. B) DFT image of **1** displaying the distribution of Mulliken charges on iodine (purple), oxygen (red), and carbon (grey); benzyl groups are ommited for clarity. C) Calculated electrostatic potential map showing the electrophilic core highlighted by a smaller area of red color surrounded by green color of **1**. Electron-rich periphery oxygens (formerly the carbonyl of benzoic acid) are highlighted by a greater area of red color.

In addition, the crystal structure also demonstrates that the three iodine atoms, three carbonyl carbon atoms and six oxygen atoms circumscribe a small bowl-like cavity within the macrocycle. With the consideration of all primary and secondary bonds, the small cavity is highlighted by blue color in the chemical structure of phenylalanine HIM as shown in Figure 3 (left) (more figures are provided in Supporting Information File 1). Despite the core's composition of six electron-rich oxygen atoms, we hypothesized that the bowl-like macrocyclic cavity is electron-deficient in nature owing to the presence of three electron-deficient iodine atoms within the macrocycle's framework. To better understand the electrophilic environment of the macrocyclic cavity, we have calculated the Mulliken charges on each atom of Phe HIM **1**. To analyze the resultant charge of the macrocycle’s cavity, we considered the charge distribution on three iodine atoms, three carbonyl carbon atoms and six oxygen atoms that constitute a small cavity within the macrocycle. The Mulliken charge distribution diagram of HIM **1** (Figure 3, middle) shows that the summation of the overall charge on three oxygen atoms facing towards the center of the macrocycle is somewhat lower than the summation of charge of the three iodine atoms. The resultant charge calculated from three iodine, three carbonyl carbons and six oxygen atoms constituting HIM cavity is 1.994 e, suggesting that the central core of the macrocycle is electrophilic in nature. In addition, DFT results show the three outwardly projected carbonyl oxygens (formerly the carbonyl of benzoic acid) are negatively charged and can potentially bind or interact with metal cations. Figure 3 (right) shows the calculated electrostatic potential map with the area of the electrophilic cavity of HIM (shown in red) being smaller, which suggests lower electron density. In contrast, the red areas of the three outwardly projected carbonyl oxygens (formerly the carbonyl of benzoic acid) are larger, demonstrating the higher overall electron density of that region. Additional information of the electrostatic potential map with color code is provided in the supporting information (Supporting Information File 1, Figure S14).

We recently reported that anions of tetrabutylammonium salts such as F^−^, Cl^−^, Br^−^, and CN^−^ disrupt the secondary bonding in the HIMs and initiate the disassembly of the HIM trimer into a HIM monomer [18]. The HIMs were found not to interact with tetrabutylammonium nitrate, signifying a lack of association between the HIM and the bulky cation (tetrabutylammonium) or the non-nucleophilic anion (nitrate). With these observations, and the previous report of possible cation binding via mass spectrometry experiments [17], we were intrigued by the potential of HIM binding smaller cations such as lithium and sodium. While the previous authors suggested metal cations bind at the oxygen-rich core in a similar fashion to crown ethers, our hypothesis diverged and considered alternative binding sights including the exterior of the HIMs. Recently, Huber and co-workers [21] showed hypervalent iodine(III) compounds were not impacted by non-coordinating BArF cations. Upon this inspiration, we employed LiBArF_20_ and NaBArF_24_ as salt sources to investigate the association of HIMs with metal cations.

**Figure 4:**
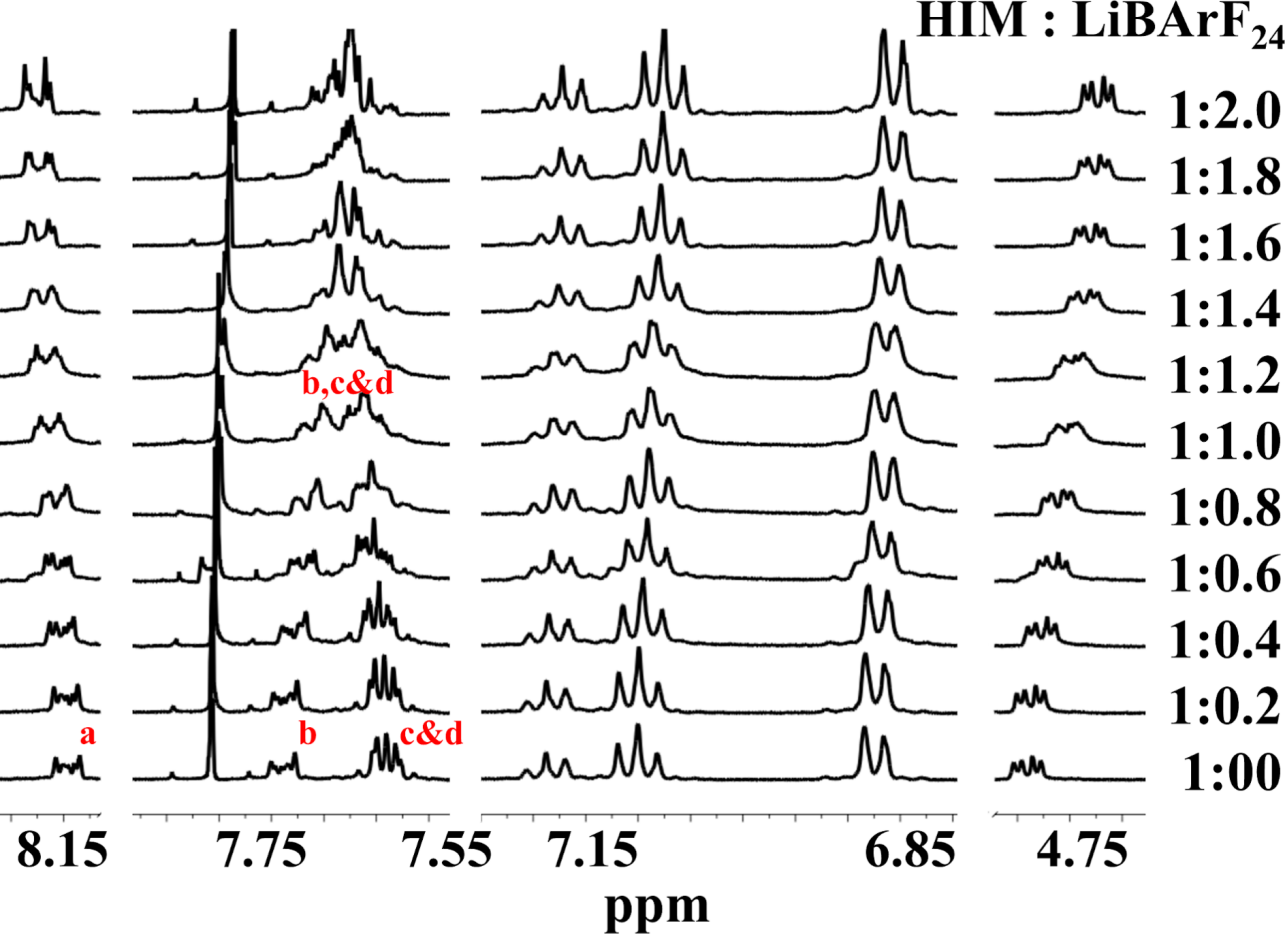
^1^H NMR titration experiment of **1** with LiBArF_20_ at an incremental equivalency in CDCl_3_ and (CD_3_)_2_CO 1:2.

NMR titration of HIM **1** with LiBArF_20_ led to distinct shifting of signals with the successive addition of salt. Figure 4 shows a titration of **1** with increasing equivalents of LiBArF_20_. The most dramatic changes included the aromatic proton multiplet at 7.750 ppm (compound **1**, proton b, Figure 3A) shifting upfield and the multiplet at 7.60 ppm (protons c and d, Figure 3A) shifting downfield and merging into a single multiplet when approximately 1:1 ratio of HIM to salt was added as shown in Figure 4. Additional titration data are provided in Supporting Information File 1, Figures S4 and S5). Similar, yet less dramatic shifts were observed for the other aromatic and aliphatic protons. Alternatively, titration with NaBArF_24_ showed much smaller, nonetheless measurable, movement in the aromatic proton signals upon equivalent salt additions (see Supporting Information File 1, Figures S6 and S7). These results suggest that there are new associative processes occurring in the presence of both LiBArF_20_ and NaBArF_24_. The NMR spectral changes observed are mainly due to the binding of metals (lithium and sodium) to HIM **1**. Additionally, some of these changes could be attributed to minor interactions with the weekly coordinating counter ions BArF_20_^−^ or BArF_24_^−^. The larger magnitude of changes with the addition of LiBArF_20_ in comparison to NaBArF_24_ suggests a stronger association and is presumably owing to the difference in size and bonding strength of the cations.

**Figure 5:**
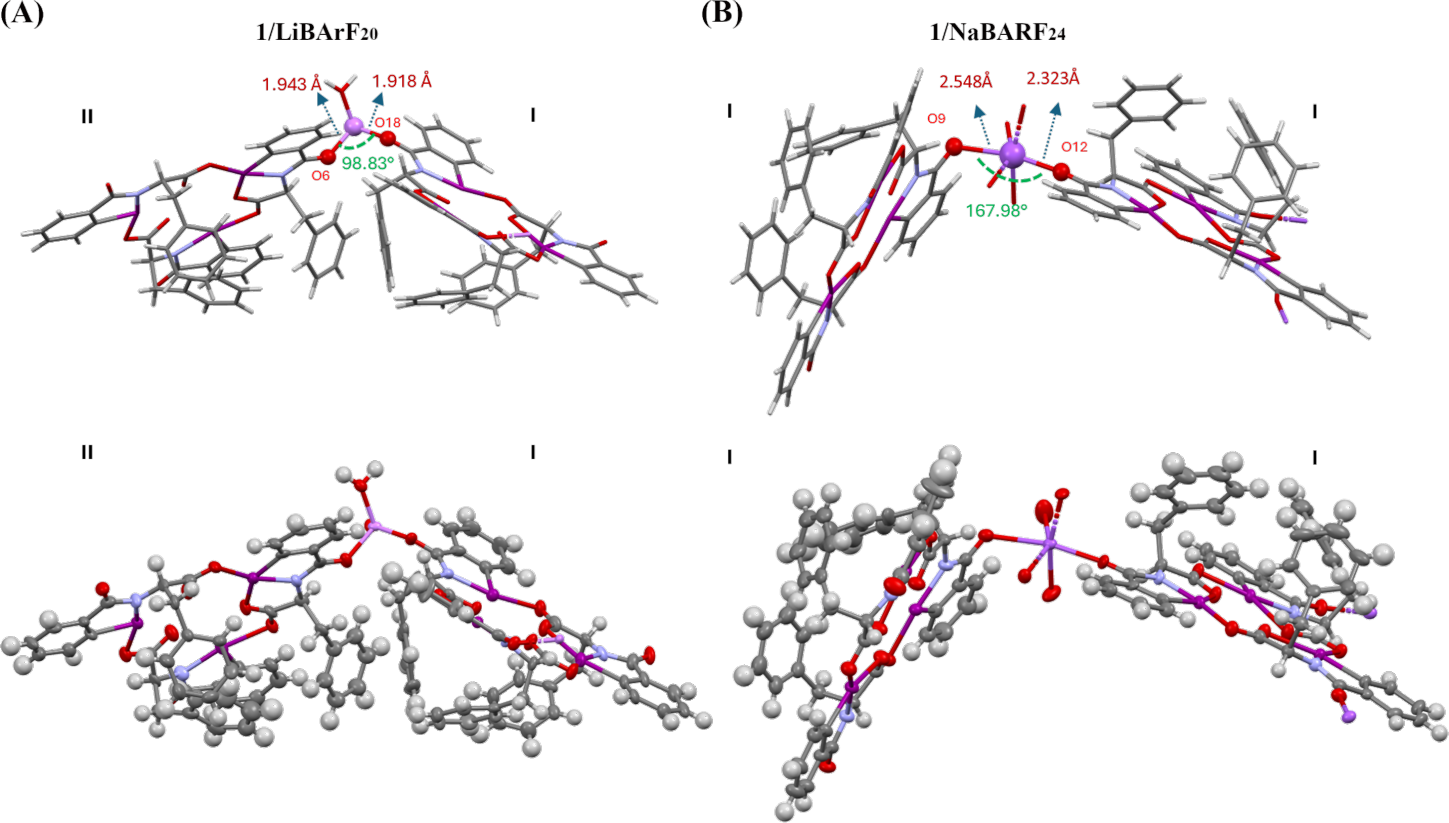
Crystal structures of HIM **1** and LiBArF_20_ (A) and NaBArF_24_ (B). BARF cation is omitted for clarity. Nitrogen, oxygen, iodine, and metal (lithium or sodium) atoms are denoted by light blue, red, purple, and lavender color, respectively. Thermal ellipsoids drawn at 50% probability.

**Figure 6:**
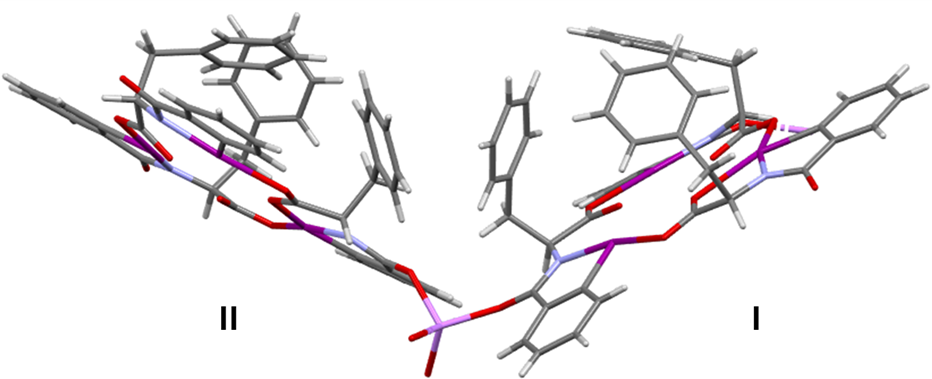
Alternative view of the crystal structure of the HIM **1** and LiBArF_20_ complex. BArF_20_ anion is omitted for clarity. Nitrogen, oxygen, iodine, and lithium atoms are denoted by light blue, red, purple, and lavender color, respectively. Conformer **II** displays two benzyl groups projected in and one benzyl group projected out. Conformer **I** displays all benzyl groups projected towards the interior of the macrocycle.

To further understand the complexation with metal cations, separate co-crystals of HIM **1** with LiBArF_20_ and NaBArF_24_ were obtained by vapor diffusion of diethyl ether into acetone. The details of the co-crystallization experiment are provided in the crystallographic information section of Supporting Information File 1. Single crystals with quality suitable for XRD analysis were obtained in both cases (Figure 5). Unlike the previous assumption of metal coordination at the macrocycle core [17], the crystal structures reveal complexation of both cations at the periphery of the macrocycle. Both **1**/LiBArF_20_ and **1**/NaBArF_24_ crystalized in an orthorhombic system with a *P*212121 space group, where the unit cell of each complex contains two molecules of HIM for every metal BArF_20_ or BArF_24_. Details of the crystal structure data set can be found in Supporting Information File 2. The overall macrocycle structures are analogous to the single crystal structure of the previously reported phenylalanine HIMs alone (Figure 2). For the **1**/LiBArF_20_ crystal, the phenylalanine HIMs exists in two different molecular conformations analogous to **I** and **II** (Figure 6) with differences arising in the location of the projected benzyl groups. For the **1**/NaBArF_24_ complex, the repeating unit shows that both phenylalanine HIMs exhibit the same conformation (e.g., **I**) where all three benzyl groups are oriented toward the interior of the macrocycle (Supporting Information File 1, Figure S9).

From the single crystal data set, we observed that a lithium ion is coordinated with two variations of the phenylalanine macrocycles (**I** and **II**) through the outwardly projected carbonyl oxygens (O6 from **I** and O18 from **II**) via oxygen–metal bonds (Figure 5). The bond distance between O6–Li is 1.943 Å, which is slightly longer than the bond distance between O18–Li of 1.918 Å. The overall structure is a bent dimer with the two phenylalanine HIM structures arranged in close geometry with a O6–Li–O18 bond angle of 98.83°. In contrast, the Na^+^-incorporated co-crystal structure is different than the Li^+^ co-crystal, even though the unit cell is almost the same. In this complex, the Na^+^ is similarly coordinated with two phenylalanine macrocycles through the amide moiety carbonyl oxygen (O9 from one **I** and O12 from second **I**). However, the bond distances between oxygen and the metal are significantly longer for Na^+^ versus Li^+^. The O9–Na distance is 2.548 Å while the O12–Na is 2.323 Å. The average difference in bond lengths between Li^+^ and Na^+^ is ≈0.5 Å. Unlike the Li^+^ complex, the Na^+^ complex grows into a polymer-like network. While the overall structure forms a polymer, the molecular repeating unit consists of two HIM **1** for every NaBArF_24_. Though only the smallest unit of the chain is shown in Figure 5, the ratio of the HIM **1** to NaBArF_24_ remains 2:1 upon applying the same number of symmetry operators to the chain. The Na^+^ bond angles are more linear than Li^+^ with an O9–Na–O12 bond angle of 167.98°.

**Figure 7:**
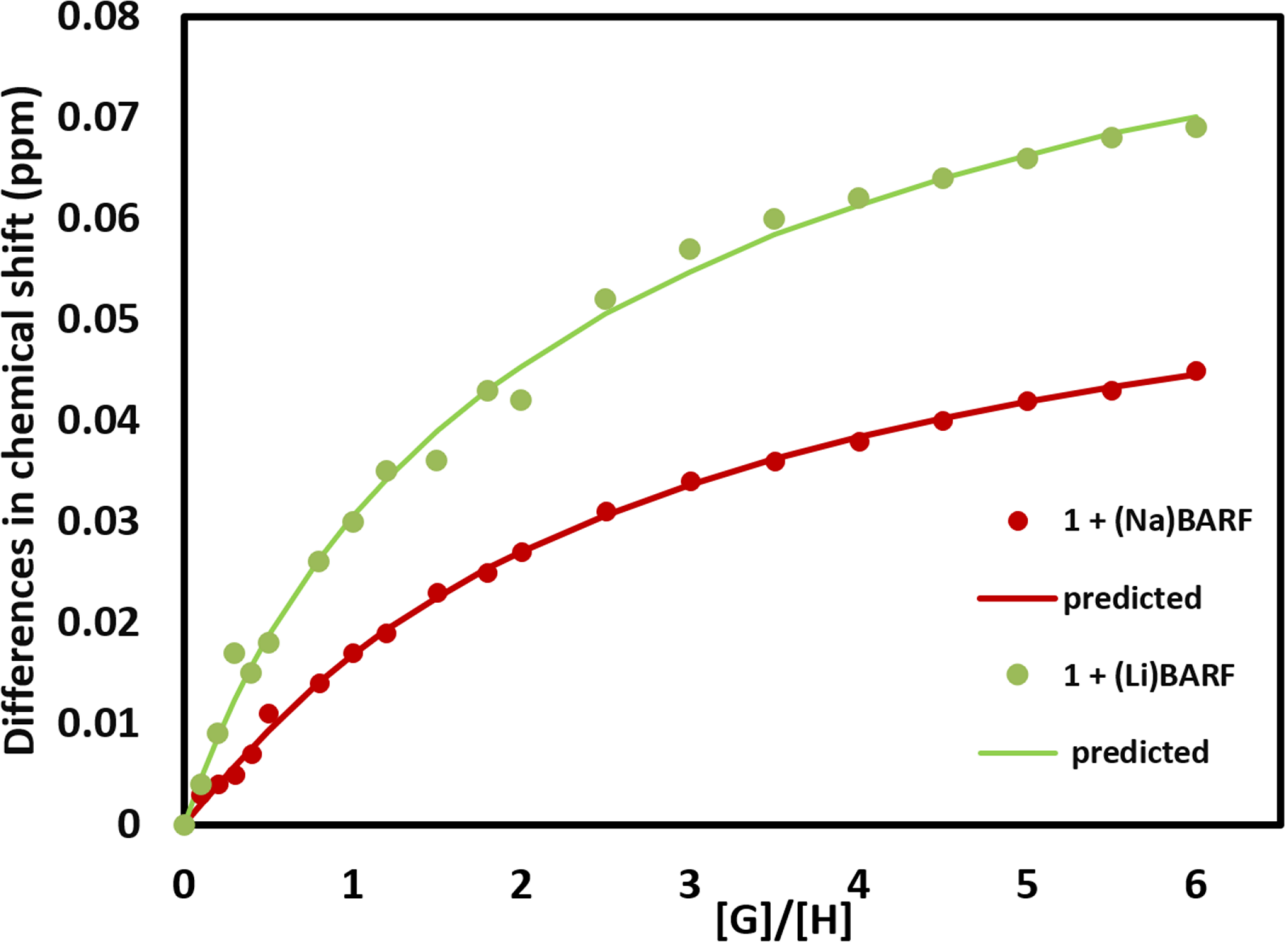
Isotherms of **1** titrated with NaBArF_24_ orLiBArF_20_. The solid lines are the predicted model fits for each curve. [H] is defined as concentration of HIM. The curve is derived using a 2:1 stoichiometric ratio, selected from the BindFit software (http://app.supramolecular.org/bindfit/).

To better understand the association process in this HIM system, the ^1^H NMR titration data was used to determine the association constants for metal coordination. Two stock solutions were prepared with one containing a concentration of 2.83 mM of the macrocycle (HIM) in a mixture of deuterated chloroform and acetone 1:2. The second stock solution contained the metal BArF_20_ or BArF_24_ at a concentration of 10 times that of the HIM concentration. Gradually adding the metal BArF_20_ or BArF_24_ stock solution to the HIM stock solution in an NMR tube allowed the host-to-guest ratio to be varied while keeping the host concentration constant. With crystallographic confirmation of a 2:1 host–guest complex, titration data was fitted using a 2:1 model of HIM to metal BArF_20_ or BArF_24_ (Figure 7) [22–23]. The calculated cooperative association constants for **1** with LiBArF_20_ are 0.09 M^−1^ and 21522 M^−1^. The cooperative association constants for HIM with NaBArF_24_ were found to be considerably lower with associations of 68 M^−1^ and 115 M^−1^. The estimated errors in the cooperative binding constants for Li are 5% and 38%, while for Na, the errors are 13% and 29%. These errors are asymptotic errors at the 95% confidence interval level [24] and could be due to the fluctuation of the concentration of the host and the guest, which contribute to the *x*-axis in the fitting process. The isothermal fitting to 2:1 models is often prone to overfitting with NMR data and is noticeable with the fit for **1**. However, the general trend does correlate with the strength of the respective metal–oxygen bonds.

**Figure 8:**
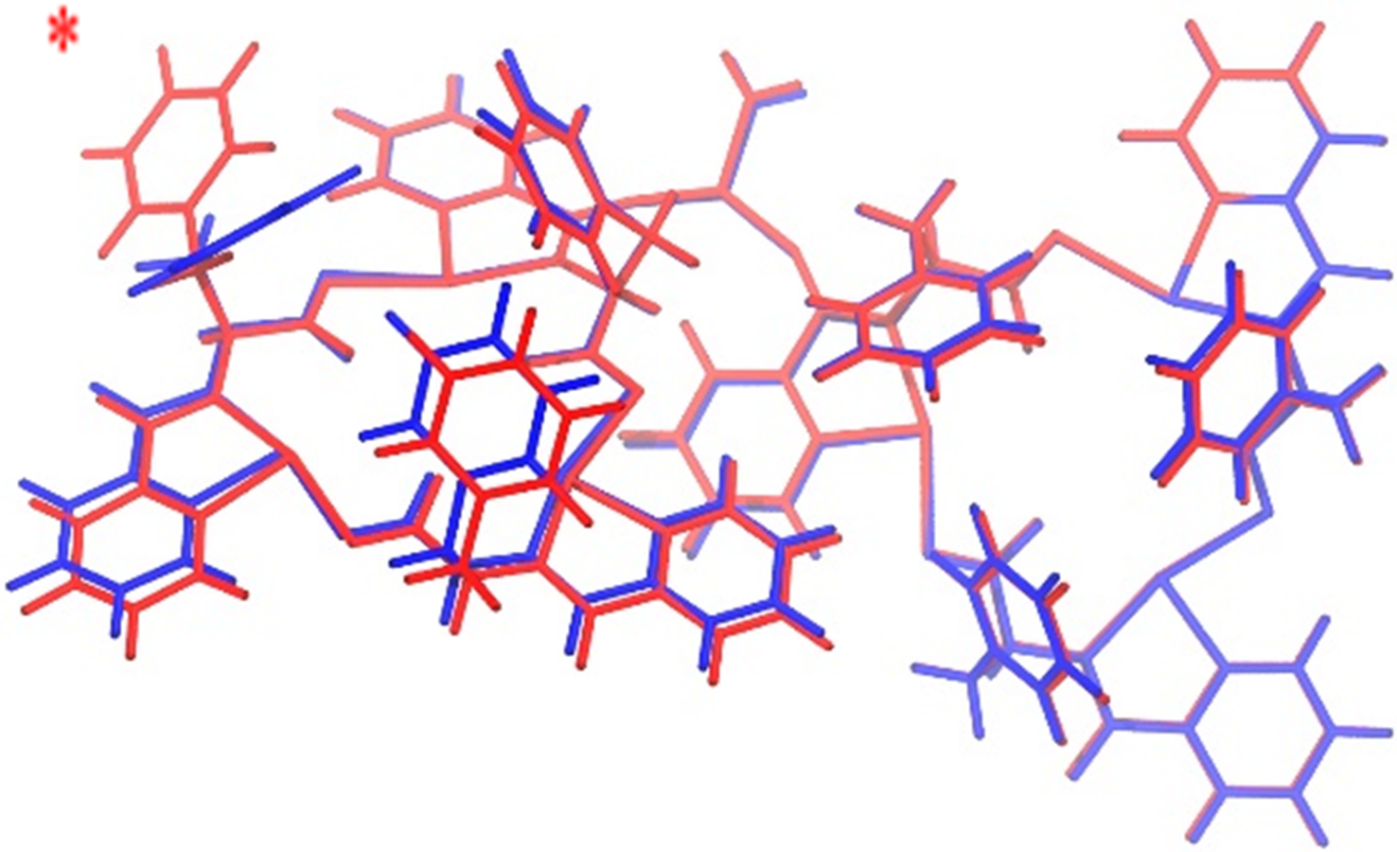
Lithium complex **2** (red) overlaid with lithium complex **3** (blue). In lithium complex **2**, one benzyl ring is pointing outside highlighted by the red-colored asterisk.

The relatively high *K*_a_ value seen for the Li^+^ as compared to Na^+^ cation is presumed to result from difference in atom size. The Li^+^, being smaller in size, binds more strongly with HIM than the Na^+^ resulting in the higher binding constants. To gain further insight into the difference in binding constants as well as the isolation of two distinct molecular conformations of phenylalanine HIM **1** (Figure 1), DFT calculations were performed. The details of DFT calculations are shown in Table 1. For DFT calculations, we assigned two HIM macrocycles per LiBArF_20_ as found in the co-crystal. Li^+^ complex **2** (Figure 8) represents the complex consisting of HIM in two different molecular conformations **I** and **II**, whereas Li^+^ complex **3** (Figure S19 in Supporting Information File 1) represents a complex consisting of both macrocycles in the same molecular conformation (e.g., **I** and **I**). Similarly, Na^+^ complex **4** (Figure S20, Supporting Information File 1) represents the complex consisting of HIM in two different molecular conformations **I** and **II**, whereas the Na^+^ complex **5** (Figure S21, Supporting Information File 1) represents a complex consisting of both HIM in same molecular conformation, **I** and **I**. Without any metal coordination, DFT suggests conformer **I** is more stable than conformer **II** by ≈60 kJ/mol. However, upon complexation with Li^+^, both complexes **2** and **3** are remarkably similar in energy with the difference of less than 1 kJ/mol. This very small energy difference should be too small for marked differences in the arrangement of groups in space. The overlaid image of lithium complexes **2** and **3** (Figure 8) shows the similar arrangement of all groups on plane except for one benzyl ring (highlighted by a red asterisk) in complex **2** that faces outside the interior of the macrocycle. A similar trend was seen in the HIM–Na^+^ crystals where complex **4** and complex **5** are similar in energy with a difference of less than 1.5 kJ/mol. While both Li^+^ and Na^+^ crystals were prepared in similar conditions, the difference in the resulting crystal structure (e.g., location of benzyl rings) could arise from a difference in the overall nucleation events of a given crystal and not necessarily from a difference in energy between conformations. DFT results of the binding energy of the two metals shows that the isolated Li^+^ complex **2** (1029.87 kJ/mol) is more stable than the Na^+^ complex (897.77 kJ/mol). This significant energy difference supports the observed stronger binding of Li^+^ in these systems. The higher binding constant for the Li^+^ cation is consistent with the correlation between binding constants and binding energies for a 2:1 model [25].

**Table 1:** Binding energies and binding constants for HIM **1** with LiBArF_20_ and NaBArF_24_.

System (conformation)	Binding energy (kJ/mol)	Binding constant (M^−1^)

HIM **1** (**I)**	−297.05	
HIM **1** (**II)**	−237.31	
Li complex **2** (**I** and **II**)	−1029.87	0.09, 21522
Li complex **3** (**I** and **I**)	−1029.69	
Na complex **4** (**I** and **II**)	−896.39	
Na complex **5** (**I** and **I**)	−897.77	68, 115

## Conclusion

In conclusion, we have explored the binding of hypervalent iodine macrocycles with two first group alkali metals. Analysis of association constants reveals that, upon addition of LiBArF_20_ and NaBArF_24_, the Li^+^ binds stronger than Na^+^ cation with the macrocycle. Alternative to a previous report, the metal cations bind to the periphery of the macrocycle and not to the electrophilic, yet oxygen-rich, core of the macrocycle.

## Supporting Information

File 1 Detailed experimental procedures, NMR spectra, and X-ray crystallography details.

File 2 Crystallographic Information Files for **1**/LiBArF_20_ and **1**/NaBArF_24_.

## Data Availability

Data generated and analyzed during this study is available from the corresponding author upon reasonable request.
