# Supplementary material for: Supramolecular assembly of hypervalent iodine macrocycles and alkali metals
Source: Beilstein J Org Chem. 2025 May 30;21:1095–103. doi: 10.3762/bjoc.21.87 (PMC12130623; doi:10.3762/bjoc.21.87)
Supplement: File 1 — Detailed experimental procedures, NMR spectra, and X-ray crystallography details. [file Beilstein_J_Org_Chem-21-1095-s001.pdf]

BEILSTEIN JOURNAL OF ORGANIC CHEMISTRY

Supporting Information

for

Supramolecular assembly of hypervalent iodine macrocycles and alkali metals

Krishna Pandey, Lucas X. Orton, Grayson Venus, Waseem A. Hussain, Toby Woods, Lichang Wang and Kyle N. Plunkett

Beilstein J. Org. Chem. **2025,** *21,* 1095–1103. doi:10.3762/bjoc.21.87

Detailed experimental procedures, NMR spectra, and X-ray crystallography details

License and Terms: This is a supporting information file under the terms of the Creative Commons Attribution License (https://creativecommons.org/licenses/by/4.0). Please note that the reuse, redistribution and reproduction in particular requires that the author(s) and source are credited and that individual graphics may be subject to special legal provisions.
The license is subject to the *Beilstein Journal of Organic Chemistry* terms and conditions: (https://www.beilstein-journals.org/bjoc/terms)

Table of contents

1. Crystal structure of HIM **1** S2
2. Cation binding experiment setup and evaluation........ S3
3. NMR titrations S5
4. Crystallographic information S9
5. Computational experiments S138
6. References S171

Crystal structures of previously isolated phenyl HIM 1

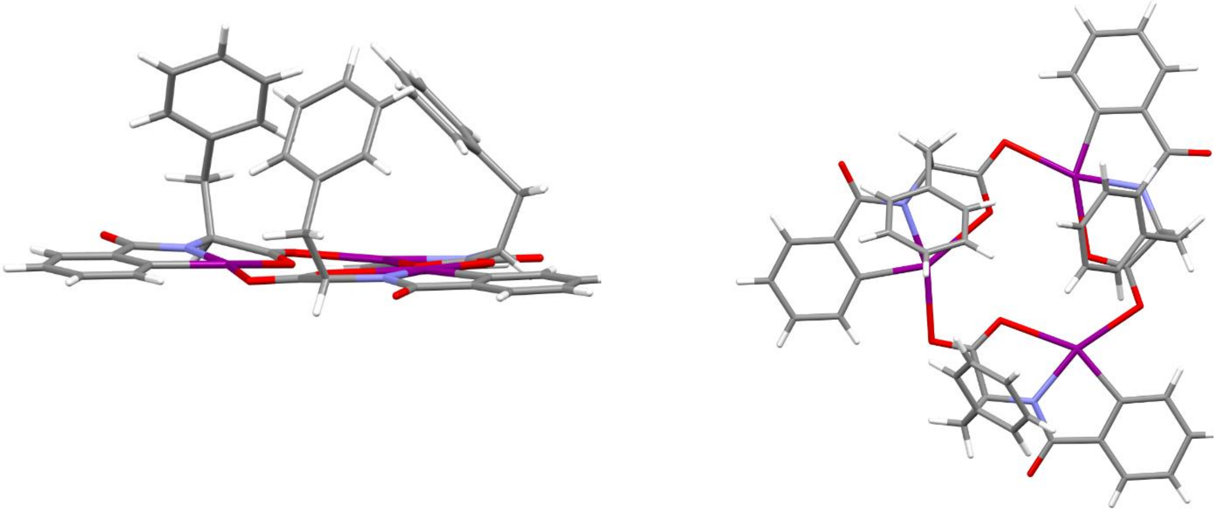

Figure S1. Expanded representation of crystal structure of HIM **1**. Left) Clear representation displaying all benzyl groups located above a single plane. Right) Clear representation showing carbonyl carbon facing towards the interior of the macrocycle. Oxygen, nitrogen and iodine atoms are denoted by red, light blue and purple color respectively. Crystal structure from our previous manuscript.[1]

Binding experiments

Analogous to the binding experiment reported in our previous study,[1] the binding ability between the phenylalanine hypervalent iodine macrocycle (HIM) **1** and lithium as well as sodium cations was primarily analyzed utilizing ^{1}H NMR spectrometry in deuterated chloroform/acetone 1:2. From the ^{1}H NMR study, the data was used to determine the binding constant between the HIM and cations in Bindfit (http://app.supramolecular.org/bindfit/). HIM was treated with various two cations Li^+ and Na^+. Commercially available lithium tetrakis(pentafluorophenyl)borate ethyl etherate ($LiBArF_{20}$) and sodium tetrakis[3,5-bis(trifluoromethyl)phenyl] borate ($NaBArF_{24}$) were used for the experiment.

In a similar manner to our previously reported NMR titration experiment,[1] an incremental equivalent addition of metal $BArF_{20}$ or $BArF_{24}$ to the HIM was performed for this experiment. Stock solution (A) of HIM **1** was prepared at a concentration of 2.83 mM which was kept constant throughout the process. A portion of HIM stock was used to prepare salt stock (B) at an equivalence of 10 times. 0.5 mL of stock A was taken to the NMR tube and the spectra ^{1}H NMR spectrum was recorded.

For $LiBArF_{20}$

Then stock B was added stepwise in 0.1 equiv (versus the HIM) increase until a 1:1.5 equiv of HIM to $LiBArF_{20}$ was added. After each stepwise addition of stock B to the NMR tube of stock A, a ^{1}H NMR spectrum was recorded. The incremental rate increased to 0.2 or 0.3 equiv from 0.5 to 2 equivalents. After 2 equiv of stock B were added to the NMR tube the increment rate was kept at 0.5 equiv for each addition till the last addition.

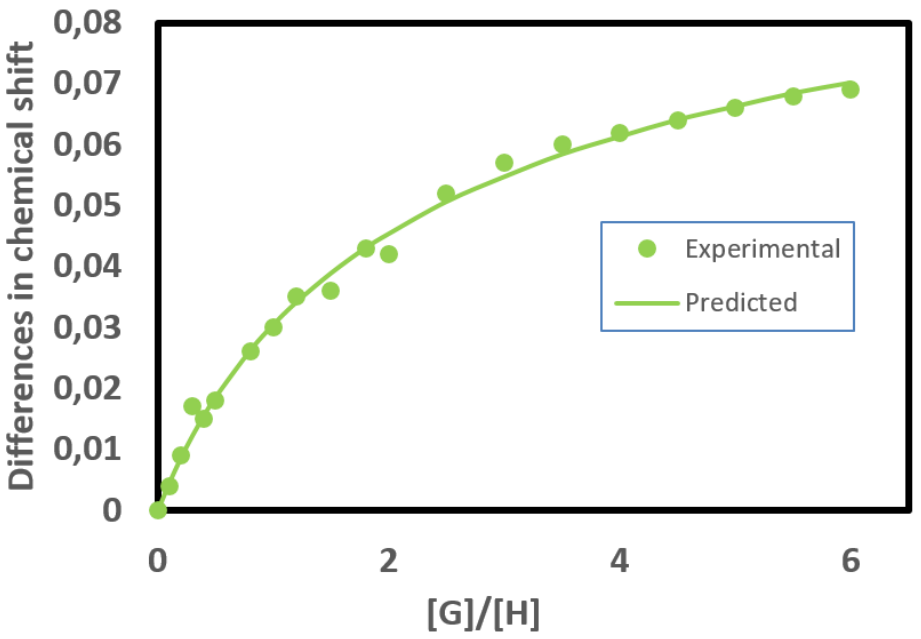

Figure S2. Binding isotherms of Phe-HIM **1** with titrating equivalents of $LiBArF_{20}$. The curve is derived using a 2:1 stoichiometric ratio for HIM **1** and Li, selected from the BindFit software (http://app.supramolecular.org/bindfit/). This 2:1 model was chosen for the BindFit experiment based on the evidence of an isolated co-crystal, which demonstrated a 2:1 ratio between HIM **1** and Li metal.

For $NaBArF_{24}$

Then stock B was added stepwise in 0.1 equiv (verses the HIM) increase until a 1:1.3 equiv of HIM to $NaBArF_{24}$ was added. After each stepwise addition of stock B to the NMR tube of stock A, a ^{1}H NMR spectrum was recorded. The incremental rate increased to 0.2 or 0.3 equiv from 1.3 to 2 equiv. After 2 equiv stock B was added to the NMR tube the increment rate kept at 0.5 equiv for each addition till the last addition.

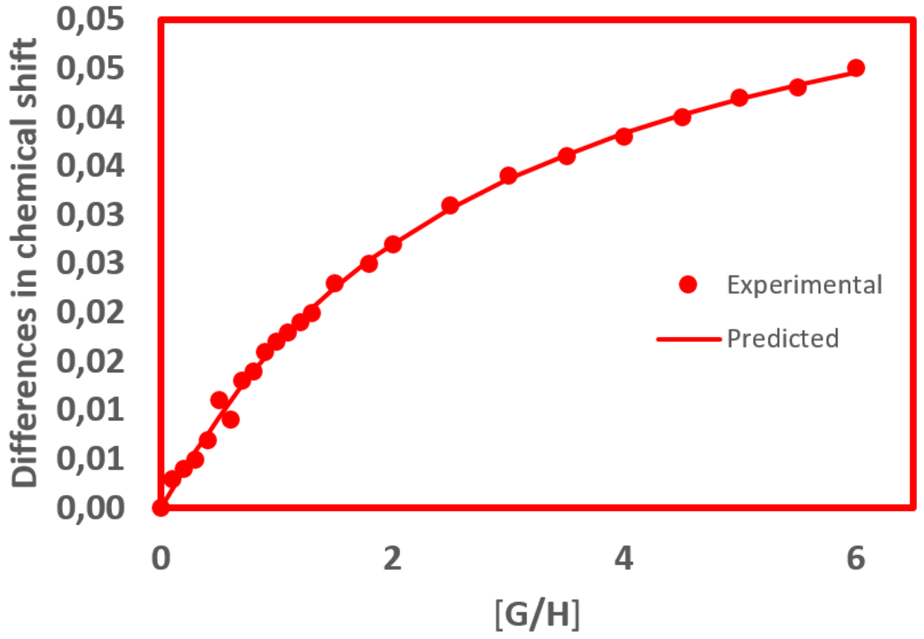

Figure S3. Binding isotherms of Phe-HIM **1** with titrating equivalents of $NaBArF_{24}$. The curve is derived using a 2:1 stoichiometric ratio for HIM **1** and Na, selected from the BindFit software (http://app.supramolecular.org/bindfit/). This 2:1 model was chosen for the BindFit experiment based on the evidence of an isolated co-crystal, which demonstrated a 2:1 ratio between HIM **1** and Na metal.

NMR Titrations

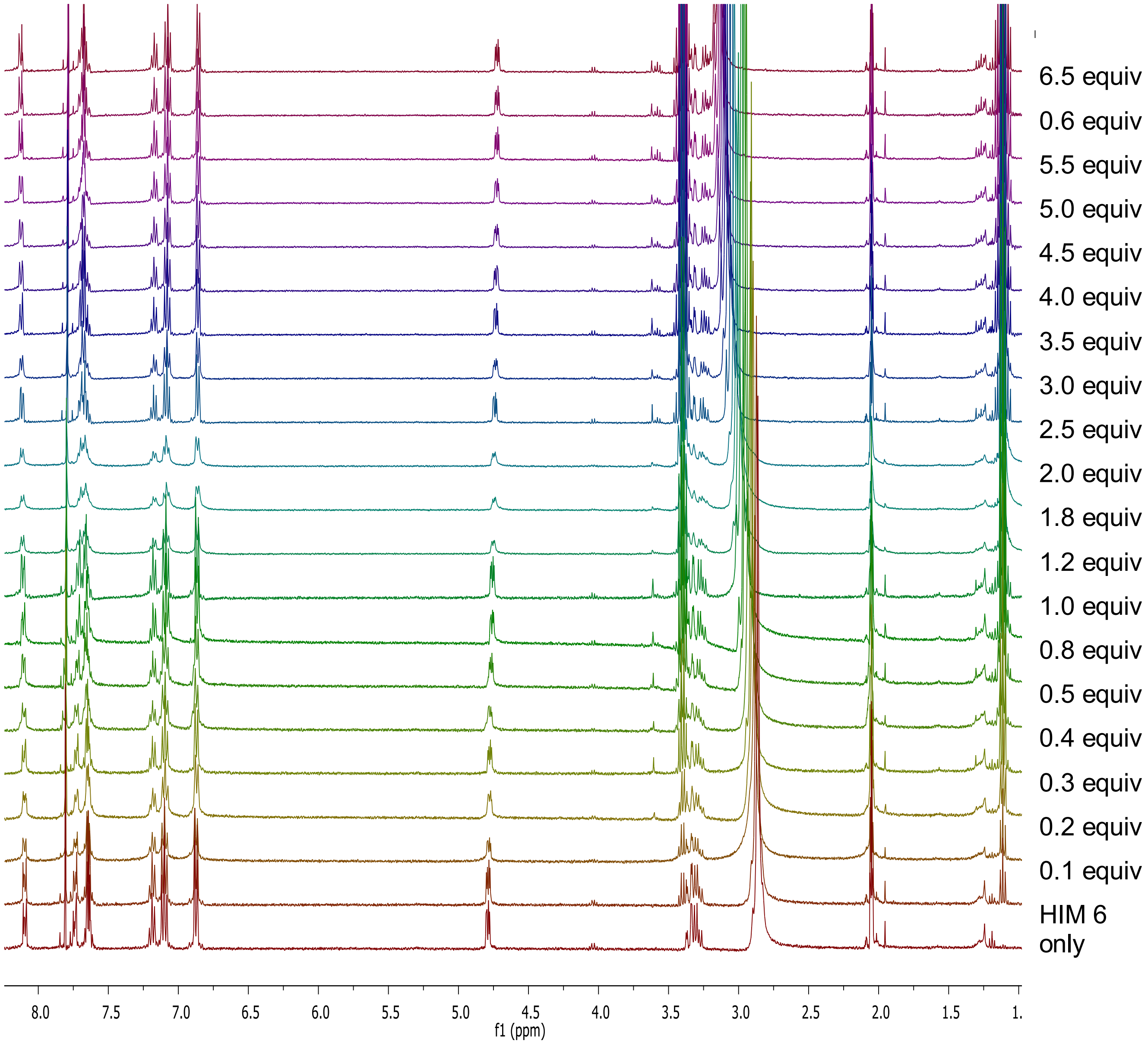

Figure S4. ^{1}H NMR titration of **1** with $LiBArF_{20}$ at an incremental equivalency, starting from 0.1 to 6.5 $LiBArF_{20}$ in $(CD_3)_2CO/CDCl_3$(2:1). See Figure S5 for the expansion of aromatic region.

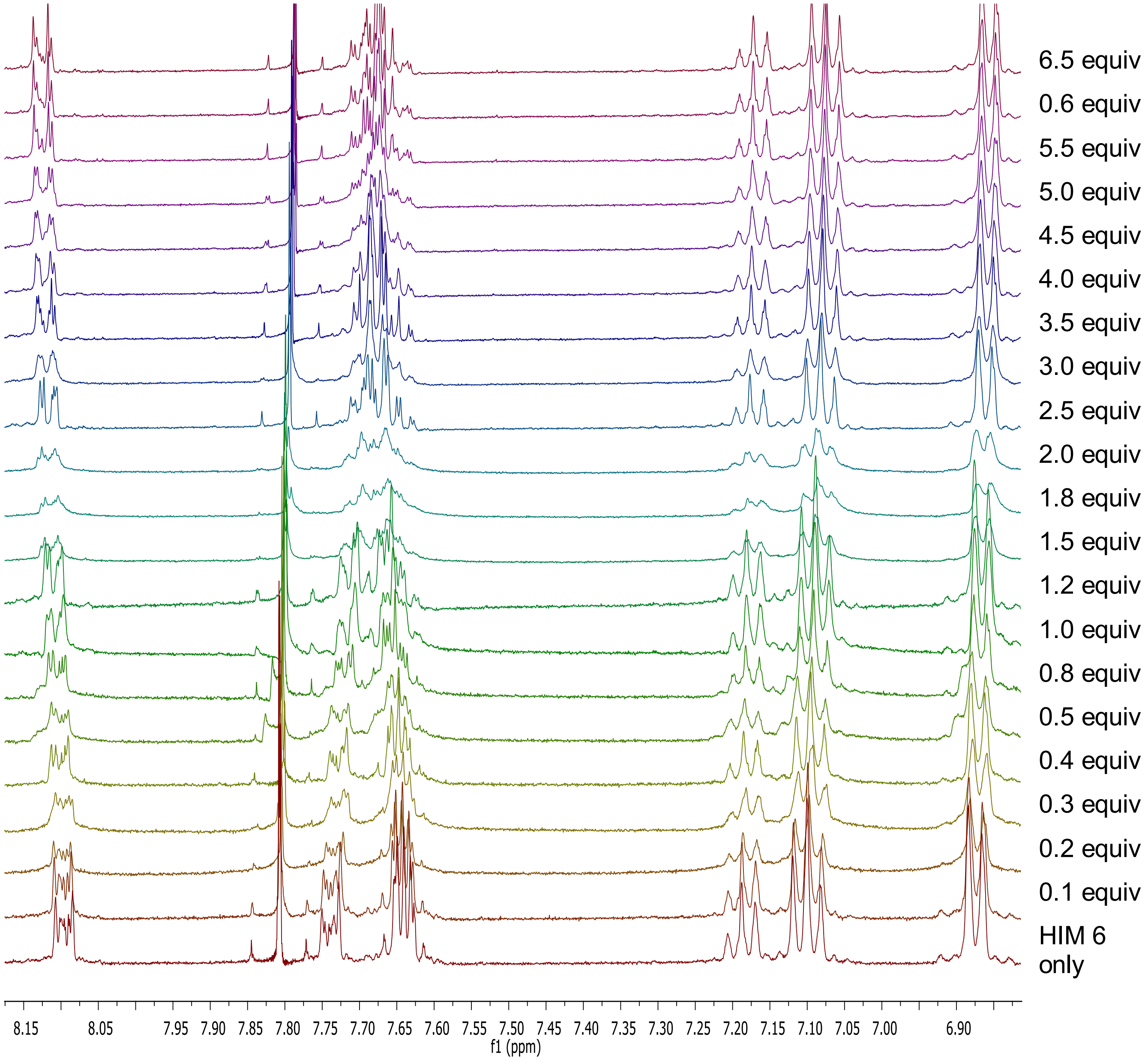

Figure S5. Expanded representation of aromatic region of ^{1}H NMR titration of **1** with $LiBArF_{20}$ at an incremental equivalency, starting from 0.1 to 6.5 $LiBArF_{20}$ in $(CD_3)_2CO/CDCl_3$(2:1). The miltiplet initially detected at 7.750 ppm shifted up field and completely merged with adjacent multiplet initially observed at 7.652 ppm. These two aromatics multiplets merged into a single multiplet with the addition of approximately 1.8 equivalents of $LiBArF_{20}$.

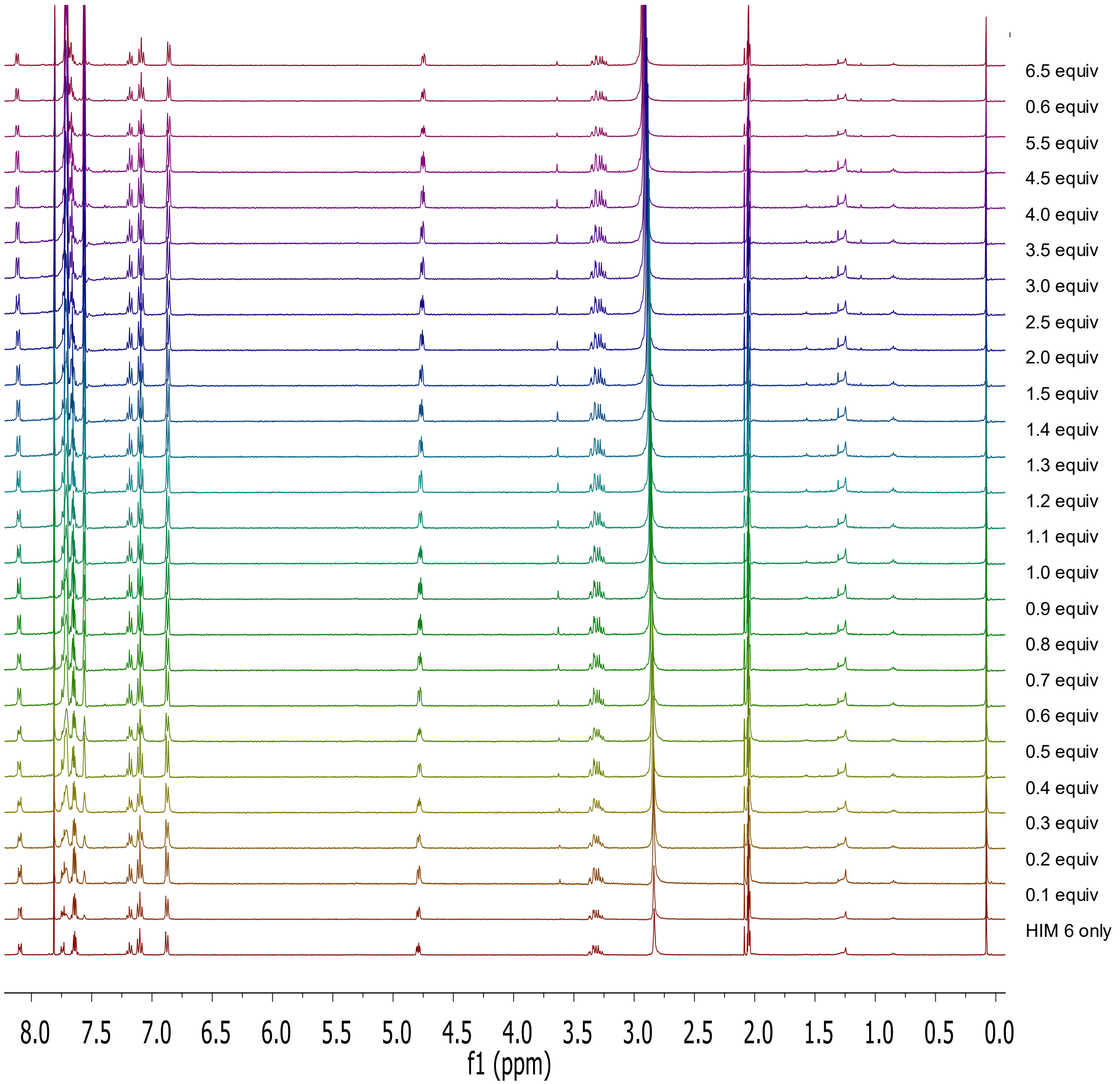

Figure S6. ^{1}H NMR titration of **1** with $NaBArF_{24}$ at an incremental equivalency, starting from 0.1 to 6.5 $NaBArF_{24}$ in $(CD_3)_2CO/CDCl_3$(2:1). Very small changes in the shift of both aromatic and aliphatic signals was observed. The aromatic signal at 8.083 ppm slightly shifted downfield to 8.109 ppm. Another aromatic signal initially detected at 6.886 ppm shifted to 6.869 ppm and the aliphatic signal at 4.805 ppm shifted to 4.734 ppm. See Figure S7 for the expansion of aromatic region.

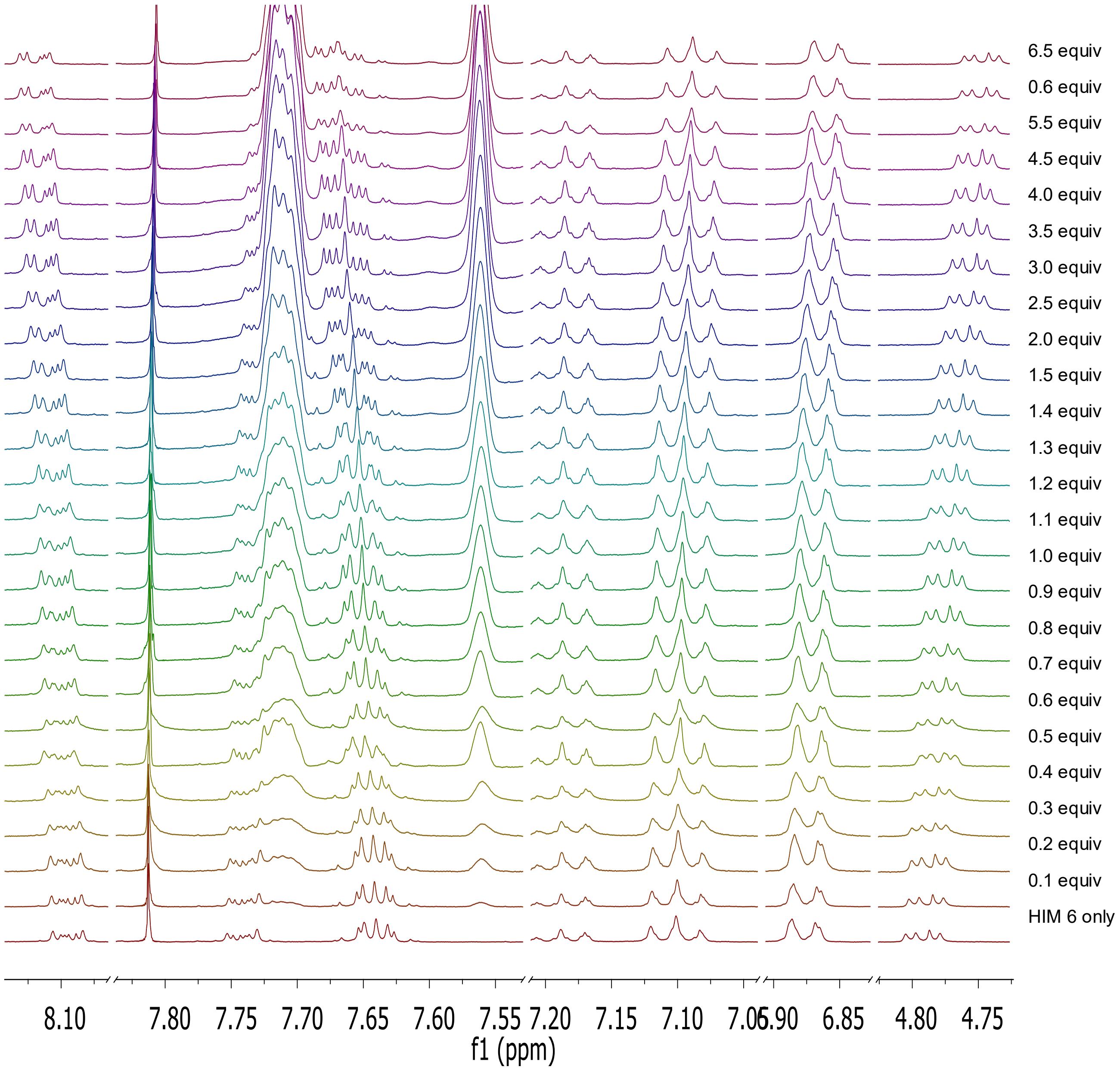

Figure S7. Expanded representation of ^{1}H NMR titration of **1** with $NaBArF_{24}$ at an incremental equivalency, starting from 0.1 to 6.5 $NaBArF_{24}$ in $(CD_3)_2CO/CDCl_3$(2:1). Very small changes in the shift of both aromatic and aliphatic signals takes place. The aromatic signal at 8.083 ppm slightly shifted downfield to 8.109 ppm. Another aromatic signal initially detected at 6.886 ppm shifted to 6.869 ppm and the aliphatic signal at 4.805 ppm sifted to 4.734 ppm.

Crystallographic information

Intensity data were collected on a Bruker D8 Venture kappa diffractometer equipped with a Photon II CPAD detector. An Iμs microfocus Mo source (λ = 0.71073 Å) coupled with a multi-layer mirror monochromator provided the incident beam. The sample was mounted on a 0.3 mm nylon loop with the minimal amount of Paratone-N oil. Data was collected at 100 K using a cold stream of N_2(g) provided by an Oxford Cryostream 700 LT device. Preliminary unit cell constants were determined with a set of 24 narrow frame scans. Data was collected as a series of φ and/or ω scans with typical scan width of 0.5° and counting time of 10–30 seconds/frame at a crystal to detector distance of ≈ 3.7 cm. The collection, cell refinement, and integration of intensity data was carried out with the APEX3 software.[2] Final cell constants were determined by global refinement of reflections from the complete data set. A multi-scan absorption correction was performed with SADABS.[3] The structure was phased with intrinsic methods using SHELXT[4] and refined with the full-matrix least-squares program SHELXL.[5]

(1) Pandey, K., Arafin, S., Jones, E., Du, Y., Kulkarni, G.C., Uddin, A., Woods, T.J., Plunkett, K.N., J. Org. Chem. 2024, 89 (11), 7437-7445

(2) Bruker (2018). APEX3. Bruker AXS, Inc., Madison, Wisconsin, USA.

(3) Krause, L., Herbst-Irmer, R., Sheldrick, G. M. and Stalke, D. J. Appl. Cryst., 2015, 48, 3-10.

(4) Sheldrick, G. M. Acta Cryst. 2015, A71, 3-8.

(5) Sheldrick, G. M. Acta Cryst. 2015, C71, 3-8.

Phenylalanine HIM/LiBArF20 crystal, CCDC number 2390610

The structure was confirmed by obtaining a single crystal that was solved using X-ray diffraction. The single crystal of phenylalanine HIM/LiBArF20 co-crystal suitable for X-ray crystallography was obtained using acetone and diethyl ether by vapor diffusion method. A co-crystal of HIM **1** with LiBArF20 was prepared through vapor diffusion method by introducing 5 mL of diethyl ether into the solution of 1 mL of acetone containing HIM **1**(10 mg, 0.0084 mmol) and LiBArF20 (7.3 mg, 0.0084 mmol). The single crystal was run at 100 K using a wavelength of 0.71073 A°. From the analysis of single crystal data set, we observed that a lithium atom is coordinated to two phenylalanine macrocycles through carbonyl oxygen forming a metal coordinated hypervalent iodine based macrocyclic complex. Analogous to the single crystal structure of phenylalanine HIM, two phenylalanine HIM in this complex are also distorted planner macrocyclic system consisting of the amino acids carbonyl oxygen facing inside the ring and the benzyl group facing above the plane of the macrocycle. It is important to note that phenylalanine HIM exists in two molecular conformations in this complex. In conformer I, the structure includes two benzyl groups projecting inward and a benzyl group projecting outward from the macrocycle. In contrast, conformer II features all three benzyl groups oriented towards the interior of the macrocycle.

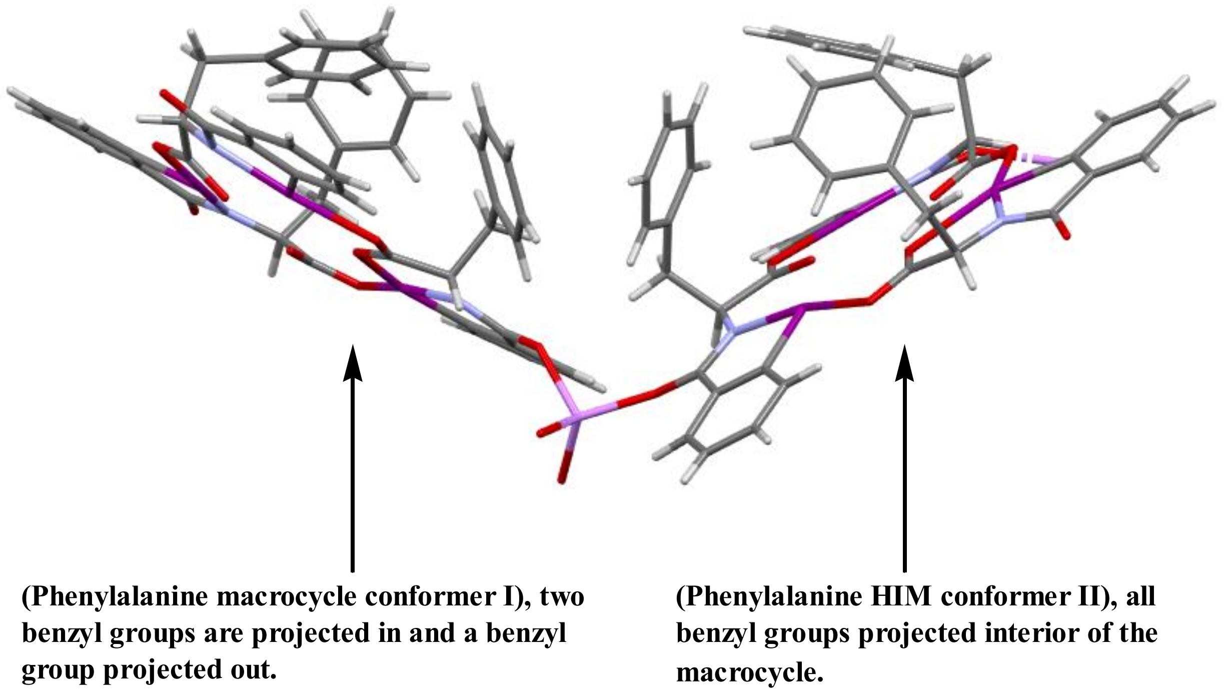

Figure S8. Perspective view of crystallographically independent molecules of phenylalanine HIM/LiBArF20 complex. Solvent is removed for clarity. Nitrogen, oxygen, iodine, and lithium atoms are denoted by light blue, red, purple, and lavender color respectively. Hydrogens atoms are omitted. (Capped sticks style)

Phenylalanine HIM/NaBArF24 crystal, CCDC number 2390609

The single crystal of Phenylalanine HIM **1** /NaBArF$_{24}$ co-crystal suitable for X-ray crystallography was obtained using acetone and diethyl ether by vapor diffusion method. The single crystal was run at 100 K using a wavelength of 0.71073 A°. A co-crystal of HIM **1** with NaBArF$_{24}$ was prepared through vapor diffusion method by introducing 5 mL of diethyl ether into the solution of 1 mL of acetone containing HIM **1** (10 mg, 0.0084 mmol) and NaBArF$_{24}$ (7.5 mg, 0.0084 mmol). From the analysis of single crystal data set, analogous to phenylalanine HIM/LiBArF$_{20}$ crystal, we observed that a sodium atom is coordinated to two phenylalanine macrocycles through carbonyl oxygen forming a metal coordinated hypervalent iodine based macrocyclic complex. Similar to the single crystal structure of phenylalanine HIM, two phenylalanine HIM in this complex are also distorted planner macrocyclic system consisting of the amino acids carbonyl oxygen facing inside the ring and the benzyl group facing above the plane of the macrocycle. Each phenylalanine HIM exhibits all three benzyl groups oriented toward the interior of the macrocycle, as depicted in the figure below.

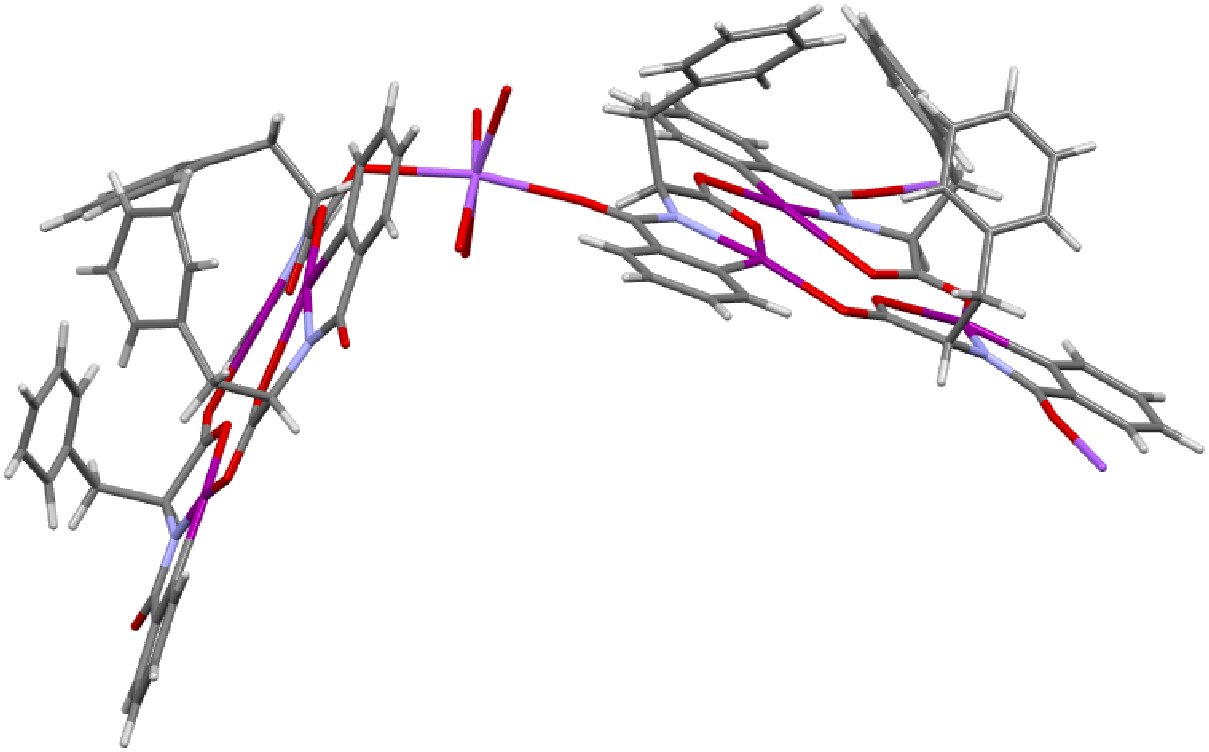

Figure S9. Perspective view of crystallographically independent molecules of phenylalanine HIM **1** /NaBArF$_{24}$ complex. Solvent is removed for clarity. Nitrogen, oxygen, iodine and sodium atoms are denoted by light blue, red, purple and lavender color respectively. Hydrogens atoms are omitted. All three benzyl groups in both phenylalanine HIMs are projected towards the interior of the macrocycle. (Capped sticks style)

University of Illinois, Department of Chemistry

George L. Clark X- R Facility and 3M Materials Laboratory

Structure report for compound Phenylalanine HIM/LiBArF$_{20}$

Identification code	2390610
Empirical formula	C126.80 H91 B F20 I6 Li N6 O20.70
Principal Investigator:	Professor Dr. Kyle N Plunkett

List of Tables

Table 1. Crystallographic Experimental Details

Table 2. Atomic Coordinates and Equivalent Isotropic Displacement Parameters

Table 3. Selected Interatomic Distances and Selected Interatomic Angles

Table 4. Anisotropic Displacement Parameters

Table 5. Derived Atomic Coordinates and Displacement Parameters for Hydrogen Atoms

Table 6. Torsional Angles

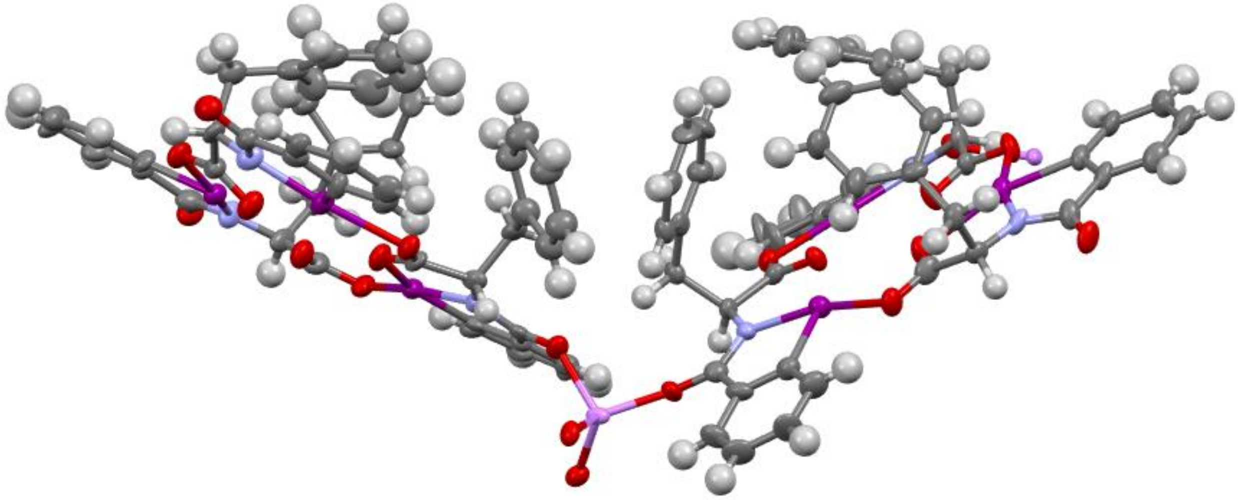

Figure S10. Perspective view of crystallographically independent molecules of phenylalanine HIM **1**/$LiBArF_{20}$ complex. Solvent is removed for clarity. Nitrogen, oxygen, iodine and lithium atoms are denoted by light blue, red, purple and lavender color respectively. Hydrogens atoms are omitted. (Ellipsoid style)

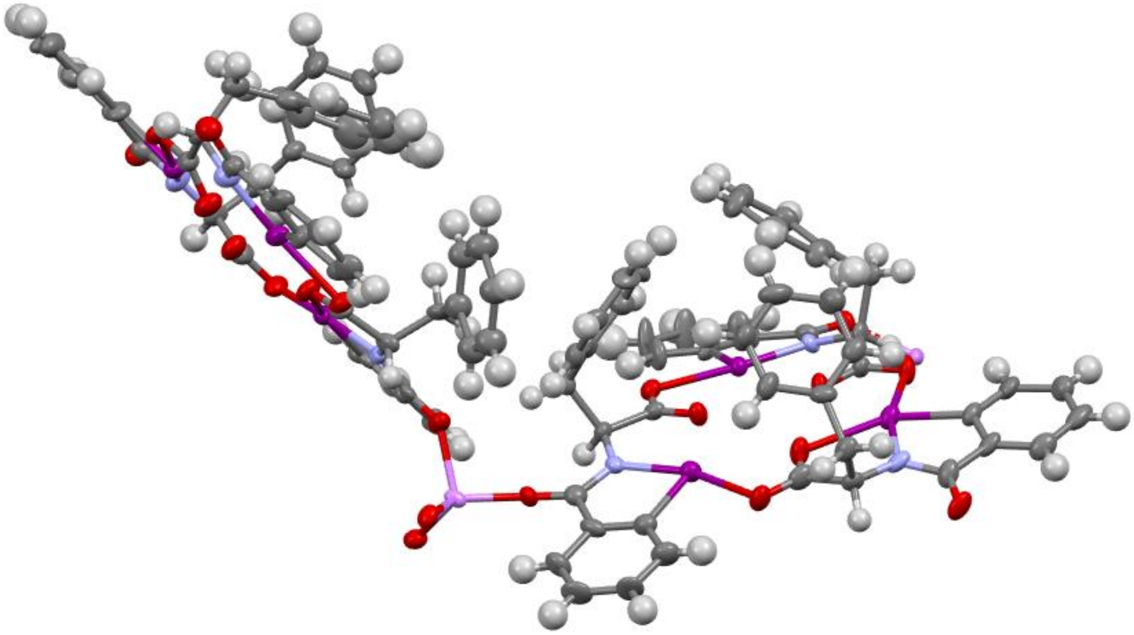

Figure S11. Perspective view of crystallographically independent molecules of phenylalanine HIM **1** /$LiBArF_{20}$ complex. Solvent is removed for clarity. Nitrogen, oxygen, iodine and lithium atoms are denoted by light blue, red, purple and lavender color respectively. Hydrogens atoms are omitted. (Ellipsoid style)

Table 1. Crystal data and structure refinement for 19948ds.

Identification code	2390610	
Empirical formula	C126.80 H91 B F20 I6 Li N6 O20.70	
Formula weight	3189.00	
Temperature	100(2) K	
Wavelength	0.71073 Å	
Crystal system	Orthorhombic	
Space group	$P2_12_12_1$	
Unit cell dimensions	a = 20.2726(7) Å	a= 90°.
	b = 24.0552(9) Å	b= 90°.
	c = 26.2524(11) Å	g = 90°.
Volume	12802.3(8) $Å^3$	
Z	4	
Density (calculated)	1.655 Mg/m^3	
Absorption coefficient	1.554 mm^{-1}	
F(000)	6262	
Crystal size	0.079 x 0.050 x 0.037 mm^3	
Theta range for data collection	2.116 to 23.372°.	
Index ranges	-22<=h<=22, -26<=k<=26, -29<=l<=29	
Reflections collected	198999	
Independent reflections	18490 [R(int) = 0.1898]	
Completeness to theta = 23.372°	99.1 %	
Absorption correction	Semi-empirical from equivalents	
Max. and min. transmission	0.7449 and 0.5705	
Refinement method	Full-matrix least-squares on F^2	
Data / restraints / parameters	18490 / 322 / 1700	
Goodness-of-fit on F^2	1.019	
Final R indices [I>2sigma(I)]	R1 = 0.0501, wR2 = 0.1075	
R indices (all data)	R1 = 0.0710, wR2 = 0.1184	
Absolute structure parameter	-0.017(13)	
Extinction coefficient	n/a	
Largest diff. peak and hole	0.854 and -0.821 e.$Å^{-3}$	

Table 2. Atomic coordinates (x 10^4) and equivalent isotropic displacement parameters ($Å^2$x 10^3) for 19948ds. U(eq) is defined as one third of the trace of the orthogonalized U^{ij} tensor.

	x	y	z	U(eq)
I(1)	6230(1)	1164(1)	6414(1)	37(1)
I(2)	4227(1)	-212(1)	6677(1)	38(1)
I(3)	4310(1)	1104(1)	5080(1)	36(1)
I(4)	8886(1)	4185(1)	2289(1)	33(1)
I(5)	8746(1)	2869(1)	3874(1)	33(1)
I(6)	6937(1)	4444(1)	3620(1)	27(1)
F(1)	6874(4)	-398(3)	3087(4)	42(2)
F(2)	7927(4)	-220(4)	3671(4)	62(3)
F(3)	8191(5)	809(5)	4043(4)	63(3)
F(4)	7325(5)	1650(4)	3855(4)	54(3)
F(5)	6251(4)	1479(3)	3316(3)	42(2)
F(6)	7270(4)	305(4)	2256(4)	42(2)
F(7)	7506(4)	-112(4)	1346(4)	52(3)
F(8)	6516(4)	-556(4)	777(4)	50(3)
F(9)	5267(4)	-549(4)	1138(4)	49(2)
F(10)	5009(4)	-138(3)	2062(3)	36(2)
F(11)	5670(4)	-897(3)	2686(3)	37(2)
F(12)	5035(4)	-1506(3)	3347(4)	45(2)
F(13)	4437(5)	-1040(4)	4177(4)	54(3)
F(14)	4488(5)	80(4)	4296(4)	57(3)
F(15)	5140(4)	719(3)	3647(3)	35(2)
F(16)	6478(4)	1325(4)	2283(4)	42(2)
F(17)	5910(5)	2211(4)	1881(4)	54(3)
F(18)	4572(6)	2328(4)	1906(4)	64(3)
F(19)	3835(5)	1498(4)	2317(4)	57(3)
F(20)	4382(4)	624(4)	2746(3)	40(2)
O(1)	6182(5)	559(5)	7089(4)	43(3)
O(2)	5174(5)	509(5)	6764(4)	47(3)
O(3)	4963(6)	-1018(5)	7961(5)	46(3)
O(4)	3386(5)	-194(5)	6078(4)	36(3)
O(5)	4082(5)	467(5)	5814(4)	44(3)

O(6)	2524(5)	617(5)	4457(4)	39(3)
O(7)	5182(5)	1715(4)	4992(5)	43(3)
O(8)	5343(6)	1353(5)	5758(5)	49(3)
O(9)	7411(5)	2329(5)	5681(5)	48(3)
O(10)	9697(5)	3540(5)	2469(4)	41(3)
O(11)	8980(5)	3439(4)	3093(4)	40(3)
O(12)	10586(5)	2131(4)	3712(4)	42(3)
O(13)	7875(5)	2991(4)	4424(4)	41(3)
O(14)	7749(5)	3671(4)	3868(4)	40(3)
O(15)	6145(5)	4294(4)	5091(4)	32(2)
O(16)	7024(5)	4982(4)	2886(4)	31(2)
O(17)	7835(5)	4360(4)	2801(4)	33(3)
O(18)	8436(5)	5558(4)	1377(4)	37(3)
O(19)	8355(5)	6685(4)	804(4)	34(3)
N(1)	4840(6)	-408(5)	7299(5)	39(3)
N(2)	3435(6)	648(5)	4958(5)	35(3)
N(3)	6575(7)	1726(5)	5856(6)	42(4)
N(4)	9643(6)	2605(5)	3540(5)	34(3)
N(5)	6704(5)	4113(4)	4356(5)	26(3)
N(6)	8363(6)	4864(5)	1973(5)	30(3)
C(1)	5616(8)	318(6)	7043(6)	37(4)
C(2)	5530(7)	-232(7)	7317(7)	41(4)
C(3)	4637(8)	-825(7)	7592(6)	35(4)
C(4)	3979(7)	-1057(7)	7440(6)	37(4)
C(5)	3689(8)	-863(7)	7015(6)	40(4)
C(6)	3091(8)	-1068(7)	6832(7)	42(4)
C(7)	2794(8)	-1477(8)	7112(7)	46(5)
C(8)	3069(9)	-1684(7)	7554(7)	47(5)
C(9)	3656(8)	-1470(7)	7722(7)	40(4)
C(10)	5999(7)	-658(7)	7068(7)	42(4)
C(11)	5776(8)	-823(7)	6554(7)	46(5)
C(12)	5373(8)	-1293(8)	6496(8)	53(5)
C(13)	5117(10)	-1424(9)	6015(8)	62(6)
C(14)	5244(11)	-1074(10)	5603(9)	78(7)
C(15)	5634(10)	-622(9)	5670(8)	69(6)
C(16)	5909(9)	-501(8)	6136(7)	54(5)

C(17)	3582(8)	156(7)	5761(6)	37(4)
C(18)	3213(7)	175(6)	5259(6)	33(4)
C(19)	3071(8)	817(6)	4553(6)	35(4)
C(20)	3416(8)	1217(6)	4213(6)	33(4)
C(21)	4032(8)	1415(6)	4376(6)	34(4)
C(22)	4409(8)	1772(6)	4069(7)	39(4)
C(23)	4143(10)	1904(7)	3594(7)	51(5)
C(24)	3537(8)	1714(7)	3436(6)	40(4)
C(25)	3172(8)	1373(6)	3747(6)	39(4)
C(26)	3326(7)	-372(6)	4981(7)	38(4)
C(27)	3004(14)	-878(10)	5207(16)	46(4)
C(28)	3417(12)	-1309(11)	5354(14)	51(4)
C(29)	3152(14)	-1796(8)	5554(11)	56(5)
C(30)	2472(15)	-1850(8)	5605(10)	57(5)
C(31)	2059(12)	-1418(11)	5457(10)	56(4)
C(32)	2325(13)	-932(10)	5258(14)	51(4)
C(27A)	2970(30)	-860(20)	5210(40)	49(4)
C(28A)	3260(20)	-1350(30)	5390(30)	51(5)
C(29A)	2860(30)	-1771(16)	5570(20)	55(5)
C(30A)	2180(30)	-1710(20)	5590(20)	57(5)
C(31A)	1900(20)	-1220(30)	5410(20)	56(5)
C(32A)	2290(30)	-790(20)	5220(30)	53(5)
C(33)	5532(9)	1639(7)	5397(8)	43(4)
C(34)	6234(8)	1854(6)	5369(7)	43(5)
C(35)	7159(8)	1965(7)	5946(8)	44(5)
C(36)	7482(8)	1752(6)	6418(7)	39(4)
C(37)	7151(8)	1377(6)	6716(7)	37(4)
C(38)	7394(8)	1197(7)	7175(7)	44(4)
C(39)	8021(9)	1408(7)	7306(9)	59(6)
C(40)	8361(9)	1763(7)	7022(9)	58(6)
C(41)	8105(9)	1934(7)	6575(8)	55(5)
C(42)	6600(8)	1651(6)	4902(7)	39(4)
C(43)	6667(7)	1024(6)	4878(7)	36(4)
C(44)	7203(9)	770(8)	5106(8)	57(5)
C(45)	7286(10)	195(8)	5056(8)	62(6)
C(46)	6863(9)	-117(7)	4789(7)	51(5)

C(47)	6320(9)	143(7)	4554(6)	46(4)
C(48)	6238(8)	703(6)	4597(6)	40(4)
C(49)	9492(8)	3302(7)	2867(7)	42(4)
C(50)	9878(7)	2801(6)	3047(6)	33(4)
C(51)	10007(8)	2261(7)	3822(6)	39(4)
C(52)	9658(7)	2044(7)	4275(6)	35(4)
C(53)	9046(7)	2261(6)	4384(6)	33(4)
C(54)	8702(8)	2097(7)	4824(6)	41(4)
C(55)	8953(8)	1675(7)	5126(7)	47(5)
C(56)	9567(8)	1435(7)	4989(7)	46(5)
C(57)	9933(9)	1625(7)	4571(7)	45(5)
C(58)	9817(7)	2342(7)	2634(6)	37(4)
C(59)	9107(7)	2258(7)	2468(6)	39(4)
C(60)	8653(8)	1935(6)	2743(7)	40(4)
C(61)	8010(9)	1877(8)	2593(7)	56(5)
C(62)	7787(8)	2166(8)	2155(8)	56(5)
C(63)	8230(8)	2491(8)	1879(7)	51(5)
C(64)	8875(9)	2535(7)	2033(7)	45(4)
C(65)	7565(7)	3416(6)	4241(6)	31(4)
C(66)	6924(7)	3573(6)	4522(6)	33(4)
C(67)	6293(7)	4412(6)	4646(6)	31(4)
C(68)	6043(8)	4912(6)	4379(6)	34(4)
C(69)	6243(7)	5013(6)	3862(6)	35(4)
C(70)	6030(9)	5472(7)	3608(6)	47(5)
C(71)	5563(11)	5819(8)	3857(8)	75(7)
C(72)	5369(10)	5739(8)	4344(7)	67(6)
C(73)	5590(8)	5274(7)	4620(6)	42(4)
C(74)	6402(7)	3116(7)	4446(6)	34(4)
C(75)	6146(8)	3085(6)	3912(6)	32(4)
C(76)	5551(8)	3353(7)	3776(7)	40(4)
C(77)	5323(9)	3355(7)	3288(7)	46(5)
C(78)	5670(9)	3090(7)	2911(7)	47(5)
C(79)	6249(10)	2815(7)	3034(6)	52(5)
C(80)	6489(8)	2813(6)	3523(6)	39(4)
C(81)	7520(7)	4781(6)	2645(6)	29(4)
C(82)	7712(7)	5058(6)	2145(6)	31(4)

C(83)	8678(7)	5138(6)	1597(6)	34(4)
C(84)	9335(8)	4908(6)	1469(6)	37(4)
C(85)	9560(7)	4460(7)	1733(6)	38(4)
C(86)	10174(8)	4219(8)	1643(7)	51(5)
C(87)	10565(7)	4467(8)	1271(7)	51(5)
C(88)	10349(8)	4925(8)	995(7)	50(5)
C(89)	9751(8)	5164(7)	1103(7)	47(5)
C(90)	7170(7)	4952(6)	1746(6)	31(4)
C(91)	7102(7)	4347(6)	1590(6)	33(4)
C(92)	6680(8)	3984(7)	1860(7)	44(4)
C(93)	6613(9)	3443(7)	1695(8)	52(5)
C(94)	6948(9)	3239(6)	1283(8)	47(5)
C(95)	7366(7)	3579(7)	1025(7)	40(4)
C(96)	7447(8)	4140(7)	1173(6)	39(4)
C(97)	6513(7)	540(7)	3147(6)	37(4)
C(98)	6958(8)	129(7)	3273(7)	40(4)
C(99)	7509(8)	208(7)	3567(7)	47(4)
C(100)	7654(8)	716(8)	3759(7)	46(5)
C(101)	7208(8)	1140(7)	3669(7)	41(4)
C(102)	6665(7)	1044(6)	3374(6)	31(4)
C(103)	6107(7)	97(6)	2224(6)	32(4)
C(104)	6747(7)	87(6)	1996(7)	34(4)
C(105)	6893(8)	-121(7)	1525(7)	40(4)
C(106)	6400(8)	-342(6)	1250(6)	38(4)
C(107)	5768(8)	-343(6)	1423(7)	39(4)
C(108)	5645(7)	-125(6)	1906(6)	32(4)
C(109)	5436(7)	-47(6)	3120(6)	27(3)
C(110)	5382(6)	-617(6)	3079(6)	26(3)
C(111)	5058(7)	-952(6)	3415(6)	32(4)
C(112)	4753(7)	-721(6)	3829(7)	38(4)
C(113)	4776(8)	-155(7)	3886(6)	37(4)
C(114)	5118(7)	155(6)	3542(7)	37(4)
C(115)	5480(7)	936(6)	2565(6)	31(4)
C(116)	5813(7)	1353(6)	2325(6)	32(4)
C(117)	5541(8)	1814(6)	2093(7)	41(4)
C(118)	4854(9)	1874(6)	2122(7)	44(5)

C(119)	4497(8)	1478(7)	2323(7)	39(4)
C(120)	4797(7)	1003(6)	2555(6)	34(4)
B(1)	5882(9)	386(7)	2772(7)	35(4)
Li(1)	8350(11)	5890(9)	716(9)	24(5)
C(121)	10417(12)	2628(8)	-648(7)	83(9)
C(122)	10315(9)	2613(6)	-75(6)	78(6)
O(20)	9846(6)	3026(4)	60(4)	64(4)
C(123)	9713(9)	3049(7)	591(4)	63(6)
C(124)	9208(9)	3503(7)	689(6)	39(5)
C(125)	8083(13)	1955(14)	519(13)	79(10)
C(126)	7545(13)	1588(14)	741(8)	79(8)
O(21)	7140(10)	1392(9)	339(7)	84(7)
C(127)	6618(13)	1042(13)	506(10)	87(8)
C(128)	6219(14)	859(15)	45(13)	96(11)
C(129)	1467(12)	6752(13)	6013(16)	99(11)
C(130)	2184(12)	6567(10)	5996(12)	94(9)
O(22)	2550(10)	6886(7)	6353(9)	89(7)
C(131)	3230(11)	6747(10)	6369(12)	82(8)
C(132)	3569(13)	7113(11)	6762(13)	76(10)

Table 3. Bond lengths [Å] and angles [°] for 19948ds.

I(1)-C(37)	2.091(16)
I(1)-N(3)	2.112(14)
I(1)-O(1)	2.294(11)
I(2)-C(5)	2.104(17)
I(2)-N(1)	2.106(13)
I(2)-O(4)	2.321(10)
I(3)-C(21)	2.071(16)
I(3)-N(2)	2.109(12)
I(3)-O(7)	2.310(11)
I(4)-C(85)	2.106(16)
I(4)-N(6)	2.117(12)
I(4)-O(10)	2.309(10)
I(5)-C(53)	2.073(15)
I(5)-N(4)	2.117(12)
I(5)-O(13)	2.300(11)
I(6)-C(69)	2.062(15)
I(6)-N(5)	2.142(12)
I(6)-O(16)	2.328(10)
F(1)-C(98)	1.371(18)
F(2)-C(99)	1.362(19)
F(3)-C(100)	1.338(18)
F(4)-C(101)	1.342(18)
F(5)-C(102)	1.349(16)
F(6)-C(104)	1.367(17)
F(7)-C(105)	1.330(17)
F(8)-C(106)	1.363(17)
F(9)-C(107)	1.355(18)
F(10)-C(108)	1.352(16)
F(11)-C(110)	1.365(15)
F(12)-C(111)	1.346(16)
F(13)-C(112)	1.354(17)
F(14)-C(113)	1.347(18)
F(15)-C(114)	1.385(17)
F(16)-C(116)	1.355(17)

F(17)-C(117)	1.336(18)
F(18)-C(118)	1.357(18)
F(19)-C(119)	1.344(18)
F(20)-C(120)	1.338(17)
O(1)-C(1)	1.293(18)
O(2)-C(1)	1.246(18)
O(3)-C(3)	1.260(18)
O(4)-C(17)	1.249(19)
O(5)-C(17)	1.269(19)
O(6)-C(19)	1.236(18)
O(6)-Li(1)#1	1.94(2)
O(7)-C(33)	1.29(2)
O(8)-C(33)	1.23(2)
O(9)-C(35)	1.23(2)
O(10)-C(49)	1.26(2)
O(11)-C(49)	1.241(19)
O(12)-C(51)	1.249(18)
O(13)-C(65)	1.295(17)
O(14)-C(65)	1.213(18)
O(15)-C(67)	1.239(17)
O(15)-Li(1)#2	1.98(2)
O(16)-C(81)	1.282(17)
O(17)-C(81)	1.265(17)
O(18)-C(83)	1.261(17)
O(18)-Li(1)	1.92(3)
O(19)-Li(1)	1.92(2)
O(19)-H(19A)	0.9584
O(19)-H(19B)	0.9585
N(1)-C(3)	1.33(2)
N(1)-C(2)	1.461(19)
N(2)-C(19)	1.356(19)
N(2)-C(18)	1.458(19)
N(3)-C(35)	1.34(2)
N(3)-C(34)	1.49(2)
N(4)-C(51)	1.332(19)
N(4)-C(50)	1.46(2)

N(5)-C(67)	1.340(18)
N(5)-C(66)	1.440(17)
N(6)-C(83)	1.347(19)
N(6)-C(82)	1.471(18)
C(1)-C(2)	1.51(2)
C(2)-C(10)	1.54(2)
C(2)-H(2)	1.0000
C(3)-C(4)	1.50(2)
C(4)-C(5)	1.35(2)
C(4)-C(9)	1.40(2)
C(5)-C(6)	1.39(2)
C(6)-C(7)	1.37(2)
C(6)-H(6)	0.9500
C(7)-C(8)	1.38(2)
C(7)-H(7)	0.9500
C(8)-C(9)	1.37(2)
C(8)-H(8)	0.9500
C(9)-H(9)	0.9500
C(10)-C(11)	1.48(2)
C(10)-H(10A)	0.9900
C(10)-H(10B)	0.9900
C(11)-C(16)	1.37(2)
C(11)-C(12)	1.40(2)
C(12)-C(13)	1.40(3)
C(12)-H(12)	0.9500
C(13)-C(14)	1.39(3)
C(13)-H(13)	0.9500
C(14)-C(15)	1.36(3)
C(14)-H(14)	0.9500
C(15)-C(16)	1.37(3)
C(15)-H(15)	0.9500
C(16)-H(16)	0.9500
C(17)-C(18)	1.52(2)
C(18)-C(26)	1.52(2)
C(18)-H(18)	1.0000
C(19)-C(20)	1.49(2)

C(20)-C(25)	1.37(2)
C(20)-C(21)	1.40(2)
C(21)-C(22)	1.40(2)
C(22)-C(23)	1.39(2)
C(22)-H(22)	0.9500
C(23)-C(24)	1.37(2)
C(23)-H(23)	0.9500
C(24)-C(25)	1.37(2)
C(24)-H(24)	0.9500
C(25)-H(25)	0.9500
C(26)-C(27A)	1.50(2)
C(26)-C(27)	1.504(18)
C(26)-H(26A)	0.9900
C(26)-H(26B)	0.9900
C(27)-C(28)	1.3900
C(27)-C(32)	1.3900
C(28)-C(29)	1.3900
C(28)-H(28)	0.9500
C(29)-C(30)	1.3900
C(29)-H(29)	0.9500
C(30)-C(31)	1.3900
C(30)-H(30)	0.9500
C(31)-C(32)	1.3900
C(31)-H(31)	0.9500
C(32)-H(32)	0.9500
C(27A)-C(28A)	1.3900
C(27A)-C(32A)	1.3900
C(28A)-C(29A)	1.3900
C(28A)-H(28A)	0.9500
C(29A)-C(30A)	1.3900
C(29A)-H(29A)	0.9500
C(30A)-C(31A)	1.3900
C(30A)-H(30A)	0.9500
C(31A)-C(32A)	1.3900
C(31A)-H(31A)	0.9500
C(32A)-H(32A)	0.9500

C(33)-C(34)	1.51(2)
C(34)-C(42)	1.51(2)
C(34)-H(34)	1.0000
C(35)-C(36)	1.49(2)
C(36)-C(37)	1.37(2)
C(36)-C(41)	1.40(2)
C(37)-C(38)	1.37(2)
C(38)-C(39)	1.41(2)
C(38)-H(38)	0.9500
C(39)-C(40)	1.33(3)
C(39)-H(39)	0.9500
C(40)-C(41)	1.35(3)
C(40)-H(40)	0.9500
C(41)-H(41)	0.9500
C(42)-C(43)	1.52(2)
C(42)-H(42A)	0.9900
C(42)-H(42B)	0.9900
C(43)-C(48)	1.38(2)
C(43)-C(44)	1.38(2)
C(44)-C(45)	1.40(3)
C(44)-H(44)	0.9500
C(45)-C(46)	1.34(2)
C(45)-H(45)	0.9500
C(46)-C(47)	1.41(2)
C(46)-H(46)	0.9500
C(47)-C(48)	1.36(2)
C(47)-H(47)	0.9500
C(48)-H(48)	0.9500
C(49)-C(50)	1.51(2)
C(50)-C(58)	1.55(2)
C(50)-H(50)	1.0000
C(51)-C(52)	1.48(2)
C(52)-C(53)	1.38(2)
C(52)-C(57)	1.39(2)
C(53)-C(54)	1.41(2)
C(54)-C(55)	1.39(2)

C(54)-H(54)	0.9500
C(55)-C(56)	1.42(2)
C(55)-H(55)	0.9500
C(56)-C(57)	1.40(2)
C(56)-H(56)	0.9500
C(57)-H(57)	0.9500
C(58)-C(59)	1.52(2)
C(58)-H(58A)	0.9900
C(58)-H(58B)	0.9900
C(59)-C(60)	1.40(2)
C(59)-C(64)	1.40(2)
C(60)-C(61)	1.37(2)
C(60)-H(60)	0.9500
C(61)-C(62)	1.42(3)
C(61)-H(61)	0.9500
C(62)-C(63)	1.39(2)
C(62)-H(62)	0.9500
C(63)-C(64)	1.37(2)
C(63)-H(63)	0.9500
C(64)-H(64)	0.9500
C(65)-C(66)	1.54(2)
C(66)-C(74)	1.54(2)
C(66)-H(66)	1.0000
C(67)-C(68)	1.48(2)
C(68)-C(73)	1.41(2)
C(68)-C(69)	1.44(2)
C(69)-C(70)	1.36(2)
C(70)-C(71)	1.42(2)
C(70)-H(70)	0.9500
C(71)-C(72)	1.35(3)
C(71)-H(71)	0.9500
C(72)-C(73)	1.41(2)
C(72)-H(72)	0.9500
C(73)-H(73)	0.9500
C(74)-C(75)	1.50(2)
C(74)-H(74A)	0.9900

C(74)-H(74B)	0.9900
C(75)-C(80)	1.40(2)
C(75)-C(76)	1.41(2)
C(76)-C(77)	1.36(2)
C(76)-H(76)	0.9500
C(77)-C(78)	1.37(2)
C(77)-H(77)	0.9500
C(78)-C(79)	1.39(2)
C(78)-H(78)	0.9500
C(79)-C(80)	1.37(2)
C(79)-H(79)	0.9500
C(80)-H(80)	0.9500
C(81)-C(82)	1.52(2)
C(82)-C(90)	1.54(2)
C(82)-H(82)	1.0000
C(83)-C(84)	1.48(2)
C(84)-C(85)	1.36(2)
C(84)-C(89)	1.42(2)
C(85)-C(86)	1.39(2)
C(86)-C(87)	1.39(2)
C(86)-H(86)	0.9500
C(87)-C(88)	1.39(2)
C(87)-H(87)	0.9500
C(88)-C(89)	1.37(2)
C(88)-H(88)	0.9500
C(89)-H(89)	0.9500
C(90)-C(91)	1.52(2)
C(90)-H(90A)	0.9900
C(90)-H(90B)	0.9900
C(91)-C(96)	1.39(2)
C(91)-C(92)	1.41(2)
C(92)-C(93)	1.38(2)
C(92)-H(92)	0.9500
C(93)-C(94)	1.37(3)
C(93)-H(93)	0.9500
C(94)-C(95)	1.36(2)

C(94)-H(94)	0.9500
C(95)-C(96)	1.41(2)
C(95)-H(95)	0.9500
C(96)-H(96)	0.9500
C(97)-C(98)	1.38(2)
C(97)-C(102)	1.38(2)
C(97)-B(1)	1.66(2)
C(98)-C(99)	1.37(2)
C(99)-C(100)	1.35(3)
C(100)-C(101)	1.38(2)
C(101)-C(102)	1.36(2)
C(103)-C(108)	1.36(2)
C(103)-C(104)	1.43(2)
C(103)-B(1)	1.66(2)
C(104)-C(105)	1.37(2)
C(105)-C(106)	1.34(2)
C(106)-C(107)	1.36(2)
C(107)-C(108)	1.40(2)
C(109)-C(114)	1.37(2)
C(109)-C(110)	1.38(2)
C(109)-B(1)	1.65(2)
C(110)-C(111)	1.36(2)
C(111)-C(112)	1.37(2)
C(112)-C(113)	1.37(2)
C(113)-C(114)	1.36(2)
C(115)-C(116)	1.36(2)
C(115)-C(120)	1.39(2)
C(115)-B(1)	1.65(2)
C(116)-C(117)	1.38(2)
C(117)-C(118)	1.40(2)
C(118)-C(119)	1.31(2)
C(119)-C(120)	1.43(2)
C(121)-C(122)	1.5197
C(121)-H(12A)	0.9800
C(121)-H(12B)	0.9800
C(121)-H(12C)	0.9800

C(122)-O(20)	1.4201
C(122)-H(12D)	0.9900
C(122)-H(12E)	0.9900
O(20)-C(123)	1.4202
C(123)-C(124)	1.5197
C(123)-H(12F)	0.9900
C(123)-H(12G)	0.9900
C(124)-H(12H)	0.9800
C(124)-H(12I)	0.9800
C(124)-H(12J)	0.9800
C(125)-C(126)	1.5199
C(125)-H(12K)	0.9800
C(125)-H(12L)	0.9800
C(125)-H(12M)	0.9800
C(126)-O(21)	1.4201
C(126)-H(12N)	0.9900
C(126)-H(12O)	0.9900
O(21)-C(127)	1.4201
C(127)-C(128)	1.5200
C(127)-H(12P)	0.9900
C(127)-H(12Q)	0.9900
C(128)-H(12R)	0.9800
C(128)-H(12S)	0.9800
C(128)-H(12T)	0.9800
C(129)-C(130)	1.5201
C(129)-H(12U)	0.9800
C(129)-H(12V)	0.9800
C(129)-H(12W)	0.9800
C(130)-O(22)	1.4201
C(130)-H(13A)	0.9900
C(130)-H(13B)	0.9900
O(22)-C(131)	1.4203
C(131)-C(132)	1.5198
C(131)-H(13C)	0.9900
C(131)-H(13D)	0.9900
C(132)-H(13E)	0.9800

C(132)-H(13F)	0.9800
C(132)-H(13G)	0.9800

C(37)-I(1)-N(3)	79.1(6)
C(37)-I(1)-O(1)	84.3(5)
N(3)-I(1)-O(1)	162.8(4)
C(5)-I(2)-N(1)	79.2(5)
C(5)-I(2)-O(4)	85.3(5)
N(1)-I(2)-O(4)	164.5(4)
C(21)-I(3)-N(2)	79.9(6)
C(21)-I(3)-O(7)	83.6(5)
N(2)-I(3)-O(7)	163.5(5)
C(85)-I(4)-N(6)	79.1(6)
C(85)-I(4)-O(10)	83.7(5)
N(6)-I(4)-O(10)	162.7(4)
C(53)-I(5)-N(4)	78.7(6)
C(53)-I(5)-O(13)	84.8(5)
N(4)-I(5)-O(13)	163.3(4)
C(69)-I(6)-N(5)	79.6(6)
C(69)-I(6)-O(16)	86.4(5)
N(5)-I(6)-O(16)	165.9(4)
C(1)-O(1)-I(1)	104.5(9)
C(17)-O(4)-I(2)	103.3(9)
C(19)-O(6)-Li(1)#1	137.3(12)
C(33)-O(7)-I(3)	104.4(10)
C(49)-O(10)-I(4)	103.9(10)
C(65)-O(13)-I(5)	103.9(9)
C(67)-O(15)-Li(1)#2	135.0(12)
C(81)-O(16)-I(6)	105.0(9)
C(83)-O(18)-Li(1)	141.4(13)
Li(1)-O(19)-H(19A)	144.9
Li(1)-O(19)-H(19B)	110.5
H(19A)-O(19)-H(19B)	104.5
C(3)-N(1)-C(2)	119.8(14)
C(3)-N(1)-I(2)	115.7(10)
C(2)-N(1)-I(2)	121.6(11)

C(19)-N(2)-C(18)	119.4(13)
C(19)-N(2)-I(3)	114.9(11)
C(18)-N(2)-I(3)	125.7(10)
C(35)-N(3)-C(34)	118.4(14)
C(35)-N(3)-I(1)	116.5(12)
C(34)-N(3)-I(1)	125.1(10)
C(51)-N(4)-C(50)	120.9(13)
C(51)-N(4)-I(5)	115.5(11)
C(50)-N(4)-I(5)	123.5(9)
C(67)-N(5)-C(66)	120.3(13)
C(67)-N(5)-I(6)	116.7(9)
C(66)-N(5)-I(6)	122.7(10)
C(83)-N(6)-C(82)	119.7(12)
C(83)-N(6)-I(4)	115.4(10)
C(82)-N(6)-I(4)	124.9(9)
O(2)-C(1)-O(1)	121.8(15)
O(2)-C(1)-C(2)	121.3(14)
O(1)-C(1)-C(2)	116.7(13)
N(1)-C(2)-C(1)	110.3(12)
N(1)-C(2)-C(10)	112.6(13)
C(1)-C(2)-C(10)	107.9(14)
N(1)-C(2)-H(2)	108.7
C(1)-C(2)-H(2)	108.7
C(10)-C(2)-H(2)	108.7
O(3)-C(3)-N(1)	124.0(15)
O(3)-C(3)-C(4)	122.3(15)
N(1)-C(3)-C(4)	113.7(14)
C(5)-C(4)-C(9)	118.6(15)
C(5)-C(4)-C(3)	118.7(15)
C(9)-C(4)-C(3)	122.6(16)
C(4)-C(5)-C(6)	122.9(16)
C(4)-C(5)-I(2)	112.4(12)
C(6)-C(5)-I(2)	124.7(12)
C(7)-C(6)-C(5)	116.9(17)
C(7)-C(6)-H(6)	121.5
C(5)-C(6)-H(6)	121.5

C(6)-C(7)-C(8)	122.3(17)
C(6)-C(7)-H(7)	118.8
C(8)-C(7)-H(7)	118.8
C(9)-C(8)-C(7)	118.9(17)
C(9)-C(8)-H(8)	120.6
C(7)-C(8)-H(8)	120.6
C(8)-C(9)-C(4)	120.3(17)
C(8)-C(9)-H(9)	119.8
C(4)-C(9)-H(9)	119.8
C(11)-C(10)-C(2)	112.1(13)
C(11)-C(10)-H(10A)	109.2
C(2)-C(10)-H(10A)	109.2
C(11)-C(10)-H(10B)	109.2
C(2)-C(10)-H(10B)	109.2
H(10A)-C(10)-H(10B)	107.9
C(16)-C(11)-C(12)	119.0(18)
C(16)-C(11)-C(10)	121.3(16)
C(12)-C(11)-C(10)	119.5(16)
C(13)-C(12)-C(11)	119.6(18)
C(13)-C(12)-H(12)	120.2
C(11)-C(12)-H(12)	120.2
C(14)-C(13)-C(12)	120(2)
C(14)-C(13)-H(13)	120.2
C(12)-C(13)-H(13)	120.2
C(15)-C(14)-C(13)	119(2)
C(15)-C(14)-H(14)	120.3
C(13)-C(14)-H(14)	120.3
C(14)-C(15)-C(16)	121(2)
C(14)-C(15)-H(15)	119.3
C(16)-C(15)-H(15)	119.3
C(11)-C(16)-C(15)	120.9(18)
C(11)-C(16)-H(16)	119.5
C(15)-C(16)-H(16)	119.5
O(4)-C(17)-O(5)	125.4(14)
O(4)-C(17)-C(18)	116.3(15)
O(5)-C(17)-C(18)	118.2(15)

N(2)-C(18)-C(17)	110.1(13)
N(2)-C(18)-C(26)	111.5(12)
C(17)-C(18)-C(26)	108.5(13)
N(2)-C(18)-H(18)	108.9
C(17)-C(18)-H(18)	108.9
C(26)-C(18)-H(18)	108.9
O(6)-C(19)-N(2)	122.2(15)
O(6)-C(19)-C(20)	123.4(15)
N(2)-C(19)-C(20)	114.2(14)
C(25)-C(20)-C(21)	120.2(16)
C(25)-C(20)-C(19)	122.9(14)
C(21)-C(20)-C(19)	116.9(15)
C(22)-C(21)-C(20)	120.9(16)
C(22)-C(21)-I(3)	125.9(12)
C(20)-C(21)-I(3)	113.1(12)
C(23)-C(22)-C(21)	116.3(16)
C(23)-C(22)-H(22)	121.8
C(21)-C(22)-H(22)	121.8
C(24)-C(23)-C(22)	122.6(18)
C(24)-C(23)-H(23)	118.7
C(22)-C(23)-H(23)	118.7
C(23)-C(24)-C(25)	120.1(17)
C(23)-C(24)-H(24)	120.0
C(25)-C(24)-H(24)	120.0
C(20)-C(25)-C(24)	119.8(16)
C(20)-C(25)-H(25)	120.1
C(24)-C(25)-H(25)	120.1
C(27A)-C(26)-C(18)	114(4)
C(27)-C(26)-C(18)	116(2)
C(27)-C(26)-H(26A)	108.2
C(18)-C(26)-H(26A)	108.2
C(27)-C(26)-H(26B)	108.2
C(18)-C(26)-H(26B)	108.2
H(26A)-C(26)-H(26B)	107.4
C(28)-C(27)-C(32)	120.0
C(28)-C(27)-C(26)	116.9(18)

C(32)-C(27)-C(26)	123.0(18)
C(27)-C(28)-C(29)	120.0
C(27)-C(28)-H(28)	120.0
C(29)-C(28)-H(28)	120.0
C(30)-C(29)-C(28)	120.0
C(30)-C(29)-H(29)	120.0
C(28)-C(29)-H(29)	120.0
C(29)-C(30)-C(31)	120.0
C(29)-C(30)-H(30)	120.0
C(31)-C(30)-H(30)	120.0
C(32)-C(31)-C(30)	120.0
C(32)-C(31)-H(31)	120.0
C(30)-C(31)-H(31)	120.0
C(31)-C(32)-C(27)	120.0
C(31)-C(32)-H(32)	120.0
C(27)-C(32)-H(32)	120.0
C(28A)-C(27A)-C(32A)	120.0
C(28A)-C(27A)-C(26)	127(5)
C(32A)-C(27A)-C(26)	113(5)
C(29A)-C(28A)-C(27A)	120.0
C(29A)-C(28A)-H(28A)	120.0
C(27A)-C(28A)-H(28A)	120.0
C(28A)-C(29A)-C(30A)	120.0
C(28A)-C(29A)-H(29A)	120.0
C(30A)-C(29A)-H(29A)	120.0
C(29A)-C(30A)-C(31A)	120.0
C(29A)-C(30A)-H(30A)	120.0
C(31A)-C(30A)-H(30A)	120.0
C(32A)-C(31A)-C(30A)	120.0
C(32A)-C(31A)-H(31A)	120.0
C(30A)-C(31A)-H(31A)	120.0
C(31A)-C(32A)-C(27A)	120.0
C(31A)-C(32A)-H(32A)	120.0
C(27A)-C(32A)-H(32A)	120.0
O(8)-C(33)-O(7)	122.7(16)
O(8)-C(33)-C(34)	121.3(16)

O(7)-C(33)-C(34)	115.4(16)
N(3)-C(34)-C(42)	113.7(13)
N(3)-C(34)-C(33)	109.0(15)
C(42)-C(34)-C(33)	113.0(14)
N(3)-C(34)-H(34)	106.9
C(42)-C(34)-H(34)	106.9
C(33)-C(34)-H(34)	106.9
O(9)-C(35)-N(3)	125.0(17)
O(9)-C(35)-C(36)	122.2(15)
N(3)-C(35)-C(36)	112.8(15)
C(37)-C(36)-C(41)	118.8(17)
C(37)-C(36)-C(35)	119.0(14)
C(41)-C(36)-C(35)	122.2(16)
C(36)-C(37)-C(38)	122.2(16)
C(36)-C(37)-I(1)	112.5(11)
C(38)-C(37)-I(1)	125.2(13)
C(37)-C(38)-C(39)	115.2(18)
C(37)-C(38)-H(38)	122.4
C(39)-C(38)-H(38)	122.4
C(40)-C(39)-C(38)	124(2)
C(40)-C(39)-H(39)	117.9
C(38)-C(39)-H(39)	117.9
C(39)-C(40)-C(41)	119.1(18)
C(39)-C(40)-H(40)	120.4
C(41)-C(40)-H(40)	120.4
C(40)-C(41)-C(36)	120.5(18)
C(40)-C(41)-H(41)	119.8
C(36)-C(41)-H(41)	119.8
C(34)-C(42)-C(43)	113.4(14)
C(34)-C(42)-H(42A)	108.9
C(43)-C(42)-H(42A)	108.9
C(34)-C(42)-H(42B)	108.9
C(43)-C(42)-H(42B)	108.9
H(42A)-C(42)-H(42B)	107.7
C(48)-C(43)-C(44)	118.7(15)
C(48)-C(43)-C(42)	121.5(14)

C(44)-C(43)-C(42)	119.5(15)
C(43)-C(44)-C(45)	119.3(18)
C(43)-C(44)-H(44)	120.3
C(45)-C(44)-H(44)	120.3
C(46)-C(45)-C(44)	121.8(18)
C(46)-C(45)-H(45)	119.1
C(44)-C(45)-H(45)	119.1
C(45)-C(46)-C(47)	118.8(17)
C(45)-C(46)-H(46)	120.6
C(47)-C(46)-H(46)	120.6
C(48)-C(47)-C(46)	119.9(17)
C(48)-C(47)-H(47)	120.0
C(46)-C(47)-H(47)	120.0
C(47)-C(48)-C(43)	121.4(16)
C(47)-C(48)-H(48)	119.3
C(43)-C(48)-H(48)	119.3
O(11)-C(49)-O(10)	123.5(15)
O(11)-C(49)-C(50)	119.6(16)
O(10)-C(49)-C(50)	116.8(14)
N(4)-C(50)-C(49)	111.6(13)
N(4)-C(50)-C(58)	111.4(12)
C(49)-C(50)-C(58)	107.8(13)
N(4)-C(50)-H(50)	108.7
C(49)-C(50)-H(50)	108.7
C(58)-C(50)-H(50)	108.7
O(12)-C(51)-N(4)	123.2(16)
O(12)-C(51)-C(52)	123.2(14)
N(4)-C(51)-C(52)	113.7(14)
C(53)-C(52)-C(57)	121.4(16)
C(53)-C(52)-C(51)	117.6(14)
C(57)-C(52)-C(51)	120.9(14)
C(52)-C(53)-C(54)	120.8(15)
C(52)-C(53)-I(5)	113.5(12)
C(54)-C(53)-I(5)	125.6(11)
C(55)-C(54)-C(53)	119.6(16)
C(55)-C(54)-H(54)	120.2

C(53)-C(54)-H(54)	120.2
C(54)-C(55)-C(56)	118.4(17)
C(54)-C(55)-H(55)	120.8
C(56)-C(55)-H(55)	120.8
C(57)-C(56)-C(55)	122.0(17)
C(57)-C(56)-H(56)	119.0
C(55)-C(56)-H(56)	119.0
C(52)-C(57)-C(56)	117.6(16)
C(52)-C(57)-H(57)	121.2
C(56)-C(57)-H(57)	121.2
C(59)-C(58)-C(50)	111.8(12)
C(59)-C(58)-H(58A)	109.3
C(50)-C(58)-H(58A)	109.3
C(59)-C(58)-H(58B)	109.3
C(50)-C(58)-H(58B)	109.3
H(58A)-C(58)-H(58B)	107.9
C(60)-C(59)-C(64)	117.6(15)
C(60)-C(59)-C(58)	123.2(15)
C(64)-C(59)-C(58)	119.2(15)
C(61)-C(60)-C(59)	122.2(17)
C(61)-C(60)-H(60)	118.9
C(59)-C(60)-H(60)	118.9
C(60)-C(61)-C(62)	119.1(17)
C(60)-C(61)-H(61)	120.5
C(62)-C(61)-H(61)	120.5
C(63)-C(62)-C(61)	119.5(16)
C(63)-C(62)-H(62)	120.3
C(61)-C(62)-H(62)	120.3
C(64)-C(63)-C(62)	120.3(18)
C(64)-C(63)-H(63)	119.9
C(62)-C(63)-H(63)	119.9
C(63)-C(64)-C(59)	121.4(17)
C(63)-C(64)-H(64)	119.3
C(59)-C(64)-H(64)	119.3
O(14)-C(65)-O(13)	123.4(14)
O(14)-C(65)-C(66)	121.5(13)

O(13)-C(65)-C(66)	115.2(13)
N(5)-C(66)-C(74)	113.1(12)
N(5)-C(66)-C(65)	109.7(12)
C(74)-C(66)-C(65)	110.0(12)
N(5)-C(66)-H(66)	108.0
C(74)-C(66)-H(66)	108.0
C(65)-C(66)-H(66)	108.0
O(15)-C(67)-N(5)	124.3(14)
O(15)-C(67)-C(68)	123.4(14)
N(5)-C(67)-C(68)	112.3(13)
C(73)-C(68)-C(69)	120.2(13)
C(73)-C(68)-C(67)	120.5(14)
C(69)-C(68)-C(67)	119.2(14)
C(70)-C(69)-C(68)	120.6(15)
C(70)-C(69)-I(6)	127.2(13)
C(68)-C(69)-I(6)	111.8(10)
C(69)-C(70)-C(71)	117.6(16)
C(69)-C(70)-H(70)	121.2
C(71)-C(70)-H(70)	121.2
C(72)-C(71)-C(70)	123.1(16)
C(72)-C(71)-H(71)	118.5
C(70)-C(71)-H(71)	118.5
C(71)-C(72)-C(73)	120.5(16)
C(71)-C(72)-H(72)	119.8
C(73)-C(72)-H(72)	119.8
C(72)-C(73)-C(68)	117.7(15)
C(72)-C(73)-H(73)	121.1
C(68)-C(73)-H(73)	121.1
C(75)-C(74)-C(66)	113.2(13)
C(75)-C(74)-H(74A)	108.9
C(66)-C(74)-H(74A)	108.9
C(75)-C(74)-H(74B)	108.9
C(66)-C(74)-H(74B)	108.9
H(74A)-C(74)-H(74B)	107.8
C(80)-C(75)-C(76)	116.9(15)
C(80)-C(75)-C(74)	122.4(14)

C(76)-C(75)-C(74)	120.7(14)
C(77)-C(76)-C(75)	122.0(16)
C(77)-C(76)-H(76)	119.0
C(75)-C(76)-H(76)	119.0
C(76)-C(77)-C(78)	120.1(17)
C(76)-C(77)-H(77)	119.9
C(78)-C(77)-H(77)	119.9
C(77)-C(78)-C(79)	119.2(16)
C(77)-C(78)-H(78)	120.4
C(79)-C(78)-H(78)	120.4
C(80)-C(79)-C(78)	121.4(16)
C(80)-C(79)-H(79)	119.3
C(78)-C(79)-H(79)	119.3
C(79)-C(80)-C(75)	120.4(16)
C(79)-C(80)-H(80)	119.8
C(75)-C(80)-H(80)	119.8
O(17)-C(81)-O(16)	122.5(14)
O(17)-C(81)-C(82)	120.0(13)
O(16)-C(81)-C(82)	117.5(13)
N(6)-C(82)-C(81)	110.9(12)
N(6)-C(82)-C(90)	112.3(13)
C(81)-C(82)-C(90)	109.3(11)
N(6)-C(82)-H(82)	108.1
C(81)-C(82)-H(82)	108.1
C(90)-C(82)-H(82)	108.1
O(18)-C(83)-N(6)	122.8(14)
O(18)-C(83)-C(84)	123.1(14)
N(6)-C(83)-C(84)	114.1(13)
C(85)-C(84)-C(89)	119.2(15)
C(85)-C(84)-C(83)	118.9(14)
C(89)-C(84)-C(83)	121.8(15)
C(84)-C(85)-C(86)	122.9(16)
C(84)-C(85)-I(4)	112.5(11)
C(86)-C(85)-I(4)	124.6(13)
C(87)-C(86)-C(85)	116.8(17)
C(87)-C(86)-H(86)	121.6

C(85)-C(86)-H(86)	121.6
C(88)-C(87)-C(86)	121.7(16)
C(88)-C(87)-H(87)	119.2
C(86)-C(87)-H(87)	119.2
C(89)-C(88)-C(87)	120.2(16)
C(89)-C(88)-H(88)	119.9
C(87)-C(88)-H(88)	119.9
C(88)-C(89)-C(84)	119.0(17)
C(88)-C(89)-H(89)	120.5
C(84)-C(89)-H(89)	120.5
C(91)-C(90)-C(82)	114.0(12)
C(91)-C(90)-H(90A)	108.7
C(82)-C(90)-H(90A)	108.7
C(91)-C(90)-H(90B)	108.7
C(82)-C(90)-H(90B)	108.7
H(90A)-C(90)-H(90B)	107.6
C(96)-C(91)-C(92)	118.5(15)
C(96)-C(91)-C(90)	120.6(14)
C(92)-C(91)-C(90)	120.8(15)
C(93)-C(92)-C(91)	119.0(17)
C(93)-C(92)-H(92)	120.5
C(91)-C(92)-H(92)	120.5
C(94)-C(93)-C(92)	122.6(17)
C(94)-C(93)-H(93)	118.7
C(92)-C(93)-H(93)	118.7
C(95)-C(94)-C(93)	119.3(15)
C(95)-C(94)-H(94)	120.4
C(93)-C(94)-H(94)	120.4
C(94)-C(95)-C(96)	120.6(16)
C(94)-C(95)-H(95)	119.7
C(96)-C(95)-H(95)	119.7
C(91)-C(96)-C(95)	120.0(16)
C(91)-C(96)-H(96)	120.0
C(95)-C(96)-H(96)	120.0
C(98)-C(97)-C(102)	112.3(14)
C(98)-C(97)-B(1)	119.1(15)

C(102)-C(97)-B(1)	128.6(14)
C(99)-C(98)-F(1)	115.5(14)
C(99)-C(98)-C(97)	124.6(16)
F(1)-C(98)-C(97)	119.9(14)
C(100)-C(99)-F(2)	118.2(15)
C(100)-C(99)-C(98)	120.8(16)
F(2)-C(99)-C(98)	121.0(16)
F(3)-C(100)-C(99)	122.4(16)
F(3)-C(100)-C(101)	120.3(16)
C(99)-C(100)-C(101)	117.3(15)
F(4)-C(101)-C(102)	120.3(15)
F(4)-C(101)-C(100)	119.6(14)
C(102)-C(101)-C(100)	120.1(15)
F(5)-C(102)-C(101)	115.8(13)
F(5)-C(102)-C(97)	119.4(12)
C(101)-C(102)-C(97)	124.8(14)
C(108)-C(103)-C(104)	111.2(14)
C(108)-C(103)-B(1)	120.2(13)
C(104)-C(103)-B(1)	128.3(14)
C(105)-C(104)-F(6)	115.2(13)
C(105)-C(104)-C(103)	125.6(15)
F(6)-C(104)-C(103)	119.2(14)
F(7)-C(105)-C(106)	120.8(15)
F(7)-C(105)-C(104)	121.1(15)
C(106)-C(105)-C(104)	118.1(14)
C(105)-C(106)-C(107)	121.4(15)
C(105)-C(106)-F(8)	120.7(14)
C(107)-C(106)-F(8)	117.8(15)
F(9)-C(107)-C(106)	121.5(15)
F(9)-C(107)-C(108)	120.3(14)
C(106)-C(107)-C(108)	118.2(15)
F(10)-C(108)-C(103)	118.7(14)
F(10)-C(108)-C(107)	116.0(13)
C(103)-C(108)-C(107)	125.4(14)
C(114)-C(109)-C(110)	112.1(14)
C(114)-C(109)-B(1)	118.8(13)

C(110)-C(109)-B(1)	128.7(13)
C(111)-C(110)-F(11)	113.8(12)
C(111)-C(110)-C(109)	125.2(13)
F(11)-C(110)-C(109)	121.0(13)
F(12)-C(111)-C(110)	121.2(13)
F(12)-C(111)-C(112)	119.4(14)
C(110)-C(111)-C(112)	119.4(13)
F(13)-C(112)-C(111)	121.2(14)
F(13)-C(112)-C(113)	120.5(15)
C(111)-C(112)-C(113)	118.3(15)
F(14)-C(113)-C(114)	121.4(14)
F(14)-C(113)-C(112)	119.2(15)
C(114)-C(113)-C(112)	119.3(15)
C(113)-C(114)-C(109)	125.7(14)
C(113)-C(114)-F(15)	114.9(14)
C(109)-C(114)-F(15)	119.5(14)
C(116)-C(115)-C(120)	113.4(14)
C(116)-C(115)-B(1)	120.0(13)
C(120)-C(115)-B(1)	126.1(14)
F(16)-C(116)-C(115)	119.6(13)
F(16)-C(116)-C(117)	113.7(14)
C(115)-C(116)-C(117)	126.7(15)
F(17)-C(117)-C(116)	122.3(15)
F(17)-C(117)-C(118)	120.3(15)
C(116)-C(117)-C(118)	117.2(16)
C(119)-C(118)-F(18)	121.4(17)
C(119)-C(118)-C(117)	119.7(15)
F(18)-C(118)-C(117)	118.7(17)
C(118)-C(119)-F(19)	121.5(16)
C(118)-C(119)-C(120)	121.2(15)
F(19)-C(119)-C(120)	117.3(16)
F(20)-C(120)-C(115)	122.6(14)
F(20)-C(120)-C(119)	115.9(13)
C(115)-C(120)-C(119)	121.5(15)
C(115)-B(1)-C(109)	114.7(13)
C(115)-B(1)-C(97)	113.4(13)

C(109)-B(1)-C(97)	103.6(13)
C(115)-B(1)-C(103)	100.6(13)
C(109)-B(1)-C(103)	111.5(12)
C(97)-B(1)-C(103)	113.4(13)
O(18)-Li(1)-O(19)	107.7(12)
O(18)-Li(1)-O(6)#3	98.7(11)
O(19)-Li(1)-O(6)#3	111.6(12)
O(18)-Li(1)-O(15)#4	127.5(13)
O(19)-Li(1)-O(15)#4	108.6(12)
O(6)#3-Li(1)-O(15)#4	101.7(11)
C(122)-C(121)-H(12A)	109.5
C(122)-C(121)-H(12B)	109.5
H(12A)-C(121)-H(12B)	109.5
C(122)-C(121)-H(12C)	109.5
H(12A)-C(121)-H(12C)	109.5
H(12B)-C(121)-H(12C)	109.5
O(20)-C(122)-C(121)	108.8
O(20)-C(122)-H(12D)	109.9
C(121)-C(122)-H(12D)	109.9
O(20)-C(122)-H(12E)	109.9
C(121)-C(122)-H(12E)	109.9
H(12D)-C(122)-H(12E)	108.3
C(122)-O(20)-C(123)	113.5
O(20)-C(123)-C(124)	108.8
O(20)-C(123)-H(12F)	109.9
C(124)-C(123)-H(12F)	109.9
O(20)-C(123)-H(12G)	109.9
C(124)-C(123)-H(12G)	109.9
H(12F)-C(123)-H(12G)	108.3
C(123)-C(124)-H(12H)	109.5
C(123)-C(124)-H(12I)	109.5
H(12H)-C(124)-H(12I)	109.5
C(123)-C(124)-H(12J)	109.5
H(12H)-C(124)-H(12J)	109.5
H(12I)-C(124)-H(12J)	109.5
C(126)-C(125)-H(12K)	109.5

C(126)-C(125)-H(12L)	109.5
H(12K)-C(125)-H(12L)	109.5
C(126)-C(125)-H(12M)	109.5
H(12K)-C(125)-H(12M)	109.5
H(12L)-C(125)-H(12M)	109.5
O(21)-C(126)-C(125)	108.8
O(21)-C(126)-H(12N)	109.9
C(125)-C(126)-H(12N)	109.9
O(21)-C(126)-H(12O)	109.9
C(125)-C(126)-H(12O)	109.9
H(12N)-C(126)-H(12O)	108.3
C(127)-O(21)-C(126)	113.5
O(21)-C(127)-C(128)	108.8
O(21)-C(127)-H(12P)	109.9
C(128)-C(127)-H(12P)	109.9
O(21)-C(127)-H(12Q)	109.9
C(128)-C(127)-H(12Q)	109.9
H(12P)-C(127)-H(12Q)	108.3
C(127)-C(128)-H(12R)	109.5
C(127)-C(128)-H(12S)	109.5
H(12R)-C(128)-H(12S)	109.5
C(127)-C(128)-H(12T)	109.5
H(12R)-C(128)-H(12T)	109.5
H(12S)-C(128)-H(12T)	109.5
C(130)-C(129)-H(12U)	109.5
C(130)-C(129)-H(12V)	109.5
H(12U)-C(129)-H(12V)	109.5
C(130)-C(129)-H(12W)	109.5
H(12U)-C(129)-H(12W)	109.5
H(12V)-C(129)-H(12W)	109.5
O(22)-C(130)-C(129)	108.8
O(22)-C(130)-H(13A)	109.9
C(129)-C(130)-H(13A)	109.9
O(22)-C(130)-H(13B)	109.9
C(129)-C(130)-H(13B)	109.9
H(13A)-C(130)-H(13B)	108.3

C(130)-O(22)-C(131)	113.5
O(22)-C(131)-C(132)	108.8
O(22)-C(131)-H(13C)	109.9
C(132)-C(131)-H(13C)	109.9
O(22)-C(131)-H(13D)	109.9
C(132)-C(131)-H(13D)	109.9
H(13C)-C(131)-H(13D)	108.3
C(131)-C(132)-H(13E)	109.5
C(131)-C(132)-H(13F)	109.5
H(13E)-C(132)-H(13F)	109.5
C(131)-C(132)-H(13G)	109.5
H(13E)-C(132)-H(13G)	109.5
H(13F)-C(132)-H(13G)	109.5

Symmetry transformations used to generate equivalent atoms:

#1 -x+1,y-1/2,-z+1/2   #2 -x+3/2,-y+1,z+1/2   #3 -x+1,y+1/2,-z+1/2

#4 -x+3/2,-y+1,z-1/2

Table 4. Anisotropic displacement parameters ($Å^2$x 10^3) for 19948ds. The anisotropic displacement factor exponent takes the form: $-2p^2[h^2 a^{*2}U^{11} + ... + 2 h k a^* b^* U^{12}]$

	U^{11}	U^{22}	U^{33}	U^{23}	U^{13}	U^{12}
I(1)	30(1)	34(1)	47(1)	-4(1)	-8(1)	3(1)
I(2)	27(1)	51(1)	35(1)	0(1)	-1(1)	5(1)
I(3)	33(1)	32(1)	42(1)	-7(1)	-8(1)	6(1)
I(4)	29(1)	34(1)	37(1)	3(1)	2(1)	1(1)
I(5)	34(1)	36(1)	29(1)	-2(1)	-5(1)	10(1)
I(6)	27(1)	26(1)	26(1)	1(1)	-1(1)	0(1)
F(1)	33(5)	32(5)	60(6)	7(4)	1(5)	8(4)
F(2)	40(6)	64(7)	82(8)	3(6)	-22(6)	20(5)
F(3)	44(6)	76(7)	70(8)	7(6)	-27(6)	-2(5)
F(4)	61(6)	48(6)	52(7)	-3(5)	-26(5)	-15(5)
F(5)	41(5)	33(5)	51(6)	-5(4)	-22(5)	-6(4)
F(6)	23(4)	54(6)	48(6)	0(5)	3(4)	-9(4)
F(7)	27(5)	70(7)	60(7)	7(6)	20(5)	1(5)
F(8)	45(6)	54(6)	52(7)	-11(5)	18(5)	-5(5)
F(9)	39(5)	60(6)	48(6)	-12(5)	0(5)	-11(5)
F(10)	28(5)	39(5)	42(6)	-13(4)	5(4)	-8(4)
F(11)	38(5)	28(4)	45(6)	-6(4)	11(4)	-6(4)
F(12)	49(6)	31(5)	55(6)	0(5)	16(5)	-4(4)
F(13)	80(7)	38(5)	43(6)	-2(5)	23(5)	-12(5)
F(14)	67(7)	49(6)	53(7)	-13(5)	32(6)	-6(5)
F(15)	35(5)	28(5)	43(5)	-5(4)	2(4)	-3(4)
F(16)	34(5)	40(5)	53(6)	8(5)	-12(4)	-11(4)
F(17)	80(8)	30(5)	53(6)	11(5)	-23(5)	-19(5)
F(18)	87(8)	35(6)	70(8)	1(5)	-35(6)	21(5)
F(19)	44(6)	72(7)	57(7)	-12(6)	-17(5)	29(5)
F(20)	23(4)	52(5)	46(6)	-2(5)	-4(4)	0(4)
O(1)	24(6)	50(7)	53(7)	-1(6)	-8(5)	-4(6)
O(2)	32(6)	61(8)	49(8)	6(7)	-7(6)	-3(6)
O(3)	48(7)	46(7)	45(8)	3(6)	0(6)	5(6)
O(4)	28(6)	50(7)	31(6)	3(6)	-4(5)	1(5)
O(5)	28(6)	52(7)	51(8)	-3(6)	-14(5)	-4(6)

O(6)	25(6)	53(7)	38(7)	-10(6)	-2(5)	5(5)
O(7)	44(7)	31(6)	54(8)	1(6)	-20(6)	7(5)
O(8)	37(7)	56(8)	55(9)	-5(7)	-15(6)	-4(6)
O(9)	43(7)	33(7)	68(9)	0(6)	-14(7)	-4(6)
O(10)	29(6)	51(7)	41(7)	9(6)	-1(5)	11(5)
O(11)	39(7)	46(7)	36(7)	11(5)	2(6)	16(5)
O(12)	32(6)	53(7)	40(7)	-5(6)	-6(5)	23(5)
O(13)	51(7)	40(7)	31(7)	13(5)	-1(5)	11(6)
O(14)	46(7)	39(6)	35(7)	14(6)	10(6)	13(5)
O(15)	37(6)	34(6)	25(6)	5(5)	7(5)	5(5)
O(16)	28(6)	37(6)	27(6)	0(5)	-4(5)	1(5)
O(17)	29(6)	33(6)	36(7)	11(5)	6(5)	7(5)
O(18)	31(6)	38(6)	41(7)	18(6)	-3(5)	-8(5)
O(19)	29(6)	37(6)	37(7)	-3(5)	-3(5)	-4(5)
N(1)	33(7)	45(8)	40(9)	10(7)	4(7)	-1(6)
N(2)	32(7)	40(8)	33(8)	-8(7)	-11(6)	10(6)
N(3)	35(8)	36(8)	55(10)	4(7)	-14(7)	-10(6)
N(4)	26(7)	48(8)	29(8)	-9(7)	-9(6)	4(6)
N(5)	25(6)	19(6)	34(8)	6(6)	3(6)	0(5)
N(6)	30(7)	29(7)	31(8)	5(6)	-6(6)	-9(6)
C(1)	37(10)	38(9)	36(10)	-3(8)	-9(8)	8(8)
C(2)	21(8)	55(11)	46(11)	-8(9)	-12(8)	13(8)
C(3)	34(9)	38(9)	33(10)	-1(8)	-2(8)	6(8)
C(4)	32(9)	33(9)	47(11)	-4(8)	7(8)	5(7)
C(5)	33(9)	60(11)	27(9)	-3(8)	-5(8)	0(9)
C(6)	30(9)	50(10)	46(11)	2(9)	-2(8)	7(8)
C(7)	36(10)	58(12)	45(12)	2(10)	16(9)	1(9)
C(8)	47(11)	52(11)	42(12)	-5(9)	14(9)	2(9)
C(9)	29(9)	55(11)	37(10)	-3(9)	4(8)	8(8)
C(10)	29(9)	45(10)	52(12)	7(9)	4(8)	0(7)
C(11)	34(9)	34(9)	71(14)	9(9)	5(9)	6(8)
C(12)	40(10)	62(12)	57(14)	15(10)	13(9)	14(9)
C(13)	66(14)	63(13)	58(15)	0(11)	1(11)	-4(11)
C(14)	83(17)	83(17)	68(16)	14(14)	1(13)	-1(14)
C(15)	73(15)	73(15)	61(15)	14(12)	31(12)	-9(13)
C(16)	60(12)	63(12)	40(12)	3(10)	10(10)	-9(10)

C(17)	37(10)	47(10)	28(10)	2(8)	-10(8)	18(9)
C(18)	35(9)	40(9)	23(9)	1(7)	9(7)	12(8)
C(19)	33(9)	29(8)	41(10)	-18(8)	0(8)	12(8)
C(20)	33(9)	24(8)	44(11)	-9(8)	-7(8)	3(7)
C(21)	46(10)	20(8)	36(10)	-6(7)	4(8)	13(7)
C(22)	35(10)	32(9)	51(12)	6(8)	0(8)	9(8)
C(23)	74(14)	40(10)	38(11)	9(9)	0(10)	21(9)
C(24)	37(10)	55(11)	28(10)	-5(8)	-6(8)	9(8)
C(25)	37(10)	39(9)	42(11)	-5(8)	1(8)	10(8)
C(26)	22(8)	46(10)	46(11)	-1(8)	-2(8)	11(7)
C(27)	41(8)	47(8)	51(8)	2(7)	7(8)	-5(8)
C(28)	45(9)	47(8)	61(8)	9(7)	3(8)	-5(8)
C(29)	47(10)	53(8)	69(9)	11(8)	3(9)	-3(9)
C(30)	48(11)	55(9)	68(9)	11(8)	4(9)	-4(9)
C(31)	51(9)	53(9)	63(8)	10(8)	5(8)	-5(8)
C(32)	47(8)	49(9)	56(8)	3(8)	9(8)	-4(8)
C(27A)	44(9)	47(8)	54(9)	4(8)	7(8)	-5(8)
C(28A)	46(10)	48(8)	60(9)	9(8)	6(9)	-5(9)
C(29A)	48(10)	52(8)	67(9)	10(8)	5(9)	-2(9)
C(30A)	50(11)	54(9)	67(9)	9(9)	6(9)	-1(9)
C(31A)	50(10)	54(10)	63(9)	7(9)	7(9)	-2(9)
C(32A)	49(9)	51(9)	58(9)	3(9)	7(8)	-3(9)
C(33)	45(11)	28(9)	55(13)	2(9)	-6(10)	4(8)
C(34)	27(9)	27(8)	75(13)	-3(9)	-19(9)	-7(7)
C(35)	37(10)	28(9)	68(14)	-4(9)	3(9)	0(8)
C(36)	35(9)	25(8)	57(12)	-5(9)	-17(9)	5(7)
C(37)	37(9)	35(9)	40(11)	8(8)	-13(8)	6(8)
C(38)	36(10)	45(10)	51(12)	-6(9)	-2(9)	5(8)
C(39)	47(11)	42(11)	88(16)	-13(11)	-24(12)	4(9)
C(40)	34(10)	37(10)	104(18)	14(11)	-31(11)	-2(9)
C(41)	49(11)	29(9)	88(17)	10(10)	-11(11)	2(9)
C(42)	33(9)	42(10)	44(11)	-2(9)	-17(8)	-7(8)
C(43)	27(8)	33(9)	46(11)	3(8)	-7(8)	-2(7)
C(44)	58(12)	49(11)	65(14)	-1(11)	-22(11)	14(10)
C(45)	61(12)	65(13)	59(14)	-11(12)	-27(11)	8(11)
C(46)	67(13)	37(10)	48(12)	-6(9)	0(10)	12(10)

C(47)	57(11)	43(10)	37(10)	16(8)	-5(9)	-15(9)
C(48)	43(10)	28(9)	49(11)	-2(8)	-8(9)	8(8)
C(49)	29(9)	48(11)	48(12)	-7(9)	-2(9)	13(8)
C(50)	19(8)	38(9)	41(10)	2(8)	-2(7)	1(7)
C(51)	46(11)	42(10)	29(10)	-2(8)	-3(8)	15(8)
C(52)	32(9)	45(10)	28(9)	-10(8)	-12(7)	11(8)
C(53)	34(9)	32(9)	34(10)	-2(7)	-12(7)	8(7)
C(54)	37(9)	48(10)	39(10)	3(9)	-19(8)	0(8)
C(55)	47(11)	58(11)	36(10)	9(9)	-6(9)	-5(9)
C(56)	43(10)	43(10)	54(13)	-14(9)	-23(9)	10(8)
C(57)	39(10)	52(11)	44(12)	-13(9)	-3(9)	14(9)
C(58)	31(9)	53(11)	29(10)	-1(8)	2(7)	8(8)
C(59)	30(9)	41(10)	45(11)	-9(8)	1(8)	-9(8)
C(60)	38(10)	41(9)	42(10)	-7(8)	-6(8)	9(8)
C(61)	47(12)	73(13)	48(12)	10(10)	0(10)	-9(10)
C(62)	29(9)	54(12)	86(16)	-17(12)	-1(10)	-3(9)
C(63)	38(11)	63(12)	51(12)	14(10)	-8(9)	6(9)
C(64)	45(11)	46(10)	43(11)	1(9)	-11(9)	13(9)
C(65)	24(8)	36(9)	32(10)	8(8)	-3(7)	3(7)
C(66)	35(9)	30(8)	34(10)	8(7)	-4(8)	13(7)
C(67)	26(8)	35(8)	32(10)	-3(8)	4(7)	-6(7)
C(68)	41(9)	32(9)	30(9)	6(7)	-8(8)	1(7)
C(69)	30(8)	28(8)	47(10)	-6(8)	-19(8)	10(7)
C(70)	67(12)	51(10)	24(9)	13(9)	6(9)	6(9)
C(71)	124(19)	58(12)	42(12)	23(10)	19(12)	61(13)
C(72)	97(16)	56(12)	48(13)	6(10)	16(11)	57(12)
C(73)	53(11)	44(10)	29(9)	-1(8)	14(8)	21(9)
C(74)	34(9)	46(10)	23(9)	1(7)	3(7)	-1(8)
C(75)	39(9)	22(8)	36(10)	0(7)	-1(8)	-8(7)
C(76)	37(10)	44(10)	39(11)	-1(8)	10(8)	0(8)
C(77)	46(11)	46(11)	47(12)	4(10)	-9(9)	4(9)
C(78)	52(11)	52(11)	36(11)	-9(9)	-15(9)	10(9)
C(79)	74(13)	50(11)	31(10)	-19(9)	4(10)	-2(11)
C(80)	45(10)	34(9)	37(11)	-9(8)	-3(8)	11(8)
C(81)	21(8)	41(9)	24(9)	-1(8)	-1(7)	-3(7)
C(82)	25(8)	34(9)	35(10)	10(7)	-8(7)	-1(7)

C(83)	35(9)	28(8)	39(10)	-3(8)	-2(8)	-2(7)
C(84)	35(9)	37(9)	39(10)	-2(8)	8(8)	-14(8)
C(85)	32(9)	45(10)	36(10)	1(9)	-2(7)	-5(8)
C(86)	29(9)	65(12)	58(12)	3(10)	4(9)	-1(9)
C(87)	16(8)	72(12)	65(14)	17(11)	14(8)	2(8)
C(88)	28(9)	64(12)	57(13)	0(10)	22(9)	-7(9)
C(89)	40(10)	47(10)	53(12)	13(10)	14(9)	-7(9)
C(90)	35(9)	33(9)	25(9)	5(7)	3(7)	0(7)
C(91)	19(8)	40(9)	40(10)	11(8)	-5(7)	7(7)
C(92)	39(10)	39(10)	52(12)	1(9)	-5(8)	-3(8)
C(93)	53(11)	44(11)	58(13)	24(10)	-6(10)	-17(9)
C(94)	49(11)	21(8)	72(15)	-9(9)	-16(11)	-6(8)
C(95)	27(9)	38(10)	56(12)	-10(9)	-5(8)	11(8)
C(96)	36(9)	46(10)	33(10)	9(9)	-1(8)	6(8)
C(97)	24(8)	56(11)	31(9)	2(9)	-2(7)	-2(8)
C(98)	28(9)	39(10)	52(11)	1(8)	-8(8)	11(8)
C(99)	36(10)	52(11)	52(12)	11(10)	-1(9)	5(9)
C(100)	25(9)	74(14)	39(11)	4(10)	-12(8)	-1(9)
C(101)	35(9)	43(10)	45(11)	-10(9)	-9(8)	-18(8)
C(102)	26(8)	30(8)	35(9)	3(7)	-19(7)	2(7)
C(103)	23(8)	35(8)	37(9)	7(7)	14(7)	1(7)
C(104)	21(8)	30(9)	49(11)	8(8)	1(8)	-2(7)
C(105)	29(9)	44(10)	47(11)	3(8)	11(8)	5(8)
C(106)	49(11)	37(9)	29(10)	-4(7)	10(8)	-2(8)
C(107)	34(9)	44(9)	39(10)	-8(8)	5(9)	1(8)
C(108)	23(8)	38(9)	35(10)	6(8)	2(7)	-2(7)
C(109)	28(8)	23(8)	28(9)	-1(7)	6(7)	3(6)
C(110)	17(7)	31(9)	29(9)	-14(7)	1(6)	2(7)
C(111)	30(8)	22(8)	45(11)	-11(7)	7(8)	-7(7)
C(112)	31(9)	39(10)	43(11)	1(8)	5(8)	-10(7)
C(113)	46(10)	42(10)	23(9)	-7(8)	1(8)	-6(8)
C(114)	32(9)	29(9)	51(11)	-1(9)	-5(8)	5(7)
C(115)	43(9)	27(8)	24(9)	0(7)	-14(7)	2(7)
C(116)	29(9)	32(9)	36(10)	3(8)	-18(8)	-2(7)
C(117)	48(11)	28(9)	47(11)	-5(8)	-22(9)	-4(8)
C(118)	69(13)	23(9)	38(11)	-13(8)	-28(9)	2(9)

C(119)	29(9)	41(10)	49(11)	-13(9)	-22(8)	9(8)
C(120)	24(8)	29(9)	48(11)	-16(8)	-5(7)	0(7)
B(1)	32(10)	37(10)	37(11)	-1(9)	5(8)	-11(8)
Li(1)	25(12)	19(12)	28(14)	-6(11)	8(10)	-3(10)
C(121)	140(20)	43(14)	69(14)	-21(13)	51(16)	-8(15)
C(122)	123(15)	41(10)	71(12)	-4(11)	37(12)	13(10)
O(20)	102(11)	34(8)	55(9)	-2(7)	30(9)	5(8)
C(123)	93(13)	44(10)	50(10)	2(10)	24(10)	1(9)
C(124)	43(12)	53(12)	22(11)	-4(10)	2(10)	-15(9)
C(125)	52(18)	120(30)	70(20)	-44(19)	-26(17)	6(16)
C(126)	76(16)	103(19)	58(17)	-25(15)	-19(13)	10(14)
O(21)	75(14)	105(18)	72(15)	-14(13)	-19(12)	1(12)
C(127)	71(16)	106(19)	84(18)	-5(16)	-16(14)	4(14)
C(128)	70(20)	120(30)	100(20)	-20(20)	-18(19)	10(20)
C(129)	100(19)	35(18)	160(30)	10(20)	40(20)	-21(17)
C(130)	111(18)	38(14)	130(20)	-1(14)	12(17)	-7(13)
O(22)	108(16)	33(11)	130(20)	-2(12)	11(16)	4(11)
C(131)	106(17)	20(12)	120(20)	5(13)	-3(17)	11(13)
C(132)	90(20)	25(16)	110(30)	-12(17)	6(19)	27(16)

Table 5. Hydrogen coordinates (x 10^4) and isotropic displacement parameters ($Å^2$x 10^3) for 19948ds.

	x	y	z	U(eq)
H(19A)	8101	7018	768	52
H(19B)	8786	6811	896	52
H(2)	5661	-182	7682	49
H(6)	2898	-930	6528	50
H(7)	2385	-1624	6998	56
H(8)	2854	-1971	7740	56
H(9)	3845	-1602	8030	48
H(10A)	6447	-495	7044	50
H(10B)	6027	-993	7287	50
H(12)	5274	-1521	6782	64
H(13)	4858	-1750	5970	75
H(14)	5059	-1152	5279	94
H(15)	5719	-383	5390	83
H(16)	6195	-191	6169	65
H(18)	2731	217	5330	39
H(22)	4822	1915	4178	47
H(23)	4391	2134	3371	61
H(24)	3371	1820	3112	48
H(25)	2751	1244	3639	47
H(26A)	3167	-329	4626	46
H(26B)	3808	-439	4964	46
H(28)	3882	-1272	5319	61
H(29)	3434	-2091	5655	67
H(30)	2291	-2182	5741	68
H(31)	1594	-1455	5493	67
H(32)	2042	-637	5157	61
H(28A)	3723	-1391	5378	62
H(29A)	3055	-2106	5694	66
H(30A)	1909	-1996	5713	68
H(31A)	1430	-1173	5417	67

H(32A)	2097	-458	5101	63
H(34)	6207	2268	5342	52
H(38)	7157	950	7389	53
H(39)	8213	1287	7617	71
H(40)	8778	1895	7133	70
H(41)	8350	2180	6364	66
H(42A)	7045	1819	4899	47
H(42B)	6364	1781	4594	47
H(44)	7512	983	5295	69
H(45)	7652	22	5216	74
H(46)	6927	-506	4759	61
H(47)	6012	-72	4366	55
H(48)	5878	876	4429	48
H(50)	10353	2908	3081	39
H(54)	8299	2275	4913	49
H(55)	8720	1550	5418	56
H(56)	9735	1135	5186	56
H(57)	10354	1474	4494	54
H(58A)	10087	2447	2335	45
H(58B)	9993	1988	2771	45
H(60)	8797	1750	3043	48
H(61)	7716	1646	2780	67
H(62)	7340	2138	2050	67
H(63)	8084	2683	1584	61
H(64)	9171	2758	1840	54
H(66)	7026	3597	4894	39
H(70)	6188	5558	3277	57
H(71)	5379	6121	3672	90
H(72)	5082	6000	4502	80
H(73)	5440	5204	4957	50
H(74A)	6597	2752	4539	41
H(74B)	6028	3186	4680	41
H(76)	5303	3537	4032	48
H(77)	4922	3540	3208	56
H(78)	5516	3094	2569	56
H(79)	6485	2624	2775	62

H(80)	6890	2626	3597	46
H(82)	7740	5468	2205	37
H(86)	10319	3902	1827	61
H(87)	10991	4320	1204	61
H(88)	10616	5073	730	60
H(89)	9617	5495	935	56
H(90A)	6743	5082	1885	37
H(90B)	7268	5176	1439	37
H(92)	6447	4111	2152	52
H(93)	6323	3202	1873	62
H(94)	6889	2865	1178	56
H(95)	7607	3438	743	48
H(96)	7736	4376	987	46
H(12A)	10740	2343	-746	125
H(12B)	9998	2554	-820	125
H(12C)	10581	2995	-749	125
H(12D)	10153	2242	29	94
H(12E)	10738	2684	101	94
H(12F)	10124	3129	780	75
H(12G)	9539	2686	708	75
H(12H)	9113	3522	1055	59
H(12I)	9385	3861	574	59
H(12J)	8802	3420	502	59
H(12K)	8366	2093	794	119
H(12L)	8349	1739	278	119
H(12M)	7881	2270	341	119
H(12N)	7746	1270	924	95
H(12O)	7277	1803	987	95
H(12P)	6331	1247	747	104
H(12Q)	6800	713	684	104
H(12R)	5858	617	156	144
H(12S)	6038	1186	-127	144
H(12T)	6505	654	-190	144
H(12U)	1210	6534	5768	149
H(12V)	1291	6694	6356	149
H(12W)	1439	7147	5925	149

H(13A)	2216	6167	6082	113
H(13B)	2365	6622	5650	113
H(13C)	3432	6805	6030	99
H(13D)	3283	6351	6462	99
H(13E)	4040	7020	6776	114
H(13F)	3517	7504	6666	114
H(13G)	3370	7050	7097	114

Table 6. Torsion angles [°] for 19948ds.

I(1)-O(1)-C(1)-O(2)	-15.8(18)
I(1)-O(1)-C(1)-C(2)	159.9(12)
C(3)-N(1)-C(2)-C(1)	-168.0(14)
I(2)-N(1)-C(2)-C(1)	31.9(17)
C(3)-N(1)-C(2)-C(10)	71(2)
I(2)-N(1)-C(2)-C(10)	-88.7(15)
O(2)-C(1)-C(2)-N(1)	-13(2)
O(1)-C(1)-C(2)-N(1)	170.9(14)
O(2)-C(1)-C(2)-C(10)	110.1(17)
O(1)-C(1)-C(2)-C(10)	-65.7(18)
C(2)-N(1)-C(3)-O(3)	14(2)
I(2)-N(1)-C(3)-O(3)	175.5(12)
C(2)-N(1)-C(3)-C(4)	-164.9(14)
I(2)-N(1)-C(3)-C(4)	-3.6(17)
O(3)-C(3)-C(4)-C(5)	-174.0(15)
N(1)-C(3)-C(4)-C(5)	5(2)
O(3)-C(3)-C(4)-C(9)	6(2)
N(1)-C(3)-C(4)-C(9)	-175.2(14)
C(9)-C(4)-C(5)-C(6)	-2(2)
C(3)-C(4)-C(5)-C(6)	177.5(14)
C(9)-C(4)-C(5)-I(2)	176.4(11)
C(3)-C(4)-C(5)-I(2)	-4.0(19)
C(4)-C(5)-C(6)-C(7)	1(3)
I(2)-C(5)-C(6)-C(7)	-177.3(12)
C(5)-C(6)-C(7)-C(8)	0(3)
C(6)-C(7)-C(8)-C(9)	1(3)
C(7)-C(8)-C(9)-C(4)	-2(2)
C(5)-C(4)-C(9)-C(8)	2(2)
C(3)-C(4)-C(9)-C(8)	-177.2(14)
N(1)-C(2)-C(10)-C(11)	50.5(19)
C(1)-C(2)-C(10)-C(11)	-71.5(17)
C(2)-C(10)-C(11)-C(16)	82(2)
C(2)-C(10)-C(11)-C(12)	-92.6(18)
C(16)-C(11)-C(12)-C(13)	0(3)

C(10)-C(11)-C(12)-C(13)	174.9(16)
C(11)-C(12)-C(13)-C(14)	-3(3)
C(12)-C(13)-C(14)-C(15)	2(3)
C(13)-C(14)-C(15)-C(16)	0(3)
C(12)-C(11)-C(16)-C(15)	2(3)
C(10)-C(11)-C(16)-C(15)	-172.2(17)
C(14)-C(15)-C(16)-C(11)	-3(3)
I(2)-O(4)-C(17)-O(5)	-8.5(18)
I(2)-O(4)-C(17)-C(18)	167.1(10)
C(19)-N(2)-C(18)-C(17)	-165.0(12)
I(3)-N(2)-C(18)-C(17)	16.5(16)
C(19)-N(2)-C(18)-C(26)	74.6(17)
I(3)-N(2)-C(18)-C(26)	-103.9(13)
O(4)-C(17)-C(18)-N(2)	172.3(13)
O(5)-C(17)-C(18)-N(2)	-11.8(19)
O(4)-C(17)-C(18)-C(26)	-65.4(17)
O(5)-C(17)-C(18)-C(26)	110.5(16)
Li(1)#1-O(6)-C(19)-N(2)	132.6(17)
Li(1)#1-O(6)-C(19)-C(20)	-54(2)
C(18)-N(2)-C(19)-O(6)	7(2)
I(3)-N(2)-C(19)-O(6)	-174.5(11)
C(18)-N(2)-C(19)-C(20)	-167.4(12)
I(3)-N(2)-C(19)-C(20)	11.3(15)
O(6)-C(19)-C(20)-C(25)	-4(2)
N(2)-C(19)-C(20)-C(25)	170.1(14)
O(6)-C(19)-C(20)-C(21)	179.2(13)
N(2)-C(19)-C(20)-C(21)	-6.7(19)
C(25)-C(20)-C(21)-C(22)	0(2)
C(19)-C(20)-C(21)-C(22)	176.7(13)
C(25)-C(20)-C(21)-I(3)	-178.2(11)
C(19)-C(20)-C(21)-I(3)	-1.3(16)
C(20)-C(21)-C(22)-C(23)	-1(2)
I(3)-C(21)-C(22)-C(23)	176.3(11)
C(21)-C(22)-C(23)-C(24)	2(2)
C(22)-C(23)-C(24)-C(25)	-1(2)
C(21)-C(20)-C(25)-C(24)	1(2)

C(19)-C(20)-C(25)-C(24)	-175.5(14)
C(23)-C(24)-C(25)-C(20)	-1(2)
N(2)-C(18)-C(26)-C(27A)	-167(4)
C(17)-C(18)-C(26)-C(27A)	72(4)
N(2)-C(18)-C(26)-C(27)	-168.9(18)
C(17)-C(18)-C(26)-C(27)	70(2)
C(18)-C(26)-C(27)-C(28)	-120.2(18)
C(18)-C(26)-C(27)-C(32)	62(3)
C(32)-C(27)-C(28)-C(29)	0.0
C(26)-C(27)-C(28)-C(29)	-178(3)
C(27)-C(28)-C(29)-C(30)	0.0
C(28)-C(29)-C(30)-C(31)	0.0
C(29)-C(30)-C(31)-C(32)	0.0
C(30)-C(31)-C(32)-C(27)	0.0
C(28)-C(27)-C(32)-C(31)	0.0
C(26)-C(27)-C(32)-C(31)	178(4)
C(18)-C(26)-C(27A)-C(28A)	-122(5)
C(18)-C(26)-C(27A)-C(32A)	60(5)
C(32A)-C(27A)-C(28A)-C(29A)	0.0
C(26)-C(27A)-C(28A)-C(29A)	-178(8)
C(27A)-C(28A)-C(29A)-C(30A)	0.0
C(28A)-C(29A)-C(30A)-C(31A)	0.0
C(29A)-C(30A)-C(31A)-C(32A)	0.0
C(30A)-C(31A)-C(32A)-C(27A)	0.0
C(28A)-C(27A)-C(32A)-C(31A)	0.0
C(26)-C(27A)-C(32A)-C(31A)	178(7)
I(3)-O(7)-C(33)-O(8)	-9.0(19)
I(3)-O(7)-C(33)-C(34)	162.2(11)
C(35)-N(3)-C(34)-C(42)	64.6(18)
I(1)-N(3)-C(34)-C(42)	-112.5(14)
C(35)-N(3)-C(34)-C(33)	-168.3(14)
I(1)-N(3)-C(34)-C(33)	14.5(18)
O(8)-C(33)-C(34)-N(3)	-8(2)
O(7)-C(33)-C(34)-N(3)	-178.8(13)
O(8)-C(33)-C(34)-C(42)	119.9(18)
O(7)-C(33)-C(34)-C(42)	-51.4(19)

C(34)-N(3)-C(35)-O(9)	8(3)
I(1)-N(3)-C(35)-O(9)	-174.6(13)
C(34)-N(3)-C(35)-C(36)	-173.4(14)
I(1)-N(3)-C(35)-C(36)	4.0(18)
O(9)-C(35)-C(36)-C(37)	175.0(16)
N(3)-C(35)-C(36)-C(37)	-4(2)
O(9)-C(35)-C(36)-C(41)	-3(3)
N(3)-C(35)-C(36)-C(41)	178.1(16)
C(41)-C(36)-C(37)-C(38)	4(3)
C(35)-C(36)-C(37)-C(38)	-174.7(15)
C(41)-C(36)-C(37)-I(1)	179.7(12)
C(35)-C(36)-C(37)-I(1)	1.3(19)
C(36)-C(37)-C(38)-C(39)	-3(2)
I(1)-C(37)-C(38)-C(39)	-178.4(12)
C(37)-C(38)-C(39)-C(40)	2(3)
C(38)-C(39)-C(40)-C(41)	-1(3)
C(39)-C(40)-C(41)-C(36)	2(3)
C(37)-C(36)-C(41)-C(40)	-3(3)
C(35)-C(36)-C(41)-C(40)	175.2(17)
N(3)-C(34)-C(42)-C(43)	63.7(17)
C(33)-C(34)-C(42)-C(43)	-61.2(18)
C(34)-C(42)-C(43)-C(48)	97.1(19)
C(34)-C(42)-C(43)-C(44)	-88.6(19)
C(48)-C(43)-C(44)-C(45)	-2(3)
C(42)-C(43)-C(44)-C(45)	-176.0(18)
C(43)-C(44)-C(45)-C(46)	1(3)
C(44)-C(45)-C(46)-C(47)	0(3)
C(45)-C(46)-C(47)-C(48)	1(3)
C(46)-C(47)-C(48)-C(43)	-2(3)
C(44)-C(43)-C(48)-C(47)	2(3)
C(42)-C(43)-C(48)-C(47)	176.5(16)
I(4)-O(10)-C(49)-O(11)	-3.5(19)
I(4)-O(10)-C(49)-C(50)	172.5(11)
C(51)-N(4)-C(50)-C(49)	-163.6(14)
I(5)-N(4)-C(50)-C(49)	13.0(17)
C(51)-N(4)-C(50)-C(58)	75.8(17)

I(5)-N(4)-C(50)-C(58)	-107.5(12)
O(11)-C(49)-C(50)-N(4)	-11(2)
O(10)-C(49)-C(50)-N(4)	173.1(14)
O(11)-C(49)-C(50)-C(58)	111.8(17)
O(10)-C(49)-C(50)-C(58)	-64.3(18)
C(50)-N(4)-C(51)-O(12)	7(2)
I(5)-N(4)-C(51)-O(12)	-169.6(12)
C(50)-N(4)-C(51)-C(52)	-172.2(13)
I(5)-N(4)-C(51)-C(52)	10.9(17)
O(12)-C(51)-C(52)-C(53)	173.6(15)
N(4)-C(51)-C(52)-C(53)	-7(2)
O(12)-C(51)-C(52)-C(57)	-8(3)
N(4)-C(51)-C(52)-C(57)	171.3(15)
C(57)-C(52)-C(53)-C(54)	5(2)
C(51)-C(52)-C(53)-C(54)	-176.5(14)
C(57)-C(52)-C(53)-I(5)	-178.8(12)
C(51)-C(52)-C(53)-I(5)	-0.6(18)
C(52)-C(53)-C(54)-C(55)	-6(2)
I(5)-C(53)-C(54)-C(55)	178.6(12)
C(53)-C(54)-C(55)-C(56)	2(2)
C(54)-C(55)-C(56)-C(57)	2(2)
C(53)-C(52)-C(57)-C(56)	-1(2)
C(51)-C(52)-C(57)-C(56)	-178.8(15)
C(55)-C(56)-C(57)-C(52)	-3(2)
N(4)-C(50)-C(58)-C(59)	74.4(16)
C(49)-C(50)-C(58)-C(59)	-48.4(18)
C(50)-C(58)-C(59)-C(60)	-81.4(19)
C(50)-C(58)-C(59)-C(64)	95.4(18)
C(64)-C(59)-C(60)-C(61)	1(2)
C(58)-C(59)-C(60)-C(61)	178.2(16)
C(59)-C(60)-C(61)-C(62)	-2(3)
C(60)-C(61)-C(62)-C(63)	2(3)
C(61)-C(62)-C(63)-C(64)	-1(3)
C(62)-C(63)-C(64)-C(59)	0(3)
C(60)-C(59)-C(64)-C(63)	0(2)
C(58)-C(59)-C(64)-C(63)	-177.2(16)

I(5)-O(13)-C(65)-O(14)	-1.9(18)
I(5)-O(13)-C(65)-C(66)	176.5(11)
C(67)-N(5)-C(66)-C(74)	77.8(17)
I(6)-N(5)-C(66)-C(74)	-96.7(13)
C(67)-N(5)-C(66)-C(65)	-159.0(13)
I(6)-N(5)-C(66)-C(65)	26.6(16)
O(14)-C(65)-C(66)-N(5)	-14(2)
O(13)-C(65)-C(66)-N(5)	167.7(13)
O(14)-C(65)-C(66)-C(74)	111.2(17)
O(13)-C(65)-C(66)-C(74)	-67.3(17)
Li(1)#2-O(15)-C(67)-N(5)	51(2)
Li(1)#2-O(15)-C(67)-C(68)	-127.7(16)
C(66)-N(5)-C(67)-O(15)	10(2)
I(6)-N(5)-C(67)-O(15)	-175.1(11)
C(66)-N(5)-C(67)-C(68)	-171.2(12)
I(6)-N(5)-C(67)-C(68)	3.5(16)
O(15)-C(67)-C(68)-C(73)	-3(2)
N(5)-C(67)-C(68)-C(73)	178.6(14)
O(15)-C(67)-C(68)-C(69)	180.0(14)
N(5)-C(67)-C(68)-C(69)	1(2)
C(73)-C(68)-C(69)-C(70)	4(2)
C(67)-C(68)-C(69)-C(70)	-179.0(14)
C(73)-C(68)-C(69)-I(6)	177.3(12)
C(67)-C(68)-C(69)-I(6)	-5.5(18)
C(68)-C(69)-C(70)-C(71)	-5(3)
I(6)-C(69)-C(70)-C(71)	-177.6(14)
C(69)-C(70)-C(71)-C(72)	6(3)
C(70)-C(71)-C(72)-C(73)	-5(4)
C(71)-C(72)-C(73)-C(68)	3(3)
C(69)-C(68)-C(73)-C(72)	-3(2)
C(67)-C(68)-C(73)-C(72)	-179.7(16)
N(5)-C(66)-C(74)-C(75)	53.7(18)
C(65)-C(66)-C(74)-C(75)	-69.4(16)
C(66)-C(74)-C(75)-C(80)	80.8(18)
C(66)-C(74)-C(75)-C(76)	-96.7(16)
C(80)-C(75)-C(76)-C(77)	-1(2)

C(74)-C(75)-C(76)-C(77)	176.8(15)
C(75)-C(76)-C(77)-C(78)	0(3)
C(76)-C(77)-C(78)-C(79)	1(3)
C(77)-C(78)-C(79)-C(80)	-1(3)
C(78)-C(79)-C(80)-C(75)	1(3)
C(76)-C(75)-C(80)-C(79)	0(2)
C(74)-C(75)-C(80)-C(79)	-177.4(15)
I(6)-O(16)-C(81)-O(17)	3.0(16)
I(6)-O(16)-C(81)-C(82)	-178.8(10)
C(83)-N(6)-C(82)-C(81)	-166.7(13)
I(4)-N(6)-C(82)-C(81)	11.0(16)
C(83)-N(6)-C(82)-C(90)	70.6(17)
I(4)-N(6)-C(82)-C(90)	-111.6(12)
O(17)-C(81)-C(82)-N(6)	-14.0(19)
O(16)-C(81)-C(82)-N(6)	167.7(12)
O(17)-C(81)-C(82)-C(90)	110.3(15)
O(16)-C(81)-C(82)-C(90)	-67.9(17)
Li(1)-O(18)-C(83)-N(6)	-139.7(17)
Li(1)-O(18)-C(83)-C(84)	41(3)
C(82)-N(6)-C(83)-O(18)	-1(2)
I(4)-N(6)-C(83)-O(18)	-178.7(11)
C(82)-N(6)-C(83)-C(84)	178.6(12)
I(4)-N(6)-C(83)-C(84)	0.6(16)
O(18)-C(83)-C(84)-C(85)	178.8(15)
N(6)-C(83)-C(84)-C(85)	0(2)
O(18)-C(83)-C(84)-C(89)	3(2)
N(6)-C(83)-C(84)-C(89)	-176.4(15)
C(89)-C(84)-C(85)-C(86)	-3(3)
C(83)-C(84)-C(85)-C(86)	-179.3(15)
C(89)-C(84)-C(85)-I(4)	176.1(13)
C(83)-C(84)-C(85)-I(4)	0.1(19)
C(84)-C(85)-C(86)-C(87)	1(3)
I(4)-C(85)-C(86)-C(87)	-178.0(13)
C(85)-C(86)-C(87)-C(88)	-1(3)
C(86)-C(87)-C(88)-C(89)	4(3)
C(87)-C(88)-C(89)-C(84)	-5(3)

C(85)-C(84)-C(89)-C(88)	5(3)
C(83)-C(84)-C(89)-C(88)	-178.8(16)
N(6)-C(82)-C(90)-C(91)	57.1(17)
C(81)-C(82)-C(90)-C(91)	-66.4(17)
C(82)-C(90)-C(91)-C(96)	-92.1(17)
C(82)-C(90)-C(91)-C(92)	88.8(17)
C(96)-C(91)-C(92)-C(93)	-2(2)
C(90)-C(91)-C(92)-C(93)	177.6(15)
C(91)-C(92)-C(93)-C(94)	1(3)
C(92)-C(93)-C(94)-C(95)	0(3)
C(93)-C(94)-C(95)-C(96)	-1(3)
C(92)-C(91)-C(96)-C(95)	1(2)
C(90)-C(91)-C(96)-C(95)	-178.6(14)
C(94)-C(95)-C(96)-C(91)	1(2)
C(102)-C(97)-C(98)-C(99)	-3(3)
B(1)-C(97)-C(98)-C(99)	178.1(16)
C(102)-C(97)-C(98)-F(1)	178.9(14)
B(1)-C(97)-C(98)-F(1)	0(2)
F(1)-C(98)-C(99)-C(100)	178.1(16)
C(97)-C(98)-C(99)-C(100)	0(3)
F(1)-C(98)-C(99)-F(2)	-1(2)
C(97)-C(98)-C(99)-F(2)	-179.6(16)
F(2)-C(99)-C(100)-F(3)	0(3)
C(98)-C(99)-C(100)-F(3)	-179.2(16)
F(2)-C(99)-C(100)-C(101)	-177.2(15)
C(98)-C(99)-C(100)-C(101)	3(3)
F(3)-C(100)-C(101)-F(4)	2(3)
C(99)-C(100)-C(101)-F(4)	179.4(16)
F(3)-C(100)-C(101)-C(102)	179.2(15)
C(99)-C(100)-C(101)-C(102)	-3(3)
F(4)-C(101)-C(102)-F(5)	-4(2)
C(100)-C(101)-C(102)-F(5)	178.7(15)
F(4)-C(101)-C(102)-C(97)	177.3(15)
C(100)-C(101)-C(102)-C(97)	0(3)
C(98)-C(97)-C(102)-F(5)	-175.6(14)
B(1)-C(97)-C(102)-F(5)	3(3)

C(98)-C(97)-C(102)-C(101)	3(2)
B(1)-C(97)-C(102)-C(101)	-178.2(16)
C(108)-C(103)-C(104)-C(105)	-1(2)
B(1)-C(103)-C(104)-C(105)	-174.8(15)
C(108)-C(103)-C(104)-F(6)	178.5(13)
B(1)-C(103)-C(104)-F(6)	4(2)
F(6)-C(104)-C(105)-F(7)	1(2)
C(103)-C(104)-C(105)-F(7)	-180.0(14)
F(6)-C(104)-C(105)-C(106)	-180.0(14)
C(103)-C(104)-C(105)-C(106)	-1(2)
F(7)-C(105)-C(106)-C(107)	-178.5(15)
C(104)-C(105)-C(106)-C(107)	2(3)
F(7)-C(105)-C(106)-F(8)	-1(2)
C(104)-C(105)-C(106)-F(8)	180.0(14)
C(105)-C(106)-C(107)-F(9)	177.7(15)
F(8)-C(106)-C(107)-F(9)	0(2)
C(105)-C(106)-C(107)-C(108)	-2(2)
F(8)-C(106)-C(107)-C(108)	-179.9(13)
C(104)-C(103)-C(108)-F(10)	-179.0(13)
B(1)-C(103)-C(108)-F(10)	-4(2)
C(104)-C(103)-C(108)-C(107)	1(2)
B(1)-C(103)-C(108)-C(107)	175.5(14)
F(9)-C(107)-C(108)-F(10)	0(2)
C(106)-C(107)-C(108)-F(10)	-179.7(14)
F(9)-C(107)-C(108)-C(103)	-179.4(14)
C(106)-C(107)-C(108)-C(103)	1(3)
C(114)-C(109)-C(110)-C(111)	0(2)
B(1)-C(109)-C(110)-C(111)	172.7(15)
C(114)-C(109)-C(110)-F(11)	-179.5(12)
B(1)-C(109)-C(110)-F(11)	-6(2)
F(11)-C(110)-C(111)-F(12)	0(2)
C(109)-C(110)-C(111)-F(12)	-179.3(14)
F(11)-C(110)-C(111)-C(112)	179.4(13)
C(109)-C(110)-C(111)-C(112)	0(2)
F(12)-C(111)-C(112)-F(13)	1(2)
C(110)-C(111)-C(112)-F(13)	-178.6(14)

F(12)-C(111)-C(112)-C(113)	-179.2(14)
C(110)-C(111)-C(112)-C(113)	1(2)
F(13)-C(112)-C(113)-F(14)	1(2)
C(111)-C(112)-C(113)-F(14)	-178.9(14)
F(13)-C(112)-C(113)-C(114)	177.5(14)
C(111)-C(112)-C(113)-C(114)	-2(2)
F(14)-C(113)-C(114)-C(109)	178.8(15)
C(112)-C(113)-C(114)-C(109)	2(3)
F(14)-C(113)-C(114)-F(15)	0(2)
C(112)-C(113)-C(114)-F(15)	-176.8(14)
C(110)-C(109)-C(114)-C(113)	-1(2)
B(1)-C(109)-C(114)-C(113)	-174.7(15)
C(110)-C(109)-C(114)-F(15)	178.3(13)
B(1)-C(109)-C(114)-F(15)	4(2)
C(120)-C(115)-C(116)-F(16)	-176.6(13)
B(1)-C(115)-C(116)-F(16)	-4(2)
C(120)-C(115)-C(116)-C(117)	1(2)
B(1)-C(115)-C(116)-C(117)	174.1(16)
F(16)-C(116)-C(117)-F(17)	-4(2)
C(115)-C(116)-C(117)-F(17)	177.9(15)
F(16)-C(116)-C(117)-C(118)	-179.7(14)
C(115)-C(116)-C(117)-C(118)	3(3)
F(17)-C(117)-C(118)-C(119)	178.7(15)
C(116)-C(117)-C(118)-C(119)	-6(2)
F(17)-C(117)-C(118)-F(18)	4(2)
C(116)-C(117)-C(118)-F(18)	179.0(14)
F(18)-C(118)-C(119)-F(19)	1(3)
C(117)-C(118)-C(119)-F(19)	-174.2(15)
F(18)-C(118)-C(119)-C(120)	-179.4(14)
C(117)-C(118)-C(119)-C(120)	6(3)
C(116)-C(115)-C(120)-F(20)	176.3(14)
B(1)-C(115)-C(120)-F(20)	4(2)
C(116)-C(115)-C(120)-C(119)	-2(2)
B(1)-C(115)-C(120)-C(119)	-174.1(15)
C(118)-C(119)-C(120)-F(20)	-179.8(15)
F(19)-C(119)-C(120)-F(20)	0(2)

C(118)-C(119)-C(120)-C(115)	-2(3)
F(19)-C(119)-C(120)-C(115)	178.0(14)
C(116)-C(115)-B(1)-C(109)	172.4(14)
C(120)-C(115)-B(1)-C(109)	-16(2)
C(116)-C(115)-B(1)-C(97)	54(2)
C(120)-C(115)-B(1)-C(97)	-134.3(16)
C(116)-C(115)-B(1)-C(103)	-67.8(17)
C(120)-C(115)-B(1)-C(103)	104.3(17)
C(114)-C(109)-B(1)-C(115)	-55(2)
C(110)-C(109)-B(1)-C(115)	131.8(16)
C(114)-C(109)-B(1)-C(97)	68.7(17)
C(110)-C(109)-B(1)-C(97)	-104.1(18)
C(114)-C(109)-B(1)-C(103)	-169.1(14)
C(110)-C(109)-B(1)-C(103)	18(2)
C(98)-C(97)-B(1)-C(115)	-168.5(15)
C(102)-C(97)-B(1)-C(115)	13(2)
C(98)-C(97)-B(1)-C(109)	66.5(18)
C(102)-C(97)-B(1)-C(109)	-112.3(18)
C(98)-C(97)-B(1)-C(103)	-55(2)
C(102)-C(97)-B(1)-C(103)	126.7(17)
C(108)-C(103)-B(1)-C(115)	-67.8(16)
C(104)-C(103)-B(1)-C(115)	105.8(17)
C(108)-C(103)-B(1)-C(109)	54.2(19)
C(104)-C(103)-B(1)-C(109)	-132.1(16)
C(108)-C(103)-B(1)-C(97)	170.7(14)
C(104)-C(103)-B(1)-C(97)	-16(2)
C(121)-C(122)-O(20)-C(123)	180.0
C(122)-O(20)-C(123)-C(124)	-180.0
C(125)-C(126)-O(21)-C(127)	180.0
C(126)-O(21)-C(127)-C(128)	180.0
C(129)-C(130)-O(22)-C(131)	-180.0
C(130)-O(22)-C(131)-C(132)	180.0

Symmetry transformations used to generate equivalent atoms:

#1 -x+1,y-1/2,-z+1/2 #2 -x+3/2,-y+1,z+1/2 #3 -x+1,y+1/2,-z+1/2

#4 -x+3/2,-y+1,z-1/2

University of Illinois, Department of Chemistry

George L. Clark X- R Facility and 3M Materials Laboratory

Structure report for compound **PhenylalanineHIM/NaBArF24**

Identification code	2390609
Empirical formula	C128 H84 B F24 I6 N6 Na O20
Principal Investigator:	Professor Dr. Kyle N Plunkett

List of Tables

Table 1. Crystallographic Experimental Details

Table 2. Atomic Coordinates and Equivalent Isotropic Displacement Parameters

Table 3. Selected Interatomic Distances and Selected Interatomic Angles

Table 4. Anisotropic Displacement Parameters

Table 5. Derived Atomic Coordinates and Displacement Parameters for Hydrogen Atoms

Table 6. Torsional Angles

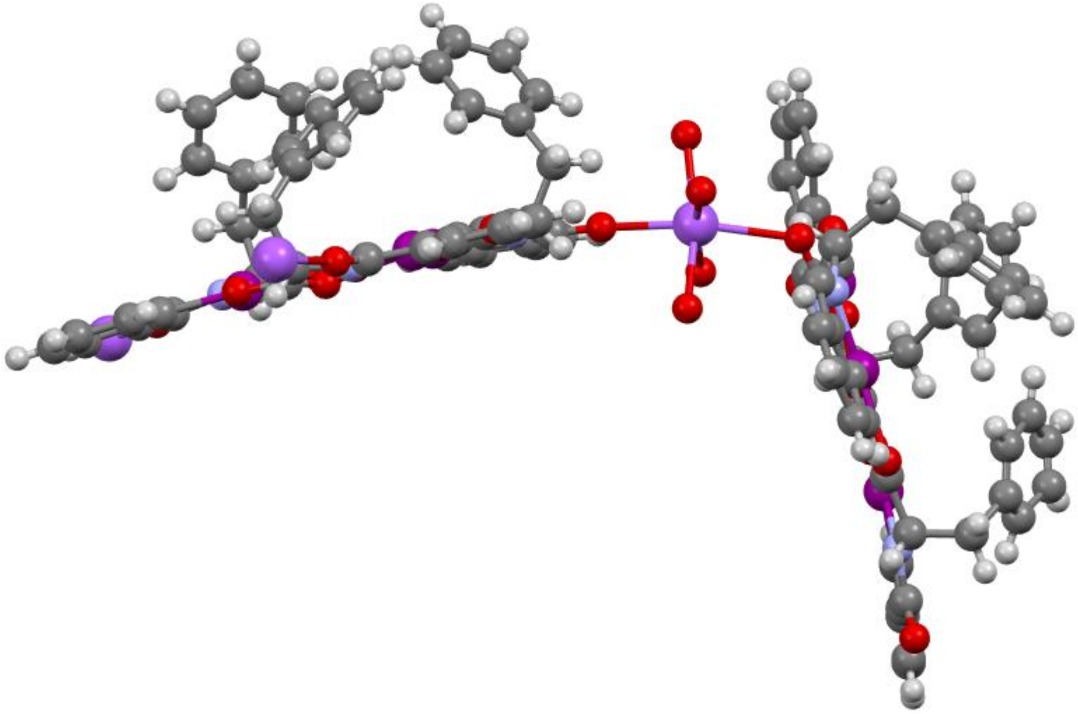

Figure S12. Perspective view of crystallographically independent molecules of phenylalanine HIM **1**/NaBArF24 complex. Solvent is removed for clarity. Nitrogen, oxygen, iodine and sodium atoms are denoted by light blue, red, purple and lavender color respectively. Hydrogens atoms are omitted. All three benzyl groups in both phenylalanine HIM are projected towards the interior of the macrocycle. (Ellipsoid style)

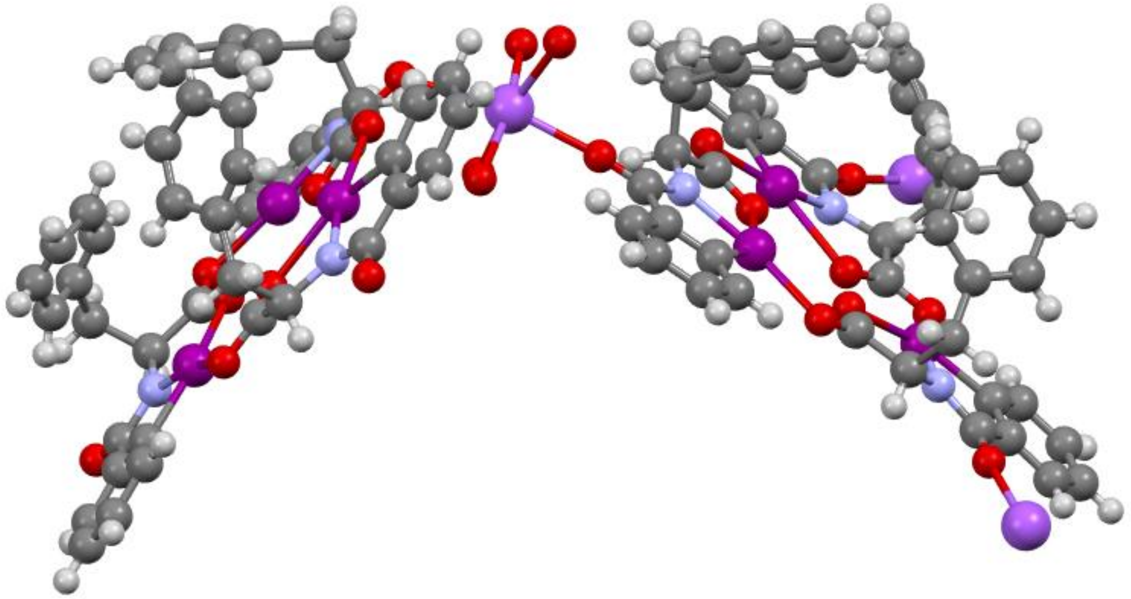

Figure S13. Perspective view of crystallographically independent molecules of phenylalanine HIM **1** /NaBArF$_{24}$ complex. Solvent is removed for clarity. Nitrogen, oxygen, iodine and sodium atoms are denoted by light blue, red, purple and lavender color respectively. Hydrogens atoms are omitted. All three benzyl groups in both phenylalanine HIM are projected towards the interior of the macrocycle. (Ellipsoid style)

Table 1. Crystal data and structure refinement for 16157ds.

Identification code	2390609	
Empirical formula	C128 H84 B F24 I6 N6 Na O20	
Formula weight	3277.21	
Temperature	100(2) K	
Wavelength	0.71073 Å	
Crystal system	Orthorhombic	
Space group	$P2_12_12_1$	
Unit cell dimensions	a = 21.4764(5) Å	a= 90°.
	b = 23.9221(5) Å	b= 90°.
	c = 24.9355(5) Å	g = 90°.
Volume	12810.9(5) $Å^3$	
Z	4	
Density (calculated)	1.699 Mg/m^3	
Absorption coefficient	1.562 mm^{-1}	
F(000)	6416	
Crystal size	0.398 x 0.188 x 0.075 mm^3	
Theta range for data collection	2.072 to 27.533°.	
Index ranges	-27<=h<=27, -30<=k<=31, -32<=l<=32	
Reflections collected	173155	
Independent reflections	29436 [R(int) = 0.0532]	
Completeness to theta = 25.242°	99.9 %	
Absorption correction	Semi-empirical from equivalents	
Max. and min. transmission	0.7456 and 0.6306	
Refinement method	Full-matrix least-squares on F^2	
Data / restraints / parameters	29436 / 4880 / 2157	
Goodness-of-fit on F^2	1.062	
Final R indices [I>2sigma(I)]	R1 = 0.0358, wR2 = 0.0824	
R indices (all data)	R1 = 0.0445, wR2 = 0.0882	
Absolute structure parameter	-0.023(4)	
Extinction coefficient	n/a	
Largest diff. peak and hole	0.867 and -1.090 e.$Å^{-3}$	

Table 2. Atomic coordinates (x 10^4) and equivalent isotropic displacement parameters ($Å^2$x 10^3) for 16157ds. U(eq) is defined as one third of the trace of the orthogonalized U^{ij} tensor.

	x	y	z	U(eq)
I(1)	5532(1)	3851(1)	4008(1)	30(1)
I(2)	6246(1)	5747(1)	4911(1)	30(1)
I(3)	4539(1)	4492(1)	5835(1)	32(1)
I(4)	10430(1)	2298(1)	4218(1)	26(1)
I(5)	12044(1)	691(1)	4587(1)	25(1)
I(6)	9345(1)	5637(1)	2145(1)	26(1)
Na(1)	8020(1)	3430(1)	3093(1)	29(1)
O(1)	4724(2)	3351(2)	4413(2)	35(1)
O(2)	4931(3)	4044(2)	4982(2)	40(1)
O(3)	3370(3)	3118(2)	6057(2)	41(1)
O(4)	4887(3)	5333(2)	6215(2)	38(1)
O(5)	5447(3)	5238(2)	5467(2)	42(1)
O(6)	6547(3)	6831(3)	6185(3)	60(2)
O(7)	6609(2)	5459(2)	4060(2)	35(1)
O(8)	5994(3)	4768(2)	4328(2)	40(1)
O(9)	6922(3)	3782(2)	2837(2)	39(1)
O(10)	10408(2)	1578(2)	3488(2)	34(1)
O(11)	9978(2)	1384(2)	2704(2)	33(1)
O(12)	8919(2)	3099(2)	3502(2)	36(1)
O(13)	11340(2)	1527(2)	4540(2)	34(1)
O(14)	11068(2)	2295(2)	4982(2)	33(1)
O(15)	12668(2)	1234(2)	6078(2)	35(1)
O(16)	12113(2)	-34(2)	3967(2)	30(1)
O(17)	11295(2)	450(2)	3687(2)	36(1)
O(18)	8635(2)	4175(2)	2816(2)	34(1)
O(19)	7603(3)	2523(2)	3296(3)	54(2)
O(20)	7819(7)	3222(7)	2185(4)	71(4)
O(20A)	8179(9)	2980(7)	2259(5)	67(4)
N(1)	4070(3)	3733(3)	5696(2)	34(1)
N(2)	6151(3)	6144(3)	5662(3)	36(1)
N(3)	6261(3)	4097(2)	3498(2)	33(1)

N(4)	9742(3)	2512(2)	3649(2)	28(1)
N(5)	12202(3)	1216(2)	5260(2)	28(1)
N(6)	8836(3)	4883(2)	2224(2)	26(1)
C(1)	4619(4)	3627(3)	4841(3)	34(2)
C(2)	4053(4)	3452(3)	5178(3)	35(2)
C(3)	3449(3)	3586(3)	4868(3)	37(2)
C(4)	3675(3)	3558(3)	6091(3)	34(2)
C(5)	3648(4)	3940(3)	6553(3)	37(2)
C(6)	3981(3)	4442(3)	6528(3)	35(2)
C(7)	3951(4)	4840(4)	6930(3)	40(2)
C(8)	3581(4)	4721(4)	7366(4)	49(2)
C(9)	3251(4)	4221(4)	7401(3)	50(2)
C(10)	3285(4)	3834(4)	6999(3)	41(2)
C(11)	3342(7)	4213(3)	4859(5)	44(3)
C(12)	3624(5)	4541(4)	4468(4)	53(3)
C(13)	3579(5)	5120(3)	4493(4)	60(3)
C(14)	3251(5)	5372(3)	4908(5)	61(3)
C(15)	2968(6)	5044(4)	5299(4)	61(3)
C(16)	3014(7)	4465(4)	5275(5)	50(3)
C(11A)	3337(17)	4196(6)	4745(13)	49(4)
C(12A)	3477(12)	4468(8)	4268(11)	60(4)
C(13A)	3359(12)	5037(8)	4215(10)	62(4)
C(14A)	3100(12)	5333(5)	4639(11)	63(4)
C(15A)	2959(15)	5061(10)	5116(10)	61(4)
C(16A)	3078(19)	4492(10)	5169(11)	54(4)
C(17)	5299(3)	5505(3)	5880(3)	34(2)
C(18)	5603(4)	6063(3)	6008(3)	36(2)
C(19)	6546(4)	6567(3)	5758(4)	43(2)
C(20)	6943(4)	6705(4)	5302(4)	49(2)
C(21)	6888(4)	6402(3)	4832(3)	39(2)
C(22)	7206(4)	6529(4)	4363(4)	48(2)
C(23)	7593(5)	7005(4)	4379(5)	70(3)
C(24)	7658(7)	7314(6)	4840(5)	100(5)
C(25)	7340(6)	7172(5)	5305(5)	80(4)
C(26)	5133(4)	6546(3)	5944(4)	46(2)
C(27)	5014(9)	6695(8)	5358(3)	45(3)

C(28)	5402(8)	7077(9)	5104(5)	52(3)
C(29)	5331(7)	7182(7)	4559(5)	57(3)
C(30)	4872(7)	6906(5)	4269(3)	61(3)
C(31)	4484(6)	6524(4)	4524(4)	57(3)
C(32)	4555(7)	6419(5)	5068(4)	52(3)
C(27A)	4990(20)	6629(19)	5347(6)	48(4)
C(28A)	5290(20)	7048(19)	5063(11)	49(4)
C(29A)	5201(18)	7100(15)	4514(10)	53(4)
C(30A)	4803(17)	6734(11)	4249(5)	58(4)
C(31A)	4499(16)	6316(10)	4533(10)	58(4)
C(32A)	4592(18)	6263(13)	5082(9)	52(4)
C(33)	6362(3)	4979(3)	3993(3)	33(2)
C(34)	6510(3)	4665(3)	3482(3)	32(2)
C(35)	6473(3)	3706(3)	3143(3)	34(2)
C(36)	6095(4)	3187(3)	3151(3)	36(2)
C(37)	5591(4)	3153(3)	3504(3)	35(2)
C(38)	5202(4)	2689(3)	3516(3)	44(2)
C(39)	5316(5)	2264(4)	3156(4)	55(2)
C(40)	5812(5)	2296(4)	2791(4)	54(2)
C(41)	6203(4)	2752(3)	2793(3)	43(2)
C(42)	6277(4)	4988(3)	2987(3)	40(2)
C(43)	5565(3)	4988(8)	2950(7)	65(3)
C(44)	5267(5)	4554(7)	2682(7)	71(3)
C(45)	4622(5)	4515(6)	2692(5)	86(4)
C(46)	4273(3)	4910(7)	2969(5)	94(4)
C(47)	4571(5)	5344(6)	3237(5)	89(4)
C(48)	5216(5)	5383(6)	3228(6)	79(4)
C(43A)	5579(6)	5060(18)	2970(18)	70(4)
C(44A)	5191(12)	4696(15)	2693(16)	75(4)
C(45A)	4570(11)	4834(14)	2610(12)	84(4)
C(46A)	4336(7)	5335(15)	2806(12)	89(5)
C(47A)	4724(12)	5700(13)	3084(11)	86(5)
C(48A)	5345(10)	5562(16)	3166(14)	78(4)
C(49)	10042(3)	1698(3)	3111(3)	28(1)
C(50)	9649(3)	2227(3)	3137(3)	30(1)
C(51)	9364(4)	2946(3)	3780(3)	33(2)

C(52)	9543(4)	3218(3)	4294(3)	31(2)
C(53)	10036(3)	3007(3)	4584(3)	30(2)
C(54)	10238(4)	3250(3)	5058(3)	36(2)
C(55)	9929(4)	3731(3)	5226(3)	42(2)
C(56)	9433(4)	3950(3)	4936(3)	42(2)
C(57)	9239(4)	3692(3)	4480(3)	37(2)
C(58)	9781(4)	2600(3)	2639(3)	37(2)
C(59)	10383(2)	2923(2)	2682(2)	37(2)
C(60)	10961(2)	2665(2)	2638(2)	45(2)
C(61)	11502(2)	2976(2)	2703(3)	51(2)
C(62)	11467(3)	3545(2)	2811(3)	62(3)
C(63)	10889(3)	3803(2)	2855(3)	72(3)
C(64)	10347(2)	3492(2)	2791(2)	54(2)
C(65)	11408(3)	1856(3)	4919(3)	30(1)
C(66)	11927(3)	1768(3)	5321(3)	30(1)
C(67)	12572(3)	1001(3)	5645(3)	28(1)
C(68)	12856(3)	455(3)	5499(3)	28(1)
C(69)	12713(3)	220(3)	5008(3)	29(1)
C(70)	12977(3)	-278(3)	4835(3)	33(2)
C(71)	13399(4)	-538(3)	5174(3)	41(2)
C(72)	13544(4)	-308(3)	5671(3)	38(2)
C(73)	13272(3)	180(3)	5834(3)	33(2)
C(74)	12414(3)	2245(3)	5268(3)	33(2)
C(75)	12697(2)	2288(2)	4711(2)	33(2)
C(76)	13254(2)	2017(2)	4597(2)	55(2)
C(77)	13525(3)	2073(3)	4094(3)	66(3)
C(78)	13238(3)	2400(3)	3704(2)	69(3)
C(79)	12681(3)	2670(3)	3818(2)	61(3)
C(80)	12410(2)	2614(2)	4322(2)	43(2)
C(81)	11689(3)	84(3)	3625(3)	27(1)
C(82)	8281(3)	4759(3)	1906(3)	26(1)
C(83)	8937(3)	4598(3)	2681(3)	26(1)
C(84)	9441(3)	4836(3)	3021(3)	26(1)
C(85)	9702(3)	5343(3)	2877(3)	25(1)
C(86)	10153(3)	5605(3)	3182(3)	35(2)
C(87)	10345(4)	5342(3)	3653(3)	38(2)

C(88)	10074(4)	4833(3)	3807(3)	36(2)
C(89)	9637(3)	4581(3)	3489(3)	32(2)
C(90)	7671(3)	4920(3)	2203(3)	29(1)
C(91)	7686(2)	5509(2)	2421(2)	31(2)
C(92)	7893(2)	5593(2)	2943(2)	32(2)
C(93)	7977(3)	6132(2)	3136(2)	39(2)
C(94)	7855(3)	6589(2)	2807(2)	54(2)
C(95)	7648(3)	6505(2)	2285(2)	58(3)
C(96)	7564(3)	5965(2)	2092(2)	43(2)
F(1)	3165(9)	6524(7)	5664(6)	82(3)
F(2)	3197(9)	5974(6)	6334(6)	85(3)
F(3)	3297(9)	6859(7)	6460(7)	85(3)
F(4)	1543(7)	7512(8)	7398(6)	117(4)
F(5)	721(8)	7021(8)	7377(7)	122(4)
F(6)	840(9)	7705(7)	6812(6)	126(4)
F(7)	2094(5)	4076(4)	4095(4)	50(2)
F(8)	1107(5)	4007(4)	4214(6)	50(2)
F(9)	1721(4)	3914(3)	4886(3)	50(2)
F(10)	2615(4)	5941(4)	3623(3)	51(2)
F(11)	2603(5)	6497(4)	4294(4)	49(2)
F(12)	1865(5)	6528(4)	3720(4)	56(2)
F(13)	1775(7)	3942(6)	6561(5)	102(3)
F(14)	1909(6)	4667(5)	7065(6)	93(4)
F(15)	1335(6)	4004(5)	7315(5)	90(3)
F(16)	-863(5)	4114(4)	6893(4)	67(2)
F(17)	-1072(5)	4311(5)	6065(4)	58(3)
F(18)	-1145(5)	4934(4)	6668(4)	60(2)
F(19)	31(4)	6975(4)	3903(4)	49(2)
F(20)	-821(7)	6486(6)	3894(6)	53(3)
F(21)	-856(4)	7349(3)	4095(4)	54(2)
F(22)	-1054(6)	6825(6)	6557(5)	80(3)
F(23)	-1511(5)	7347(4)	5982(5)	75(3)
F(24)	-1756(5)	6482(4)	6059(5)	75(3)
C(97)	1262(5)	6260(6)	5946(6)	50(2)
C(98)	1903(5)	6185(8)	5919(8)	56(2)
C(99)	2295(4)	6511(9)	6234(8)	66(2)

C(100)	2045(5)	6910(7)	6577(7)	71(2)
C(101)	1404(5)	6985(6)	6604(6)	75(2)
C(102)	1013(4)	6660(6)	6289(6)	61(2)
C(103)	2985(6)	6473(7)	6176(6)	74(2)
C(104)	1128(7)	7294(7)	7066(5)	96(2)
C(105)	1192(4)	5585(3)	5058(3)	35(2)
C(106)	1231(4)	5014(3)	4958(3)	37(2)
C(107)	1596(4)	4818(2)	4538(4)	39(2)
C(108)	1923(4)	5192(3)	4218(3)	35(2)
C(109)	1885(4)	5763(3)	4318(3)	36(2)
C(110)	1519(4)	5959(2)	4738(3)	32(2)
C(111)	1634(5)	4205(4)	4436(4)	44(2)
C(112)	2240(5)	6174(4)	3985(4)	44(2)
C(113)	594(4)	5295(3)	5996(3)	39(2)
C(114)	1080(3)	5038(4)	6269(4)	45(2)
C(115)	958(3)	4598(4)	6618(4)	57(2)
C(116)	350(4)	4416(3)	6694(4)	49(2)
C(117)	-137(3)	4673(4)	6421(4)	43(2)
C(118)	-14(3)	5113(4)	6072(4)	39(2)
C(119)	1487(5)	4303(5)	6882(5)	76(3)
C(120)	-799(5)	4503(4)	6510(4)	48(2)
C(121)	138(4)	6193(4)	5392(3)	38(2)
C(122)	74(4)	6378(4)	4867(3)	38(2)
C(123)	-424(4)	6719(4)	4726(3)	41(2)
C(124)	-858(4)	6875(4)	5112(3)	47(2)
C(125)	-794(4)	6690(4)	5637(3)	51(2)
C(126)	-296(4)	6349(4)	5778(3)	44(2)
C(127)	-509(5)	6885(4)	4151(4)	45(2)
C(128)	-1272(5)	6836(5)	6053(5)	61(2)
B(1)	781(6)	5831(5)	5580(5)	39(1)
F(1A)	3165(11)	6350(8)	5708(7)	79(4)
F(2A)	3202(11)	6065(8)	6523(7)	83(4)
F(3A)	3263(10)	6946(7)	6336(9)	83(4)
F(4A)	1416(8)	7743(7)	7158(8)	117(4)
F(5A)	508(7)	7489(7)	6930(6)	91(3)
F(6A)	1045(10)	7006(9)	7501(7)	121(4)

F(7A)	2094(5)	6026(4)	3658(4)	53(2)
F(8A)	1935(6)	6878(5)	3439(4)	63(3)
F(9A)	2440(6)	6674(6)	4159(5)	57(3)
F(10A)	-285(5)	7218(5)	3606(4)	57(2)
F(11A)	-692(8)	6428(6)	3786(8)	53(4)
F(12A)	-705(6)	7098(5)	4366(5)	63(2)
F(13A)	1598(5)	4546(4)	3908(4)	49(2)
F(14A)	1098(7)	3820(5)	4148(7)	50(3)
F(15A)	2086(6)	3862(5)	4248(6)	58(3)
F(16A)	1986(8)	4240(7)	6619(7)	103(4)
F(17A)	1083(6)	3888(7)	6583(6)	91(3)
F(18A)	1829(9)	3461(6)	6202(7)	112(4)
F(19A)	-1207(6)	7039(5)	5832(5)	66(2)
F(20A)	-1720(5)	6637(6)	6465(6)	74(3)
F(21A)	-842(7)	7053(6)	6597(6)	71(3)
F(22A)	-515(5)	4243(4)	6441(4)	52(2)
F(23A)	-1341(6)	4421(6)	5997(5)	51(3)
F(24A)	-1283(5)	4631(5)	6828(4)	46(2)
C(97A)	1264(6)	6188(7)	5962(7)	50(2)
C(98A)	1906(6)	6183(10)	5898(9)	56(2)
C(99A)	2281(5)	6500(11)	6235(10)	66(2)
C(200)	2013(6)	6823(9)	6637(8)	71(2)
C(201)	1370(6)	6829(8)	6701(7)	76(2)
C(202)	995(5)	6511(7)	6364(6)	62(2)
C(203)	2974(6)	6467(7)	6207(7)	74(2)
C(204)	1081(7)	7287(6)	7031(7)	95(2)
C(205)	797(4)	6149(4)	4925(3)	36(2)
C(206)	1354(3)	6234(4)	4653(4)	36(2)
C(207)	1358(3)	6517(4)	4166(4)	37(2)
C(208)	803(4)	6715(4)	3951(3)	37(2)
C(209)	246(3)	6631(5)	4222(4)	40(2)
C(210)	243(3)	6348(5)	4709(4)	37(2)
C(211)	1952(5)	6532(5)	3857(5)	47(2)
C(212)	-356(5)	6842(5)	3997(5)	43(2)
C(213)	1130(5)	5159(3)	5418(4)	39(2)
C(214)	1224(6)	4946(4)	4906(3)	37(2)

C(215)	1448(6)	4405(4)	4840(3)	41(2)
C(216)	1579(6)	4077(3)	5287(4)	48(3)
C(217)	1485(6)	4290(4)	5799(3)	55(3)
C(218)	1261(6)	4831(4)	5865(3)	43(2)
C(219)	1562(6)	4163(5)	4296(4)	44(2)
C(220)	1596(7)	3962(6)	6295(5)	76(3)
C(221)	91(4)	5738(4)	5786(4)	39(2)
C(222)	-253(5)	6219(3)	5886(5)	43(2)
C(223)	-822(5)	6182(3)	6150(5)	51(2)
C(224)	-1047(4)	5665(4)	6312(5)	45(2)
C(225)	-704(5)	5184(3)	6211(5)	42(2)
C(226)	-135(4)	5220(3)	5948(5)	38(2)
C(227)	-1155(6)	6713(5)	6263(6)	61(2)
C(228)	-962(5)	4629(4)	6372(4)	46(2)
B(1A)	816(6)	5807(6)	5518(5)	40(2)

Table 3. Bond lengths [Å] and angles [°] for 16157ds.

I(1)-C(37)	2.093(7)
I(1)-N(3)	2.101(6)
I(1)-O(1)	2.336(5)
I(2)-C(21)	2.097(7)
I(2)-N(2)	2.107(6)
I(2)-O(7)	2.366(5)
I(3)-N(1)	2.105(6)
I(3)-C(6)	2.107(7)
I(3)-O(4)	2.347(5)
I(4)-C(53)	2.102(6)
I(4)-N(4)	2.111(6)
I(4)-O(14)	2.346(5)
I(5)-C(69)	2.106(6)
I(5)-N(5)	2.123(5)
I(5)-O(16)	2.327(4)
I(6)-C(85)	2.100(6)
I(6)-N(6)	2.120(5)
I(6)-O(11)#1	2.333(5)
Na(1)-O(12)	2.324(6)
Na(1)-O(18)	2.324(5)
Na(1)-O(15)#2	2.343(5)
Na(1)-O(20)	2.357(10)
Na(1)-O(20A)	2.365(12)
Na(1)-O(19)	2.401(6)
Na(1)-O(9)	2.584(6)
O(1)-C(1)	1.274(9)
O(2)-C(1)	1.253(9)
O(3)-C(4)	1.243(9)
O(4)-C(17)	1.284(9)
O(5)-C(17)	1.254(9)
O(6)-C(19)	1.236(10)
O(7)-C(33)	1.277(9)
O(8)-C(33)	1.256(9)
O(9)-C(35)	1.245(9)

O(10)-C(49)	1.260(8)
O(11)-C(49)	1.270(8)
O(12)-C(51)	1.236(9)
O(13)-C(65)	1.239(8)
O(14)-C(65)	1.289(8)
O(15)-C(67)	1.234(8)
O(16)-C(81)	1.278(8)
O(17)-C(81)	1.230(8)
O(18)-C(83)	1.248(8)
N(1)-C(4)	1.365(9)
N(1)-C(2)	1.457(9)
N(2)-C(19)	1.343(10)
N(2)-C(18)	1.471(10)
N(3)-C(35)	1.366(10)
N(3)-C(34)	1.461(9)
N(4)-C(51)	1.357(8)
N(4)-C(50)	1.463(8)
N(5)-C(67)	1.348(9)
N(5)-C(66)	1.454(9)
N(6)-C(83)	1.345(8)
N(6)-C(82)	1.462(8)
C(1)-C(2)	1.535(10)
C(2)-C(3)	1.544(11)
C(2)-H(2)	1.0000
C(3)-C(11A)	1.510(13)
C(3)-C(11)	1.519(10)
C(3)-H(3A)	0.9900
C(3)-H(3B)	0.9900
C(3)-H(3C)	0.9900
C(3)-H(3D)	0.9900
C(4)-C(5)	1.472(11)
C(5)-C(10)	1.382(10)
C(5)-C(6)	1.400(11)
C(6)-C(7)	1.384(11)
C(7)-C(8)	1.376(12)
C(7)-H(7)	0.9500

C(8)-C(9)	1.392(13)
C(8)-H(8)	0.9500
C(9)-C(10)	1.366(12)
C(9)-H(9)	0.9500
C(10)-H(10)	0.9500
C(11)-C(12)	1.3900
C(11)-C(16)	1.3900
C(12)-C(13)	1.3900
C(12)-H(12)	0.9500
C(13)-C(14)	1.3900
C(13)-H(13)	0.9500
C(14)-C(15)	1.3900
C(14)-H(14)	0.9500
C(15)-C(16)	1.3900
C(15)-H(15)	0.9500
C(16)-H(16)	0.9500
C(11A)-C(12A)	1.3900
C(11A)-C(16A)	1.3900
C(12A)-C(13A)	1.3900
C(12A)-H(12A)	0.9500
C(13A)-C(14A)	1.3900
C(13A)-H(13A)	0.9500
C(14A)-C(15A)	1.3900
C(14A)-H(14A)	0.9500
C(15A)-C(16A)	1.3900
C(15A)-H(15A)	0.9500
C(16A)-H(16A)	0.9500
C(17)-C(18)	1.519(10)
C(18)-C(26)	1.543(10)
C(18)-H(18)	1.0000
C(19)-C(20)	1.461(12)
C(20)-C(21)	1.383(12)
C(20)-C(25)	1.404(12)
C(21)-C(22)	1.386(12)
C(22)-C(23)	1.409(12)
C(22)-H(22)	0.9500

C(23)-C(24)	1.374(16)
C(23)-H(23)	0.9500
C(24)-C(25)	1.387(16)
C(24)-H(24)	0.9500
C(25)-H(25)	0.9500
C(26)-C(27)	1.525(11)
C(26)-C(27A)	1.533(14)
C(26)-H(26A)	0.9900
C(26)-H(26B)	0.9900
C(26)-H(26C)	0.9900
C(26)-H(26D)	0.9900
C(27)-C(28)	1.3900
C(27)-C(32)	1.3900
C(28)-C(29)	1.3900
C(28)-H(28)	0.9500
C(29)-C(30)	1.3900
C(29)-H(29)	0.9500
C(30)-C(31)	1.3900
C(30)-H(30)	0.9500
C(31)-C(32)	1.3900
C(31)-H(31)	0.9500
C(32)-H(32)	0.9500
C(27A)-C(28A)	1.3900
C(27A)-C(32A)	1.3900
C(28A)-C(29A)	1.3900
C(28A)-H(28A)	0.9500
C(29A)-C(30A)	1.3900
C(29A)-H(29A)	0.9500
C(30A)-C(31A)	1.3900
C(30A)-H(30A)	0.9500
C(31A)-C(32A)	1.3900
C(31A)-H(31A)	0.9500
C(32A)-H(32A)	0.9500
C(33)-C(34)	1.511(10)
C(34)-C(42)	1.540(10)
C(34)-H(34)	1.0000

C(35)-C(36)	1.483(11)
C(36)-C(41)	1.392(11)
C(36)-C(37)	1.398(11)
C(37)-C(38)	1.390(11)
C(38)-C(39)	1.377(12)
C(38)-H(38)	0.9500
C(39)-C(40)	1.403(13)
C(39)-H(39)	0.9500
C(40)-C(41)	1.376(12)
C(40)-H(40)	0.9500
C(41)-H(41)	0.9500
C(42)-C(43A)	1.510(14)
C(42)-C(43)	1.533(10)
C(42)-H(42A)	0.9900
C(42)-H(42B)	0.9900
C(42)-H(42C)	0.9900
C(42)-H(42D)	0.9900
C(43)-C(44)	1.3900
C(43)-C(48)	1.3900
C(44)-C(45)	1.3900
C(44)-H(44)	0.9500
C(45)-C(46)	1.3900
C(45)-H(45)	0.9500
C(46)-C(47)	1.3900
C(46)-H(46)	0.9500
C(47)-C(48)	1.3900
C(47)-H(47)	0.9500
C(48)-H(48)	0.9500
C(43A)-C(44A)	1.3900
C(43A)-C(48A)	1.3900
C(44A)-C(45A)	1.3900
C(44A)-H(44A)	0.9500
C(45A)-C(46A)	1.3900
C(45A)-H(45A)	0.9500
C(46A)-C(47A)	1.3900
C(46A)-H(46A)	0.9500

C(47A)-C(48A)	1.3900
C(47A)-H(47A)	0.9500
C(48A)-H(48A)	0.9500
C(49)-C(50)	1.521(9)
C(50)-C(58)	1.554(10)
C(50)-H(50)	1.0000
C(51)-C(52)	1.490(10)
C(52)-C(53)	1.379(10)
C(52)-C(57)	1.389(9)
C(53)-C(54)	1.387(10)
C(54)-C(55)	1.393(10)
C(54)-H(54)	0.9500
C(55)-C(56)	1.391(12)
C(55)-H(55)	0.9500
C(56)-C(57)	1.358(11)
C(56)-H(56)	0.9500
C(57)-H(57)	0.9500
C(58)-C(59)	1.509(9)
C(58)-H(58A)	0.9900
C(58)-H(58B)	0.9900
C(59)-C(60)	1.3900
C(59)-C(64)	1.3900
C(60)-C(61)	1.3900
C(60)-H(60)	0.9500
C(61)-C(62)	1.3900
C(61)-H(61)	0.9500
C(62)-C(63)	1.3900
C(62)-H(62)	0.9500
C(63)-C(64)	1.3900
C(63)-H(63)	0.9500
C(64)-H(64)	0.9500
C(65)-C(66)	1.514(10)
C(66)-C(74)	1.553(9)
C(66)-H(66)	1.0000
C(67)-C(68)	1.488(10)
C(68)-C(69)	1.382(10)

C(68)-C(73)	1.388(10)
C(69)-C(70)	1.389(10)
C(70)-C(71)	1.386(10)
C(70)-H(70)	0.9500
C(71)-C(72)	1.392(11)
C(71)-H(71)	0.9500
C(72)-C(73)	1.367(11)
C(72)-H(72)	0.9500
C(73)-H(73)	0.9500
C(74)-C(75)	1.520(8)
C(74)-H(74A)	0.9900
C(74)-H(74B)	0.9900
C(75)-C(76)	1.3900
C(75)-C(80)	1.3900
C(76)-C(77)	1.3900
C(76)-H(76)	0.9500
C(77)-C(78)	1.3900
C(77)-H(77)	0.9500
C(78)-C(79)	1.3900
C(78)-H(78)	0.9500
C(79)-C(80)	1.3900
C(79)-H(79)	0.9500
C(80)-H(80)	0.9500
C(81)-C(82)#3	1.537(9)
C(82)-C(90)	1.552(9)
C(82)-H(82)	1.0000
C(83)-C(84)	1.488(9)
C(84)-C(89)	1.383(9)
C(84)-C(85)	1.383(9)
C(85)-C(86)	1.381(9)
C(86)-C(87)	1.395(10)
C(86)-H(86)	0.9500
C(87)-C(88)	1.402(11)
C(87)-H(87)	0.9500
C(88)-C(89)	1.369(11)
C(88)-H(88)	0.9500

C(89)-H(89)	0.9500
C(90)-C(91)	1.510(8)
C(90)-H(90A)	0.9900
C(90)-H(90B)	0.9900
C(91)-C(92)	1.3900
C(91)-C(96)	1.3900
C(92)-C(93)	1.3900
C(92)-H(92)	0.9500
C(93)-C(94)	1.3900
C(93)-H(93)	0.9500
C(94)-C(95)	1.3900
C(94)-H(94)	0.9500
C(95)-C(96)	1.3900
C(95)-H(95)	0.9500
C(96)-H(96)	0.9500
F(1)-C(103)	1.340(9)
F(2)-C(103)	1.336(9)
F(3)-C(103)	1.343(9)
F(4)-C(104)	1.324(9)
F(5)-C(104)	1.339(10)
F(6)-C(104)	1.323(10)
F(7)-C(111)	1.339(9)
F(8)-C(111)	1.346(9)
F(9)-C(111)	1.334(9)
F(10)-C(112)	1.333(9)
F(11)-C(112)	1.339(9)
F(12)-C(112)	1.343(9)
F(13)-C(119)	1.331(9)
F(14)-C(119)	1.335(9)
F(15)-C(119)	1.335(9)
F(16)-C(120)	1.340(9)
F(17)-C(120)	1.337(9)
F(18)-C(120)	1.330(9)
F(19)-C(127)	1.332(9)
F(20)-C(127)	1.331(9)
F(21)-C(127)	1.345(9)

F(22)-C(128)	1.341(9)
F(23)-C(128)	1.338(9)
F(24)-C(128)	1.340(9)
C(97)-C(98)	1.3900
C(97)-C(102)	1.3900
C(97)-B(1)	1.719(11)
C(98)-C(99)	1.3900
C(98)-H(98)	0.9500
C(99)-C(100)	1.3900
C(99)-C(103)	1.493(9)
C(100)-C(101)	1.3900
C(100)-H(100)	0.9500
C(101)-C(102)	1.3900
C(101)-C(104)	1.491(10)
C(102)-H(102)	0.9500
C(105)-C(106)	1.3900
C(105)-C(110)	1.3900
C(105)-B(1)	1.680(13)
C(106)-C(107)	1.3900
C(106)-H(106)	0.9500
C(107)-C(108)	1.3900
C(107)-C(111)	1.491(9)
C(108)-C(109)	1.3900
C(108)-H(108)	0.9500
C(109)-C(110)	1.3900
C(109)-C(112)	1.496(9)
C(110)-H(110)	0.9500
C(113)-C(114)	1.3900
C(113)-C(118)	1.3900
C(113)-B(1)	1.697(13)
C(114)-C(115)	1.3900
C(114)-H(114)	0.9500
C(115)-C(116)	1.3900
C(115)-C(119)	1.492(9)
C(116)-C(117)	1.3900
C(116)-H(116)	0.9500

C(117)-C(118)	1.3900
C(117)-C(120)	1.496(9)
C(118)-H(118)	0.9500
C(121)-C(122)	1.3900
C(121)-C(126)	1.3900
C(121)-B(1)	1.696(13)
C(122)-C(123)	1.3900
C(122)-H(122)	0.9500
C(123)-C(124)	1.3900
C(123)-C(127)	1.499(9)
C(124)-C(125)	1.3900
C(124)-H(124)	0.9500
C(125)-C(126)	1.3900
C(125)-C(128)	1.500(9)
C(126)-H(126)	0.9500
F(1A)-C(203)	1.339(10)
F(2A)-C(203)	1.338(10)
F(3A)-C(203)	1.343(9)
F(4A)-C(204)	1.343(10)
F(5A)-C(204)	1.345(10)
F(6A)-C(204)	1.353(10)
F(7A)-C(211)	1.342(10)
F(8A)-C(211)	1.332(9)
F(9A)-C(211)	1.336(10)
F(10A)-C(212)	1.335(9)
F(11A)-C(212)	1.333(9)
F(12A)-C(212)	1.336(9)
F(13A)-C(219)	1.334(9)
F(14A)-C(219)	1.345(9)
F(15A)-C(219)	1.341(9)
F(16A)-C(220)	1.340(10)
F(17A)-C(220)	1.328(10)
F(18A)-C(220)	1.321(9)
F(19A)-C(227)	1.334(10)
F(20A)-C(227)	1.325(9)
F(21A)-C(227)	1.344(10)

F(22A)-C(228)	1.342(9)
F(23A)-C(228)	1.334(9)
F(24A)-C(228)	1.331(9)
C(97A)-C(98A)	1.3900
C(97A)-C(202)	1.3900
C(97A)-B(1A)	1.728(12)
C(98A)-C(99A)	1.3900
C(98A)-H(98A)	0.9500
C(99A)-C(200)	1.3900
C(99A)-C(203)	1.492(10)
C(200)-C(201)	1.3900
C(200)-H(200)	0.9500
C(201)-C(202)	1.3900
C(201)-C(204)	1.506(10)
C(202)-H(202)	0.9500
C(205)-C(206)	1.3900
C(205)-C(210)	1.3900
C(205)-B(1A)	1.691(14)
C(206)-C(207)	1.3900
C(206)-H(206)	0.9500
C(207)-C(208)	1.3900
C(207)-C(211)	1.493(10)
C(208)-C(209)	1.3900
C(208)-H(208)	0.9500
C(209)-C(210)	1.3900
C(209)-C(212)	1.498(9)
C(210)-H(210)	0.9500
C(213)-C(214)	1.3900
C(213)-C(218)	1.3900
C(213)-B(1A)	1.709(14)
C(214)-C(215)	1.3900
C(214)-H(214)	0.9500
C(215)-C(216)	1.3900
C(215)-C(219)	1.495(9)
C(216)-C(217)	1.3900
C(216)-H(216)	0.9500

C(217)-C(218)	1.3900
C(217)-C(220)	1.483(10)
C(218)-H(218)	0.9500
C(221)-C(222)	1.3900
C(221)-C(226)	1.3900
C(221)-B(1A)	1.703(14)
C(222)-C(223)	1.3900
C(222)-H(222)	0.9500
C(223)-C(224)	1.3900
C(223)-C(227)	1.484(9)
C(224)-C(225)	1.3900
C(224)-H(224)	0.9500
C(225)-C(226)	1.3900
C(225)-C(228)	1.494(9)
C(226)-H(226)	0.9500

C(37)-I(1)-N(3)	79.3(3)
C(37)-I(1)-O(1)	84.1(3)
N(3)-I(1)-O(1)	163.4(2)
C(21)-I(2)-N(2)	79.1(3)
C(21)-I(2)-O(7)	85.2(3)
N(2)-I(2)-O(7)	163.8(2)
N(1)-I(3)-C(6)	79.3(3)
N(1)-I(3)-O(4)	163.4(2)
C(6)-I(3)-O(4)	84.1(3)
C(53)-I(4)-N(4)	79.4(3)
C(53)-I(4)-O(14)	83.4(2)
N(4)-I(4)-O(14)	162.75(19)
C(69)-I(5)-N(5)	79.3(2)
C(69)-I(5)-O(16)	83.6(2)
N(5)-I(5)-O(16)	162.80(19)
C(85)-I(6)-N(6)	79.8(2)
C(85)-I(6)-O(11)#1	83.6(2)
N(6)-I(6)-O(11)#1	163.37(19)
O(12)-Na(1)-O(18)	85.4(2)
O(12)-Na(1)-O(15)#2	89.8(2)

O(18)-Na(1)-O(15)#2	100.5(2)
O(12)-Na(1)-O(20)	120.1(4)
O(18)-Na(1)-O(20)	88.9(4)
O(15)#2-Na(1)-O(20)	149.4(4)
O(12)-Na(1)-O(20A)	96.4(5)
O(18)-Na(1)-O(20A)	90.3(4)
O(15)#2-Na(1)-O(20A)	167.9(5)
O(12)-Na(1)-O(19)	84.8(2)
O(18)-Na(1)-O(19)	165.4(3)
O(15)#2-Na(1)-O(19)	90.2(2)
O(20)-Na(1)-O(19)	86.8(5)
O(20A)-Na(1)-O(19)	80.1(5)
O(12)-Na(1)-O(9)	168.0(2)
O(18)-Na(1)-O(9)	101.26(19)
O(15)#2-Na(1)-O(9)	79.17(19)
O(20)-Na(1)-O(9)	70.4(4)
O(20A)-Na(1)-O(9)	93.6(5)
O(19)-Na(1)-O(9)	90.4(2)
C(1)-O(1)-I(1)	103.3(4)
C(17)-O(4)-I(3)	103.4(4)
C(33)-O(7)-I(2)	104.0(4)
C(35)-O(9)-Na(1)	120.6(5)
C(49)-O(11)-I(6)#3	104.9(4)
C(51)-O(12)-Na(1)	171.9(5)
C(65)-O(14)-I(4)	103.5(4)
C(67)-O(15)-Na(1)#4	169.1(5)
C(81)-O(16)-I(5)	103.6(4)
C(83)-O(18)-Na(1)	175.9(5)
C(4)-N(1)-C(2)	119.0(6)
C(4)-N(1)-I(3)	116.3(5)
C(2)-N(1)-I(3)	123.7(4)
C(19)-N(2)-C(18)	119.8(7)
C(19)-N(2)-I(2)	116.0(6)
C(18)-N(2)-I(2)	122.7(5)
C(35)-N(3)-C(34)	119.8(6)
C(35)-N(3)-I(1)	116.6(5)

C(34)-N(3)-I(1)	123.4(5)
C(51)-N(4)-C(50)	119.0(6)
C(51)-N(4)-I(4)	116.3(5)
C(50)-N(4)-I(4)	124.7(4)
C(67)-N(5)-C(66)	120.7(6)
C(67)-N(5)-I(5)	115.5(5)
C(66)-N(5)-I(5)	123.7(4)
C(83)-N(6)-C(82)	119.3(5)
C(83)-N(6)-I(6)	115.2(4)
C(82)-N(6)-I(6)	122.8(4)
O(2)-C(1)-O(1)	123.6(7)
O(2)-C(1)-C(2)	119.1(7)
O(1)-C(1)-C(2)	117.3(6)
N(1)-C(2)-C(1)	110.0(6)
N(1)-C(2)-C(3)	111.7(6)
C(1)-C(2)-C(3)	109.6(6)
N(1)-C(2)-H(2)	108.5
C(1)-C(2)-H(2)	108.5
C(3)-C(2)-H(2)	108.5
C(11A)-C(3)-C(2)	115.8(14)
C(11)-C(3)-C(2)	109.8(8)
C(11)-C(3)-H(3A)	109.7
C(2)-C(3)-H(3A)	109.7
C(11)-C(3)-H(3B)	109.7
C(2)-C(3)-H(3B)	109.7
H(3A)-C(3)-H(3B)	108.2
C(11A)-C(3)-H(3C)	108.3
C(2)-C(3)-H(3C)	108.3
C(11A)-C(3)-H(3D)	108.3
C(2)-C(3)-H(3D)	108.3
H(3C)-C(3)-H(3D)	107.4
O(3)-C(4)-N(1)	122.6(7)
O(3)-C(4)-C(5)	124.0(7)
N(1)-C(4)-C(5)	113.5(6)
C(10)-C(5)-C(6)	118.7(7)
C(10)-C(5)-C(4)	122.6(7)

C(6)-C(5)-C(4)	118.6(7)
C(7)-C(6)-C(5)	122.3(7)
C(7)-C(6)-I(3)	125.6(6)
C(5)-C(6)-I(3)	112.0(6)
C(8)-C(7)-C(6)	117.1(8)
C(8)-C(7)-H(7)	121.4
C(6)-C(7)-H(7)	121.4
C(7)-C(8)-C(9)	121.5(9)
C(7)-C(8)-H(8)	119.3
C(9)-C(8)-H(8)	119.3
C(10)-C(9)-C(8)	120.5(8)
C(10)-C(9)-H(9)	119.7
C(8)-C(9)-H(9)	119.7
C(9)-C(10)-C(5)	119.8(8)
C(9)-C(10)-H(10)	120.1
C(5)-C(10)-H(10)	120.1
C(12)-C(11)-C(16)	120.0
C(12)-C(11)-C(3)	120.1(8)
C(16)-C(11)-C(3)	119.6(8)
C(13)-C(12)-C(11)	120.0
C(13)-C(12)-H(12)	120.0
C(11)-C(12)-H(12)	120.0
C(14)-C(13)-C(12)	120.0
C(14)-C(13)-H(13)	120.0
C(12)-C(13)-H(13)	120.0
C(13)-C(14)-C(15)	120.0
C(13)-C(14)-H(14)	120.0
C(15)-C(14)-H(14)	120.0
C(16)-C(15)-C(14)	120.0
C(16)-C(15)-H(15)	120.0
C(14)-C(15)-H(15)	120.0
C(15)-C(16)-C(11)	120.0
C(15)-C(16)-H(16)	120.0
C(11)-C(16)-H(16)	120.0
C(12A)-C(11A)-C(16A)	120.0
C(12A)-C(11A)-C(3)	126(2)

C(16A)-C(11A)-C(3)	114(2)
C(13A)-C(12A)-C(11A)	120.0
C(13A)-C(12A)-H(12A)	120.0
C(11A)-C(12A)-H(12A)	120.0
C(12A)-C(13A)-C(14A)	120.0
C(12A)-C(13A)-H(13A)	120.0
C(14A)-C(13A)-H(13A)	120.0
C(15A)-C(14A)-C(13A)	120.0
C(15A)-C(14A)-H(14A)	120.0
C(13A)-C(14A)-H(14A)	120.0
C(14A)-C(15A)-C(16A)	120.0
C(14A)-C(15A)-H(15A)	120.0
C(16A)-C(15A)-H(15A)	120.0
C(15A)-C(16A)-C(11A)	120.0
C(15A)-C(16A)-H(16A)	120.0
C(11A)-C(16A)-H(16A)	120.0
O(5)-C(17)-O(4)	123.1(7)
O(5)-C(17)-C(18)	120.7(7)
O(4)-C(17)-C(18)	116.2(6)
N(2)-C(18)-C(17)	109.6(6)
N(2)-C(18)-C(26)	111.4(6)
C(17)-C(18)-C(26)	110.8(6)
N(2)-C(18)-H(18)	108.3
C(17)-C(18)-H(18)	108.3
C(26)-C(18)-H(18)	108.3
O(6)-C(19)-N(2)	122.7(8)
O(6)-C(19)-C(20)	123.6(7)
N(2)-C(19)-C(20)	113.6(7)
C(21)-C(20)-C(25)	118.2(9)
C(21)-C(20)-C(19)	119.5(7)
C(25)-C(20)-C(19)	122.1(9)
C(20)-C(21)-C(22)	123.9(7)
C(20)-C(21)-I(2)	111.6(6)
C(22)-C(21)-I(2)	124.5(6)
C(21)-C(22)-C(23)	116.3(9)
C(21)-C(22)-H(22)	121.8

C(23)-C(22)-H(22)	121.8
C(24)-C(23)-C(22)	121.2(9)
C(24)-C(23)-H(23)	119.4
C(22)-C(23)-H(23)	119.4
C(23)-C(24)-C(25)	121.1(9)
C(23)-C(24)-H(24)	119.4
C(25)-C(24)-H(24)	119.4
C(24)-C(25)-C(20)	119.3(10)
C(24)-C(25)-H(25)	120.4
C(20)-C(25)-H(25)	120.4
C(27)-C(26)-C(18)	112.5(9)
C(27A)-C(26)-C(18)	109.2(18)
C(27)-C(26)-H(26A)	109.1
C(18)-C(26)-H(26A)	109.1
C(27)-C(26)-H(26B)	109.1
C(18)-C(26)-H(26B)	109.1
H(26A)-C(26)-H(26B)	107.8
C(27A)-C(26)-H(26C)	109.8
C(18)-C(26)-H(26C)	109.8
C(27A)-C(26)-H(26D)	109.8
C(18)-C(26)-H(26D)	109.8
H(26C)-C(26)-H(26D)	108.3
C(28)-C(27)-C(32)	120.0
C(28)-C(27)-C(26)	119.3(12)
C(32)-C(27)-C(26)	120.5(12)
C(29)-C(28)-C(27)	120.0
C(29)-C(28)-H(28)	120.0
C(27)-C(28)-H(28)	120.0
C(30)-C(29)-C(28)	120.0
C(30)-C(29)-H(29)	120.0
C(28)-C(29)-H(29)	120.0
C(29)-C(30)-C(31)	120.0
C(29)-C(30)-H(30)	120.0
C(31)-C(30)-H(30)	120.0
C(30)-C(31)-C(32)	120.0
C(30)-C(31)-H(31)	120.0

C(32)-C(31)-H(31)	120.0
C(31)-C(32)-C(27)	120.0
C(31)-C(32)-H(32)	120.0
C(27)-C(32)-H(32)	120.0
C(28A)-C(27A)-C(32A)	120.0
C(28A)-C(27A)-C(26)	120(3)
C(32A)-C(27A)-C(26)	120(3)
C(29A)-C(28A)-C(27A)	120.0
C(29A)-C(28A)-H(28A)	120.0
C(27A)-C(28A)-H(28A)	120.0
C(28A)-C(29A)-C(30A)	120.0
C(28A)-C(29A)-H(29A)	120.0
C(30A)-C(29A)-H(29A)	120.0
C(29A)-C(30A)-C(31A)	120.0
C(29A)-C(30A)-H(30A)	120.0
C(31A)-C(30A)-H(30A)	120.0
C(32A)-C(31A)-C(30A)	120.0
C(32A)-C(31A)-H(31A)	120.0
C(30A)-C(31A)-H(31A)	120.0
C(31A)-C(32A)-C(27A)	120.0
C(31A)-C(32A)-H(32A)	120.0
C(27A)-C(32A)-H(32A)	120.0
O(8)-C(33)-O(7)	122.3(7)
O(8)-C(33)-C(34)	119.6(6)
O(7)-C(33)-C(34)	118.0(6)
N(3)-C(34)-C(33)	111.2(6)
N(3)-C(34)-C(42)	111.7(6)
C(33)-C(34)-C(42)	111.0(6)
N(3)-C(34)-H(34)	107.6
C(33)-C(34)-H(34)	107.6
C(42)-C(34)-H(34)	107.6
O(9)-C(35)-N(3)	123.8(7)
O(9)-C(35)-C(36)	123.7(7)
N(3)-C(35)-C(36)	112.5(6)
C(41)-C(36)-C(37)	119.4(7)
C(41)-C(36)-C(35)	121.8(7)

C(37)-C(36)-C(35)	118.6(7)
C(38)-C(37)-C(36)	121.6(7)
C(38)-C(37)-I(1)	126.0(6)
C(36)-C(37)-I(1)	112.3(5)
C(39)-C(38)-C(37)	118.0(8)
C(39)-C(38)-H(38)	121.0
C(37)-C(38)-H(38)	121.0
C(38)-C(39)-C(40)	121.2(9)
C(38)-C(39)-H(39)	119.4
C(40)-C(39)-H(39)	119.4
C(41)-C(40)-C(39)	120.3(8)
C(41)-C(40)-H(40)	119.9
C(39)-C(40)-H(40)	119.9
C(40)-C(41)-C(36)	119.5(8)
C(40)-C(41)-H(41)	120.2
C(36)-C(41)-H(41)	120.2
C(43A)-C(42)-C(34)	113.7(19)
C(43)-C(42)-C(34)	111.9(9)
C(43)-C(42)-H(42A)	109.2
C(34)-C(42)-H(42A)	109.2
C(43)-C(42)-H(42B)	109.2
C(34)-C(42)-H(42B)	109.2
H(42A)-C(42)-H(42B)	107.9
C(43A)-C(42)-H(42C)	108.8
C(34)-C(42)-H(42C)	108.8
C(43A)-C(42)-H(42D)	108.8
C(34)-C(42)-H(42D)	108.8
H(42C)-C(42)-H(42D)	107.7
C(44)-C(43)-C(48)	120.0
C(44)-C(43)-C(42)	119.1(11)
C(48)-C(43)-C(42)	120.5(11)
C(43)-C(44)-C(45)	120.0
C(43)-C(44)-H(44)	120.0
C(45)-C(44)-H(44)	120.0
C(46)-C(45)-C(44)	120.0
C(46)-C(45)-H(45)	120.0

C(44)-C(45)-H(45)	120.0
C(47)-C(46)-C(45)	120.0
C(47)-C(46)-H(46)	120.0
C(45)-C(46)-H(46)	120.0
C(46)-C(47)-C(48)	120.0
C(46)-C(47)-H(47)	120.0
C(48)-C(47)-H(47)	120.0
C(47)-C(48)-C(43)	120.0
C(47)-C(48)-H(48)	120.0
C(43)-C(48)-H(48)	120.0
C(44A)-C(43A)-C(48A)	120.0
C(44A)-C(43A)-C(42)	122(2)
C(48A)-C(43A)-C(42)	117(2)
C(45A)-C(44A)-C(43A)	120.0
C(45A)-C(44A)-H(44A)	120.0
C(43A)-C(44A)-H(44A)	120.0
C(44A)-C(45A)-C(46A)	120.0
C(44A)-C(45A)-H(45A)	120.0
C(46A)-C(45A)-H(45A)	120.0
C(47A)-C(46A)-C(45A)	120.0
C(47A)-C(46A)-H(46A)	120.0
C(45A)-C(46A)-H(46A)	120.0
C(46A)-C(47A)-C(48A)	120.0
C(46A)-C(47A)-H(47A)	120.0
C(48A)-C(47A)-H(47A)	120.0
C(47A)-C(48A)-C(43A)	120.0
C(47A)-C(48A)-H(48A)	120.0
C(43A)-C(48A)-H(48A)	120.0
O(10)-C(49)-O(11)	121.9(6)
O(10)-C(49)-C(50)	120.3(6)
O(11)-C(49)-C(50)	117.8(6)
N(4)-C(50)-C(49)	110.4(5)
N(4)-C(50)-C(58)	113.8(6)
C(49)-C(50)-C(58)	110.1(6)
N(4)-C(50)-H(50)	107.4
C(49)-C(50)-H(50)	107.4

C(58)-C(50)-H(50)	107.4
O(12)-C(51)-N(4)	123.8(7)
O(12)-C(51)-C(52)	123.5(6)
N(4)-C(51)-C(52)	112.7(7)
C(53)-C(52)-C(57)	119.1(7)
C(53)-C(52)-C(51)	119.3(6)
C(57)-C(52)-C(51)	121.6(7)
C(52)-C(53)-C(54)	122.2(6)
C(52)-C(53)-I(4)	112.1(5)
C(54)-C(53)-I(4)	125.7(6)
C(53)-C(54)-C(55)	117.0(7)
C(53)-C(54)-H(54)	121.5
C(55)-C(54)-H(54)	121.5
C(56)-C(55)-C(54)	121.3(7)
C(56)-C(55)-H(55)	119.3
C(54)-C(55)-H(55)	119.3
C(57)-C(56)-C(55)	119.9(7)
C(57)-C(56)-H(56)	120.0
C(55)-C(56)-H(56)	120.0
C(56)-C(57)-C(52)	120.4(7)
C(56)-C(57)-H(57)	119.8
C(52)-C(57)-H(57)	119.8
C(59)-C(58)-C(50)	113.2(6)
C(59)-C(58)-H(58A)	108.9
C(50)-C(58)-H(58A)	108.9
C(59)-C(58)-H(58B)	108.9
C(50)-C(58)-H(58B)	108.9
H(58A)-C(58)-H(58B)	107.7
C(60)-C(59)-C(64)	120.0
C(60)-C(59)-C(58)	122.0(4)
C(64)-C(59)-C(58)	117.9(4)
C(61)-C(60)-C(59)	120.0
C(61)-C(60)-H(60)	120.0
C(59)-C(60)-H(60)	120.0
C(60)-C(61)-C(62)	120.0
C(60)-C(61)-H(61)	120.0

C(62)-C(61)-H(61)	120.0
C(63)-C(62)-C(61)	120.0
C(63)-C(62)-H(62)	120.0
C(61)-C(62)-H(62)	120.0
C(62)-C(63)-C(64)	120.0
C(62)-C(63)-H(63)	120.0
C(64)-C(63)-H(63)	120.0
C(63)-C(64)-C(59)	120.0
C(63)-C(64)-H(64)	120.0
C(59)-C(64)-H(64)	120.0
O(13)-C(65)-O(14)	123.0(7)
O(13)-C(65)-C(66)	120.2(6)
O(14)-C(65)-C(66)	116.7(6)
N(5)-C(66)-C(65)	110.8(5)
N(5)-C(66)-C(74)	112.7(6)
C(65)-C(66)-C(74)	109.7(6)
N(5)-C(66)-H(66)	107.8
C(65)-C(66)-H(66)	107.8
C(74)-C(66)-H(66)	107.8
O(15)-C(67)-N(5)	123.4(7)
O(15)-C(67)-C(68)	122.9(6)
N(5)-C(67)-C(68)	113.8(6)
C(69)-C(68)-C(73)	119.0(7)
C(69)-C(68)-C(67)	118.8(6)
C(73)-C(68)-C(67)	122.2(6)
C(68)-C(69)-C(70)	122.2(6)
C(68)-C(69)-I(5)	112.1(5)
C(70)-C(69)-I(5)	125.7(5)
C(71)-C(70)-C(69)	117.5(7)
C(71)-C(70)-H(70)	121.3
C(69)-C(70)-H(70)	121.3
C(70)-C(71)-C(72)	120.9(7)
C(70)-C(71)-H(71)	119.6
C(72)-C(71)-H(71)	119.6
C(73)-C(72)-C(71)	120.4(7)
C(73)-C(72)-H(72)	119.8

C(71)-C(72)-H(72)	119.8
C(72)-C(73)-C(68)	120.0(7)
C(72)-C(73)-H(73)	120.0
C(68)-C(73)-H(73)	120.0
C(75)-C(74)-C(66)	113.3(5)
C(75)-C(74)-H(74A)	108.9
C(66)-C(74)-H(74A)	108.9
C(75)-C(74)-H(74B)	108.9
C(66)-C(74)-H(74B)	108.9
H(74A)-C(74)-H(74B)	107.7
C(76)-C(75)-C(80)	120.0
C(76)-C(75)-C(74)	120.0(4)
C(80)-C(75)-C(74)	119.9(4)
C(77)-C(76)-C(75)	120.0
C(77)-C(76)-H(76)	120.0
C(75)-C(76)-H(76)	120.0
C(76)-C(77)-C(78)	120.0
C(76)-C(77)-H(77)	120.0
C(78)-C(77)-H(77)	120.0
C(79)-C(78)-C(77)	120.0
C(79)-C(78)-H(78)	120.0
C(77)-C(78)-H(78)	120.0
C(80)-C(79)-C(78)	120.0
C(80)-C(79)-H(79)	120.0
C(78)-C(79)-H(79)	120.0
C(79)-C(80)-C(75)	120.0
C(79)-C(80)-H(80)	120.0
C(75)-C(80)-H(80)	120.0
O(17)-C(81)-O(16)	124.3(6)
O(17)-C(81)-C(82)#3	119.8(6)
O(16)-C(81)-C(82)#3	115.8(6)
N(6)-C(82)-C(81)#1	109.4(5)
N(6)-C(82)-C(90)	112.3(6)
C(81)#1-C(82)-C(90)	108.6(5)
N(6)-C(82)-H(82)	108.8
C(81)#1-C(82)-H(82)	108.8

C(90)-C(82)-H(82)	108.8
O(18)-C(83)-N(6)	123.6(6)
O(18)-C(83)-C(84)	122.4(6)
N(6)-C(83)-C(84)	114.0(5)
C(89)-C(84)-C(85)	118.9(6)
C(89)-C(84)-C(83)	122.2(6)
C(85)-C(84)-C(83)	118.8(6)
C(86)-C(85)-C(84)	122.5(6)
C(86)-C(85)-I(6)	125.6(5)
C(84)-C(85)-I(6)	111.8(5)
C(85)-C(86)-C(87)	117.8(7)
C(85)-C(86)-H(86)	121.1
C(87)-C(86)-H(86)	121.1
C(86)-C(87)-C(88)	120.0(7)
C(86)-C(87)-H(87)	120.0
C(88)-C(87)-H(87)	120.0
C(89)-C(88)-C(87)	120.5(7)
C(89)-C(88)-H(88)	119.7
C(87)-C(88)-H(88)	119.7
C(88)-C(89)-C(84)	120.1(7)
C(88)-C(89)-H(89)	119.9
C(84)-C(89)-H(89)	119.9
C(91)-C(90)-C(82)	112.7(5)
C(91)-C(90)-H(90A)	109.1
C(82)-C(90)-H(90A)	109.1
C(91)-C(90)-H(90B)	109.1
C(82)-C(90)-H(90B)	109.1
H(90A)-C(90)-H(90B)	107.8
C(92)-C(91)-C(96)	120.0
C(92)-C(91)-C(90)	118.6(4)
C(96)-C(91)-C(90)	121.1(4)
C(91)-C(92)-C(93)	120.0
C(91)-C(92)-H(92)	120.0
C(93)-C(92)-H(92)	120.0
C(92)-C(93)-C(94)	120.0
C(92)-C(93)-H(93)	120.0

C(94)-C(93)-H(93)	120.0
C(95)-C(94)-C(93)	120.0
C(95)-C(94)-H(94)	120.0
C(93)-C(94)-H(94)	120.0
C(96)-C(95)-C(94)	120.0
C(96)-C(95)-H(95)	120.0
C(94)-C(95)-H(95)	120.0
C(95)-C(96)-C(91)	120.0
C(95)-C(96)-H(96)	120.0
C(91)-C(96)-H(96)	120.0
C(98)-C(97)-C(102)	120.0
C(98)-C(97)-B(1)	119.5(7)
C(102)-C(97)-B(1)	120.4(7)
C(99)-C(98)-C(97)	120.0
C(99)-C(98)-H(98)	120.0
C(97)-C(98)-H(98)	120.0
C(98)-C(99)-C(100)	120.0
C(98)-C(99)-C(103)	120.8(7)
C(100)-C(99)-C(103)	119.0(7)
C(101)-C(100)-C(99)	120.0
C(101)-C(100)-H(100)	120.0
C(99)-C(100)-H(100)	120.0
C(100)-C(101)-C(102)	120.0
C(100)-C(101)-C(104)	119.7(8)
C(102)-C(101)-C(104)	118.3(8)
C(101)-C(102)-C(97)	120.0
C(101)-C(102)-H(102)	120.0
C(97)-C(102)-H(102)	120.0
F(2)-C(103)-F(1)	105.3(11)
F(2)-C(103)-F(3)	106.9(11)
F(1)-C(103)-F(3)	107.2(12)
F(2)-C(103)-C(99)	111.4(11)
F(1)-C(103)-C(99)	111.9(11)
F(3)-C(103)-C(99)	113.7(11)
F(6)-C(104)-F(4)	108.8(13)
F(6)-C(104)-F(5)	109.6(13)

F(4)-C(104)-F(5)	105.6(11)
F(6)-C(104)-C(101)	100.6(13)
F(4)-C(104)-C(101)	114.2(11)
F(5)-C(104)-C(101)	117.7(13)
C(106)-C(105)-C(110)	120.0
C(106)-C(105)-B(1)	120.9(6)
C(110)-C(105)-B(1)	119.0(6)
C(105)-C(106)-C(107)	120.0
C(105)-C(106)-H(106)	120.0
C(107)-C(106)-H(106)	120.0
C(108)-C(107)-C(106)	120.0
C(108)-C(107)-C(111)	120.5(6)
C(106)-C(107)-C(111)	119.5(6)
C(109)-C(108)-C(107)	120.0
C(109)-C(108)-H(108)	120.0
C(107)-C(108)-H(108)	120.0
C(108)-C(109)-C(110)	120.0
C(108)-C(109)-C(112)	121.1(6)
C(110)-C(109)-C(112)	118.9(6)
C(109)-C(110)-C(105)	120.0
C(109)-C(110)-H(110)	120.0
C(105)-C(110)-H(110)	120.0
F(9)-C(111)-F(7)	108.1(9)
F(9)-C(111)-F(8)	106.2(9)
F(7)-C(111)-F(8)	106.2(9)
F(9)-C(111)-C(107)	112.2(8)
F(7)-C(111)-C(107)	112.1(8)
F(8)-C(111)-C(107)	111.7(9)
F(10)-C(112)-F(11)	106.2(9)
F(10)-C(112)-F(12)	107.0(9)
F(11)-C(112)-F(12)	105.6(9)
F(10)-C(112)-C(109)	114.2(8)
F(11)-C(112)-C(109)	110.9(9)
F(12)-C(112)-C(109)	112.4(9)
C(114)-C(113)-C(118)	120.0
C(114)-C(113)-B(1)	117.1(6)

C(118)-C(113)-B(1)	122.9(6)
C(113)-C(114)-C(115)	120.0
C(113)-C(114)-H(114)	120.0
C(115)-C(114)-H(114)	120.0
C(116)-C(115)-C(114)	120.0
C(116)-C(115)-C(119)	120.5(7)
C(114)-C(115)-C(119)	119.4(7)
C(115)-C(116)-C(117)	120.0
C(115)-C(116)-H(116)	120.0
C(117)-C(116)-H(116)	120.0
C(118)-C(117)-C(116)	120.0
C(118)-C(117)-C(120)	118.6(7)
C(116)-C(117)-C(120)	121.4(7)
C(117)-C(118)-C(113)	120.0
C(117)-C(118)-H(118)	120.0
C(113)-C(118)-H(118)	120.0
F(13)-C(119)-F(15)	104.6(10)
F(13)-C(119)-F(14)	108.2(11)
F(15)-C(119)-F(14)	103.9(10)
F(13)-C(119)-C(115)	113.3(11)
F(15)-C(119)-C(115)	115.1(10)
F(14)-C(119)-C(115)	111.0(10)
F(18)-C(120)-F(17)	105.5(9)
F(18)-C(120)-F(16)	105.7(9)
F(17)-C(120)-F(16)	107.9(9)
F(18)-C(120)-C(117)	111.4(9)
F(17)-C(120)-C(117)	112.7(9)
F(16)-C(120)-C(117)	113.2(9)
C(122)-C(121)-C(126)	120.0
C(122)-C(121)-B(1)	120.2(6)
C(126)-C(121)-B(1)	119.6(6)
C(121)-C(122)-C(123)	120.0
C(121)-C(122)-H(122)	120.0
C(123)-C(122)-H(122)	120.0
C(122)-C(123)-C(124)	120.0
C(122)-C(123)-C(127)	119.3(6)

C(124)-C(123)-C(127)	120.6(6)
C(125)-C(124)-C(123)	120.0
C(125)-C(124)-H(124)	120.0
C(123)-C(124)-H(124)	120.0
C(126)-C(125)-C(124)	120.0
C(126)-C(125)-C(128)	119.3(7)
C(124)-C(125)-C(128)	120.7(7)
C(125)-C(126)-C(121)	120.0
C(125)-C(126)-H(126)	120.0
C(121)-C(126)-H(126)	120.0
F(20)-C(127)-F(19)	109.2(10)
F(20)-C(127)-F(21)	105.2(10)
F(19)-C(127)-F(21)	107.5(9)
F(20)-C(127)-C(123)	109.4(10)
F(19)-C(127)-C(123)	112.5(8)
F(21)-C(127)-C(123)	112.8(9)
F(23)-C(128)-F(24)	106.4(10)
F(23)-C(128)-F(22)	106.0(10)
F(24)-C(128)-F(22)	104.4(10)
F(23)-C(128)-C(125)	112.5(9)
F(24)-C(128)-C(125)	113.0(10)
F(22)-C(128)-C(125)	113.8(10)
C(105)-B(1)-C(121)	113.2(8)
C(105)-B(1)-C(113)	109.5(8)
C(121)-B(1)-C(113)	111.2(8)
C(105)-B(1)-C(97)	107.8(8)
C(121)-B(1)-C(97)	109.3(9)
C(113)-B(1)-C(97)	105.6(9)
C(98A)-C(97A)-C(202)	120.0
C(98A)-C(97A)-B(1A)	118.3(8)
C(202)-C(97A)-B(1A)	121.7(8)
C(97A)-C(98A)-C(99A)	120.0
C(97A)-C(98A)-H(98A)	120.0
C(99A)-C(98A)-H(98A)	120.0
C(200)-C(99A)-C(98A)	120.0
C(200)-C(99A)-C(203)	118.5(8)

C(98A)-C(99A)-C(203)	121.3(8)
C(201)-C(200)-C(99A)	120.0
C(201)-C(200)-H(200)	120.0
C(99A)-C(200)-H(200)	120.0
C(200)-C(201)-C(202)	120.0
C(200)-C(201)-C(204)	118.7(9)
C(202)-C(201)-C(204)	119.4(9)
C(201)-C(202)-C(97A)	120.0
C(201)-C(202)-H(202)	120.0
C(97A)-C(202)-H(202)	120.0
F(2A)-C(203)-F(1A)	106.5(12)
F(2A)-C(203)-F(3A)	107.7(12)
F(1A)-C(203)-F(3A)	105.0(12)
F(2A)-C(203)-C(99A)	112.1(13)
F(1A)-C(203)-C(99A)	111.1(12)
F(3A)-C(203)-C(99A)	113.9(13)
F(4A)-C(204)-F(5A)	104.1(12)
F(4A)-C(204)-F(6A)	103.3(13)
F(5A)-C(204)-F(6A)	106.8(13)
F(4A)-C(204)-C(201)	119.8(13)
F(5A)-C(204)-C(201)	122.4(13)
F(6A)-C(204)-C(201)	97.7(14)
C(206)-C(205)-C(210)	120.0
C(206)-C(205)-B(1A)	118.4(7)
C(210)-C(205)-B(1A)	121.6(7)
C(205)-C(206)-C(207)	120.0
C(205)-C(206)-H(206)	120.0
C(207)-C(206)-H(206)	120.0
C(206)-C(207)-C(208)	120.0
C(206)-C(207)-C(211)	117.9(7)
C(208)-C(207)-C(211)	121.6(7)
C(209)-C(208)-C(207)	120.0
C(209)-C(208)-H(208)	120.0
C(207)-C(208)-H(208)	120.0
C(208)-C(209)-C(210)	120.0
C(208)-C(209)-C(212)	120.8(7)

C(210)-C(209)-C(212)	119.2(7)
C(209)-C(210)-C(205)	120.0
C(209)-C(210)-H(210)	120.0
C(205)-C(210)-H(210)	120.0
F(8A)-C(211)-F(9A)	107.7(11)
F(8A)-C(211)-F(7A)	106.1(10)
F(9A)-C(211)-F(7A)	105.1(10)
F(8A)-C(211)-C(207)	113.3(10)
F(9A)-C(211)-C(207)	112.7(11)
F(7A)-C(211)-C(207)	111.3(10)
F(11A)-C(212)-F(10A)	105.9(11)
F(11A)-C(212)-F(12A)	108.0(11)
F(10A)-C(212)-F(12A)	105.0(10)
F(11A)-C(212)-C(209)	111.4(12)
F(10A)-C(212)-C(209)	113.7(10)
F(12A)-C(212)-C(209)	112.4(10)
C(214)-C(213)-C(218)	120.0
C(214)-C(213)-B(1A)	121.5(7)
C(218)-C(213)-B(1A)	118.4(7)
C(215)-C(214)-C(213)	120.0
C(215)-C(214)-H(214)	120.0
C(213)-C(214)-H(214)	120.0
C(216)-C(215)-C(214)	120.0
C(216)-C(215)-C(219)	118.4(7)
C(214)-C(215)-C(219)	121.6(7)
C(217)-C(216)-C(215)	120.0
C(217)-C(216)-H(216)	120.0
C(215)-C(216)-H(216)	120.0
C(216)-C(217)-C(218)	120.0
C(216)-C(217)-C(220)	123.3(8)
C(218)-C(217)-C(220)	116.7(8)
C(217)-C(218)-C(213)	120.0
C(217)-C(218)-H(218)	120.0
C(213)-C(218)-H(218)	120.0
F(13A)-C(219)-F(15A)	104.9(10)
F(13A)-C(219)-F(14A)	105.2(10)

F(15A)-C(219)-F(14A)	105.6(10)
F(13A)-C(219)-C(215)	113.7(9)
F(15A)-C(219)-C(215)	115.2(11)
F(14A)-C(219)-C(215)	111.4(11)
F(18A)-C(220)-F(17A)	106.7(12)
F(18A)-C(220)-F(16A)	108.6(12)
F(17A)-C(220)-F(16A)	104.9(12)
F(18A)-C(220)-C(217)	113.3(11)
F(17A)-C(220)-C(217)	113.0(12)
F(16A)-C(220)-C(217)	110.0(12)
C(222)-C(221)-C(226)	120.0
C(222)-C(221)-B(1A)	118.5(7)
C(226)-C(221)-B(1A)	121.2(7)
C(221)-C(222)-C(223)	120.0
C(221)-C(222)-H(222)	120.0
C(223)-C(222)-H(222)	120.0
C(222)-C(223)-C(224)	120.0
C(222)-C(223)-C(227)	117.4(8)
C(224)-C(223)-C(227)	122.6(8)
C(225)-C(224)-C(223)	120.0
C(225)-C(224)-H(224)	120.0
C(223)-C(224)-H(224)	120.0
C(226)-C(225)-C(224)	120.0
C(226)-C(225)-C(228)	120.6(7)
C(224)-C(225)-C(228)	119.4(7)
C(225)-C(226)-C(221)	120.0
C(225)-C(226)-H(226)	120.0
C(221)-C(226)-H(226)	120.0
F(20A)-C(227)-F(19A)	108.0(11)
F(20A)-C(227)-F(21A)	107.8(11)
F(19A)-C(227)-F(21A)	100.8(10)
F(20A)-C(227)-C(223)	113.4(10)
F(19A)-C(227)-C(223)	112.7(11)
F(21A)-C(227)-C(223)	113.3(11)
F(24A)-C(228)-F(23A)	106.6(10)
F(24A)-C(228)-F(22A)	105.3(9)

F(23A)-C(228)-F(22A)	105.6(10)
F(24A)-C(228)-C(225)	114.7(10)
F(23A)-C(228)-C(225)	111.8(11)
F(22A)-C(228)-C(225)	112.3(9)
C(205)-B(1A)-C(221)	111.6(8)
C(205)-B(1A)-C(213)	108.8(9)
C(221)-B(1A)-C(213)	109.3(9)
C(205)-B(1A)-C(97A)	108.6(10)
C(221)-B(1A)-C(97A)	107.9(9)
C(213)-B(1A)-C(97A)	110.6(9)

Symmetry transformations used to generate equivalent atoms:
#1 -x+2,y+1/2,-z+1/2   #2 x-1/2,-y+1/2,-z+1   #3 -x+2,y-1/2,-z+1/2
#4 x+1/2,-y+1/2,-z+1

Table 4. Anisotropic displacement parameters ($Å^2$x 10^3) for 16157ds. The anisotropic displacement factor exponent takes the form: $-2p^2[h^2 a^{*2}U^{11} + ... + 2 h k a^* b^* U^{12}]$

	U^{11}	U^{22}	U^{33}	U^{23}	U^{13}	U^{12}
I(1)	32(1)	29(1)	31(1)	4(1)	1(1)	-2(1)
I(2)	29(1)	23(1)	40(1)	2(1)	2(1)	0(1)
I(3)	30(1)	33(1)	32(1)	2(1)	1(1)	-6(1)
I(4)	28(1)	21(1)	30(1)	-5(1)	2(1)	4(1)
I(5)	26(1)	25(1)	24(1)	-1(1)	-2(1)	1(1)
I(6)	26(1)	24(1)	27(1)	8(1)	-2(1)	-5(1)
Na(1)	38(2)	24(1)	26(1)	2(1)	3(1)	-1(1)
O(1)	37(3)	35(3)	33(3)	-1(2)	3(2)	-7(2)
O(2)	40(3)	37(3)	42(3)	-2(2)	3(2)	-10(2)
O(3)	43(3)	40(3)	40(3)	9(2)	4(2)	-11(2)
O(4)	40(3)	37(3)	39(3)	-2(2)	2(2)	-7(2)
O(5)	51(3)	31(3)	44(3)	-6(2)	13(3)	-10(2)
O(6)	67(4)	64(4)	49(4)	-17(3)	6(3)	-26(4)
O(7)	35(3)	28(2)	43(3)	1(2)	7(2)	-3(2)
O(8)	46(3)	38(3)	36(3)	0(2)	6(2)	-10(2)
O(9)	45(3)	40(3)	32(3)	5(2)	5(2)	1(2)
O(10)	39(3)	29(2)	35(3)	-10(2)	-6(2)	14(2)
O(11)	36(3)	27(2)	36(3)	-10(2)	-7(2)	11(2)
O(12)	37(3)	32(3)	39(3)	7(2)	5(2)	18(2)
O(13)	36(3)	32(3)	35(3)	-10(2)	-10(2)	4(2)
O(14)	36(3)	30(2)	32(3)	-10(2)	-3(2)	5(2)
O(15)	35(3)	43(3)	28(3)	-1(2)	-1(2)	-7(2)
O(16)	37(3)	26(2)	28(2)	-5(2)	-3(2)	7(2)
O(17)	40(3)	38(3)	30(3)	-13(2)	-7(2)	15(2)
O(18)	34(3)	26(2)	43(3)	13(2)	-1(2)	-5(2)
O(19)	58(4)	35(3)	69(4)	15(3)	-22(3)	-9(3)
O(20)	59(9)	109(12)	45(7)	-25(7)	-14(6)	14(8)
O(20A)	88(13)	56(10)	56(9)	-13(8)	-5(9)	-12(9)
N(1)	34(3)	35(3)	33(3)	2(3)	0(3)	-9(3)
N(2)	37(3)	27(3)	45(4)	-3(3)	-2(3)	-3(3)
N(3)	36(3)	32(3)	30(3)	5(2)	4(3)	-7(3)

N(4)	30(3)	23(3)	29(3)	-2(2)	1(2)	9(2)
N(5)	29(3)	29(3)	26(3)	-4(2)	-4(2)	-3(2)
N(6)	27(3)	20(2)	31(3)	9(2)	0(2)	-7(2)
C(1)	34(4)	31(3)	37(4)	6(3)	-2(3)	-3(3)
C(2)	34(4)	37(4)	33(4)	3(3)	4(3)	-9(3)
C(3)	30(4)	41(4)	40(4)	-1(3)	-1(3)	-7(3)
C(4)	28(4)	41(4)	34(4)	9(3)	-2(3)	-5(3)
C(5)	36(4)	38(4)	37(4)	8(3)	-2(3)	-8(3)
C(6)	30(4)	48(4)	27(3)	4(3)	2(3)	-4(3)
C(7)	37(4)	44(4)	41(4)	0(3)	-3(3)	-2(3)
C(8)	42(5)	64(6)	41(5)	-5(4)	8(4)	-8(4)
C(9)	50(5)	62(6)	38(4)	3(4)	5(4)	-16(4)
C(10)	40(4)	48(5)	36(4)	6(4)	2(3)	-9(4)
C(11)	30(4)	46(5)	57(7)	8(5)	-7(5)	-1(4)
C(12)	45(6)	53(5)	60(7)	14(5)	-10(5)	3(5)
C(13)	54(6)	55(6)	70(7)	11(5)	-9(5)	2(5)
C(14)	55(6)	52(6)	77(7)	12(6)	-11(6)	8(5)
C(15)	48(5)	63(6)	71(7)	2(6)	-12(6)	8(5)
C(16)	34(5)	51(5)	65(7)	1(5)	-5(5)	2(4)
C(11A)	35(6)	46(6)	67(8)	7(6)	-8(7)	-1(6)
C(12A)	47(7)	58(7)	74(9)	14(7)	-10(7)	-3(6)
C(13A)	52(7)	60(7)	73(8)	13(7)	-10(7)	0(7)
C(14A)	55(7)	60(7)	74(8)	8(7)	-12(7)	7(7)
C(15A)	50(7)	60(7)	74(9)	2(7)	-8(7)	6(6)
C(16A)	39(7)	53(7)	71(8)	-1(7)	-9(7)	3(6)
C(17)	33(4)	30(3)	38(4)	-3(3)	2(3)	-3(3)
C(18)	38(4)	27(3)	43(4)	-1(3)	2(3)	-1(3)
C(19)	41(4)	36(4)	52(5)	-9(4)	-3(4)	-9(3)
C(20)	48(5)	43(5)	56(5)	-9(4)	4(4)	-14(4)
C(21)	35(4)	28(4)	54(5)	-1(3)	3(3)	-8(3)
C(22)	51(5)	35(4)	59(5)	-3(4)	11(4)	-13(4)
C(23)	80(8)	54(6)	76(7)	-9(5)	35(6)	-34(6)
C(24)	123(11)	95(9)	83(8)	-28(7)	40(8)	-81(9)
C(25)	86(8)	76(8)	78(8)	-28(6)	21(6)	-54(7)
C(26)	49(5)	29(4)	60(5)	2(4)	13(4)	11(3)
C(27)	38(5)	31(6)	67(5)	2(4)	8(5)	13(5)

C(28)	45(7)	34(5)	76(6)	6(5)	5(5)	12(5)
C(29)	53(7)	40(6)	76(6)	14(5)	7(5)	5(5)
C(30)	60(6)	44(7)	79(6)	18(5)	-4(5)	5(6)
C(31)	54(5)	38(6)	80(6)	16(5)	-10(5)	0(6)
C(32)	43(5)	35(7)	77(6)	11(5)	-3(5)	8(6)
C(27A)	39(7)	33(7)	71(7)	6(6)	7(6)	11(7)
C(28A)	40(8)	32(7)	73(7)	8(6)	5(6)	10(7)
C(29A)	48(8)	34(8)	76(7)	16(6)	6(7)	1(7)
C(30A)	55(7)	40(9)	78(7)	16(7)	-1(7)	1(8)
C(31A)	54(7)	42(9)	79(7)	16(7)	-6(7)	8(8)
C(32A)	42(6)	39(8)	75(7)	10(7)	-2(6)	10(7)
C(33)	32(4)	30(3)	38(4)	7(3)	0(3)	0(3)
C(34)	30(4)	29(4)	38(4)	6(3)	8(3)	0(3)
C(35)	31(4)	41(4)	32(4)	6(3)	0(3)	4(3)
C(36)	42(4)	35(4)	32(4)	0(3)	-4(3)	-1(3)
C(37)	39(4)	36(4)	30(4)	-2(3)	-4(3)	4(3)
C(38)	46(5)	38(4)	46(5)	0(4)	0(4)	-4(4)
C(39)	62(6)	38(5)	65(6)	-10(4)	4(5)	-9(4)
C(40)	64(6)	47(5)	52(5)	-15(4)	3(5)	-1(4)
C(41)	43(4)	43(4)	42(4)	-3(4)	-1(4)	1(4)
C(42)	35(4)	43(4)	41(4)	12(3)	1(3)	7(3)
C(43)	35(5)	110(9)	50(5)	25(6)	-3(4)	15(5)
C(44)	42(6)	109(9)	61(6)	22(6)	-17(5)	1(6)
C(45)	53(6)	125(10)	79(7)	24(7)	-20(6)	-3(7)
C(46)	57(7)	144(11)	81(8)	26(8)	-9(6)	5(7)
C(47)	53(6)	135(10)	77(7)	15(7)	0(6)	17(6)
C(48)	45(6)	127(10)	65(6)	16(7)	0(5)	25(6)
C(43A)	39(7)	114(10)	57(7)	21(7)	-6(6)	14(7)
C(44A)	43(7)	116(10)	65(7)	21(8)	-17(6)	5(7)
C(45A)	50(7)	128(10)	76(7)	19(8)	-16(6)	8(7)
C(46A)	54(8)	132(11)	82(9)	22(9)	-9(8)	19(8)
C(47A)	53(8)	130(11)	76(8)	16(9)	-2(8)	24(8)
C(48A)	43(7)	123(10)	66(7)	17(8)	-2(7)	21(7)
C(49)	26(3)	24(3)	34(4)	-6(3)	2(3)	6(3)
C(50)	35(4)	26(3)	28(3)	-5(3)	-4(3)	8(3)
C(51)	43(4)	22(3)	35(4)	3(3)	15(3)	5(3)

C(52)	43(4)	18(3)	33(3)	9(2)	12(3)	8(3)
C(53)	39(4)	20(3)	30(3)	-3(3)	14(3)	4(3)
C(54)	48(5)	27(3)	35(4)	-8(3)	6(3)	3(3)
C(55)	55(5)	33(4)	36(4)	-11(3)	4(4)	1(4)
C(56)	58(5)	33(4)	36(4)	-1(3)	18(4)	14(4)
C(57)	50(5)	29(3)	33(4)	8(3)	13(3)	16(3)
C(58)	50(5)	31(4)	32(4)	-3(3)	-1(3)	13(3)
C(59)	49(5)	33(4)	27(3)	3(3)	8(3)	6(3)
C(60)	53(5)	38(4)	44(5)	-6(4)	7(4)	-8(4)
C(61)	50(5)	59(6)	46(5)	-5(4)	7(4)	-12(4)
C(62)	79(7)	51(6)	56(6)	-4(5)	17(5)	-26(5)
C(63)	100(9)	42(5)	75(7)	-14(5)	40(7)	-15(6)
C(64)	79(7)	35(4)	48(5)	0(4)	22(5)	-3(4)
C(65)	30(4)	30(3)	29(3)	-7(3)	3(3)	2(3)
C(66)	31(4)	29(3)	29(3)	-4(3)	0(3)	2(3)
C(67)	30(3)	30(3)	23(3)	3(3)	0(3)	-8(3)
C(68)	25(3)	30(3)	29(3)	6(3)	1(3)	-6(3)
C(69)	26(3)	30(3)	30(4)	6(3)	-5(3)	1(3)
C(70)	31(4)	36(4)	31(4)	0(3)	-3(3)	4(3)
C(71)	40(4)	31(4)	51(5)	10(3)	-9(4)	4(3)
C(72)	39(4)	38(4)	37(4)	11(3)	-12(3)	0(3)
C(73)	37(4)	35(4)	28(3)	7(3)	-8(3)	-11(3)
C(74)	34(4)	33(4)	32(4)	-4(3)	-4(3)	-10(3)
C(75)	39(4)	23(3)	35(4)	0(3)	-3(3)	-9(3)
C(76)	55(6)	37(4)	72(6)	15(4)	15(5)	8(4)
C(77)	73(7)	49(5)	76(7)	2(5)	41(6)	-2(5)
C(78)	90(9)	70(7)	47(6)	-9(5)	19(5)	-35(6)
C(79)	88(8)	64(6)	30(4)	11(4)	-12(5)	-31(6)
C(80)	45(5)	46(5)	39(4)	8(4)	-8(4)	-9(4)
C(81)	28(3)	23(3)	30(3)	-4(3)	-1(3)	-1(3)
C(82)	26(3)	23(3)	29(3)	8(3)	-1(3)	-6(3)
C(83)	27(3)	18(3)	32(3)	7(2)	4(3)	-2(2)
C(84)	24(3)	24(3)	30(3)	4(2)	2(3)	3(3)
C(85)	24(3)	27(3)	25(3)	7(3)	-1(2)	2(2)
C(86)	36(4)	36(4)	33(4)	7(3)	-7(3)	-8(3)
C(87)	34(4)	41(4)	40(4)	3(3)	-10(3)	-4(3)

C(88)	37(4)	39(4)	33(4)	7(3)	-4(3)	4(3)
C(89)	30(4)	31(3)	33(4)	9(3)	3(3)	2(3)
C(90)	26(3)	33(3)	29(3)	10(3)	2(3)	-4(3)
C(91)	29(4)	32(4)	32(3)	6(3)	0(3)	-2(3)
C(92)	32(4)	31(4)	34(4)	10(3)	4(3)	3(3)
C(93)	42(4)	45(4)	31(4)	-4(3)	3(3)	0(4)
C(94)	69(6)	37(4)	58(6)	-3(4)	14(5)	7(4)
C(95)	93(8)	34(4)	46(5)	9(4)	5(5)	14(5)
C(96)	51(5)	42(4)	36(4)	4(3)	0(4)	11(4)
F(1)	63(5)	91(7)	91(6)	-9(6)	9(5)	-12(6)
F(2)	71(5)	89(6)	94(7)	-11(6)	-24(6)	2(5)
F(3)	67(6)	98(6)	90(7)	-7(6)	-21(6)	-24(5)
F(4)	127(7)	148(8)	75(7)	-46(6)	-13(6)	-27(7)
F(5)	111(8)	155(8)	101(7)	-39(6)	18(7)	-16(7)
F(6)	140(8)	137(8)	101(7)	-46(7)	-2(7)	3(7)
F(7)	54(4)	33(5)	62(5)	-6(4)	10(4)	7(4)
F(8)	51(4)	34(5)	64(5)	-9(5)	-12(4)	7(4)
F(9)	58(4)	33(4)	59(4)	-1(4)	-11(4)	4(3)
F(10)	57(5)	52(5)	45(5)	-5(4)	10(4)	2(4)
F(11)	61(6)	38(5)	47(5)	2(4)	0(4)	-10(4)
F(12)	59(4)	56(4)	54(5)	14(4)	2(4)	4(4)
F(13)	110(7)	92(7)	103(6)	28(6)	-20(6)	15(6)
F(14)	74(7)	89(7)	118(8)	43(6)	-34(6)	2(6)
F(15)	88(7)	78(7)	103(7)	35(6)	-17(6)	11(6)
F(16)	60(5)	72(5)	70(5)	13(4)	11(4)	-20(4)
F(17)	52(6)	72(6)	50(5)	-20(5)	7(5)	-20(5)
F(18)	49(5)	70(5)	60(5)	-23(4)	14(4)	-5(4)
F(19)	44(4)	44(4)	58(4)	10(3)	-10(4)	-7(3)
F(20)	50(6)	43(5)	65(6)	-7(4)	-19(5)	-7(4)
F(21)	57(5)	38(4)	68(5)	9(4)	-12(4)	8(4)
F(22)	69(5)	90(6)	80(5)	-20(5)	4(5)	15(5)
F(23)	64(5)	71(6)	89(6)	-25(5)	14(5)	20(5)
F(24)	72(5)	73(6)	79(6)	-3(5)	15(5)	11(5)
C(97)	45(3)	62(3)	43(3)	-8(3)	-2(3)	-14(3)
C(98)	48(3)	70(4)	49(4)	-8(3)	-4(3)	-15(3)
C(99)	57(3)	83(4)	58(3)	-10(3)	-7(3)	-19(3)

C(100)	64(4)	92(4)	57(4)	-18(4)	-5(4)	-23(4)
C(101)	69(4)	97(4)	58(4)	-24(4)	0(3)	-22(4)
C(102)	55(4)	79(4)	48(4)	-18(4)	1(3)	-20(4)
C(103)	63(4)	88(4)	72(4)	-9(4)	-13(4)	-16(4)
C(104)	93(5)	120(5)	74(4)	-34(4)	0(4)	-19(4)
C(105)	34(3)	34(3)	38(3)	-5(3)	-4(3)	-2(3)
C(106)	33(3)	35(3)	42(3)	-5(3)	-5(3)	2(3)
C(107)	36(3)	33(3)	46(3)	-6(3)	-4(3)	5(3)
C(108)	34(4)	32(4)	39(4)	-8(4)	-5(4)	3(4)
C(109)	37(4)	33(4)	38(4)	-3(3)	-5(3)	0(3)
C(110)	34(3)	29(3)	34(3)	-4(3)	-4(3)	0(3)
C(111)	43(4)	34(4)	54(4)	-7(3)	-3(3)	6(3)
C(112)	47(4)	43(4)	42(4)	-1(4)	1(4)	-1(4)
C(113)	38(3)	41(3)	39(3)	-5(3)	2(3)	-1(3)
C(114)	42(4)	43(4)	50(4)	1(4)	1(4)	1(4)
C(115)	57(5)	52(5)	63(5)	10(4)	2(4)	6(4)
C(116)	51(5)	45(5)	51(5)	-1(5)	8(5)	-1(5)
C(117)	43(3)	45(3)	41(3)	-9(3)	10(3)	-5(3)
C(118)	39(3)	40(3)	37(4)	-8(3)	8(3)	-5(3)
C(119)	73(5)	71(5)	84(5)	25(5)	-8(5)	12(5)
C(120)	47(4)	53(4)	43(4)	-11(3)	12(4)	-8(4)
C(121)	38(3)	36(3)	40(3)	-8(3)	-2(3)	-1(3)
C(122)	38(4)	32(3)	44(4)	-6(3)	-8(3)	-2(3)
C(123)	40(3)	33(3)	49(3)	-6(3)	-10(3)	0(3)
C(124)	42(4)	42(4)	56(5)	-10(4)	-4(4)	6(4)
C(125)	48(3)	50(4)	54(4)	-14(3)	3(3)	8(3)
C(126)	42(3)	44(4)	44(4)	-15(3)	1(3)	4(3)
C(127)	44(4)	34(3)	57(4)	-3(3)	-11(4)	0(3)
C(128)	58(4)	62(4)	64(4)	-15(4)	7(4)	12(4)
B(1)	37(3)	41(3)	38(3)	-5(3)	-2(3)	-5(3)
F(1A)	62(6)	83(7)	92(6)	-15(6)	-1(6)	-8(6)
F(2A)	84(6)	85(7)	82(7)	-9(6)	-18(7)	10(6)
F(3A)	66(6)	93(7)	91(7)	-2(6)	-17(6)	-25(6)
F(4A)	119(8)	136(8)	97(7)	-50(7)	-6(7)	-11(7)
F(5A)	82(7)	116(7)	75(6)	-57(6)	18(6)	-13(6)
F(6A)	115(8)	161(8)	86(7)	-30(7)	-2(7)	-17(7)

F(7A)	56(5)	51(5)	51(5)	-6(4)	9(4)	2(4)
F(8A)	62(6)	61(6)	66(6)	21(5)	29(5)	8(5)
F(9A)	55(6)	58(6)	56(6)	-3(5)	4(5)	-10(5)
F(10A)	57(5)	50(5)	65(5)	20(5)	-10(5)	-8(4)
F(11A)	50(7)	41(6)	68(7)	0(5)	-13(6)	-7(5)
F(12A)	57(5)	64(5)	66(5)	-1(4)	-5(4)	6(4)
F(13A)	53(4)	39(4)	55(5)	-2(4)	8(4)	-1(4)
F(14A)	49(5)	38(7)	64(6)	-5(6)	-6(5)	-6(5)
F(15A)	49(5)	52(6)	74(6)	-12(5)	-2(5)	13(5)
F(16A)	101(7)	102(8)	106(7)	39(7)	-30(6)	2(7)
F(17A)	92(6)	85(6)	95(6)	37(6)	5(6)	8(6)
F(18A)	136(9)	96(8)	104(9)	37(7)	-2(8)	41(8)
F(19A)	62(5)	63(5)	72(5)	-4(4)	14(5)	20(4)
F(20A)	72(6)	70(6)	79(6)	3(6)	25(6)	17(5)
F(21A)	81(7)	53(6)	80(6)	-27(6)	17(6)	-3(6)
F(22A)	44(5)	58(5)	55(5)	4(4)	12(4)	-4(4)
F(23A)	53(7)	60(6)	40(5)	-7(5)	-10(5)	-3(5)
F(24A)	47(5)	51(5)	39(5)	-5(4)	15(4)	-3(4)
C(97A)	44(3)	62(3)	43(3)	-9(3)	-2(3)	-16(3)
C(98A)	49(4)	71(4)	49(4)	-8(3)	-4(3)	-15(3)
C(99A)	57(3)	84(4)	58(3)	-10(3)	-7(3)	-19(3)
C(200)	65(4)	93(4)	56(4)	-18(4)	-5(4)	-22(4)
C(201)	71(4)	99(4)	58(4)	-24(4)	0(3)	-21(4)
C(202)	56(4)	80(4)	49(4)	-18(4)	-1(4)	-20(4)
C(203)	63(4)	87(4)	72(4)	-9(4)	-12(4)	-16(4)
C(204)	93(5)	120(5)	73(4)	-33(4)	0(4)	-18(4)
C(205)	36(3)	34(3)	38(3)	-7(3)	-4(3)	-4(3)
C(206)	39(4)	32(4)	37(3)	-6(3)	-2(3)	-3(3)
C(207)	40(4)	34(4)	36(4)	-1(4)	-1(4)	-3(4)
C(208)	40(5)	35(5)	38(5)	-2(4)	2(4)	-3(4)
C(209)	41(3)	33(3)	46(4)	-3(3)	-7(3)	-3(3)
C(210)	37(4)	33(3)	42(4)	-6(3)	-7(3)	-2(3)
C(211)	51(4)	46(4)	44(5)	2(4)	2(4)	-3(4)
C(212)	44(4)	34(4)	51(4)	1(4)	-8(4)	-2(4)
C(213)	35(3)	40(3)	41(3)	-4(3)	-2(3)	-2(3)
C(214)	33(3)	36(3)	43(3)	-4(3)	-6(3)	0(3)

C(215)	39(3)	34(3)	52(3)	-3(3)	-4(3)	4(3)
C(216)	45(5)	41(5)	60(5)	9(5)	-3(5)	6(5)
C(217)	50(5)	52(5)	63(5)	17(5)	-5(5)	7(5)
C(218)	39(4)	44(4)	47(4)	3(4)	-1(4)	-1(4)
C(219)	42(4)	34(4)	56(4)	-5(4)	-1(4)	7(3)
C(220)	75(5)	72(5)	81(5)	29(5)	-10(5)	17(5)
C(221)	38(3)	41(3)	38(3)	-8(3)	1(3)	-4(3)
C(222)	44(4)	44(4)	43(4)	-11(3)	1(3)	2(3)
C(223)	49(4)	52(4)	51(4)	-13(3)	6(3)	9(3)
C(224)	41(5)	50(5)	42(5)	-10(5)	8(4)	3(5)
C(225)	41(3)	47(4)	39(3)	-10(3)	8(3)	-5(3)
C(226)	38(4)	40(4)	35(4)	-8(3)	6(3)	-6(3)
C(227)	59(4)	61(4)	63(4)	-15(4)	9(4)	12(4)
C(228)	44(4)	52(4)	43(4)	-10(4)	11(4)	-6(4)
B(1A)	37(3)	42(3)	39(3)	-5(3)	-2(3)	-5(3)

Table 5. Hydrogen coordinates (x 10^4) and isotropic displacement parameters (Å^{2}x 10^3) for 16157ds.

	x	y	z	U(eq)
H(2)	4074	3040	5239	42
H(3A)	3482	3444	4496	45
H(3B)	3092	3398	5043	45
H(3C)	3456	3377	4525	45
H(3D)	3092	3444	5079	45
H(7)	4175	5182	6906	49
H(8)	3551	4985	7649	59
H(9)	3000	4149	7707	60
H(10)	3061	3493	7027	49
H(12)	3848	4369	4184	63
H(13)	3772	5344	4225	72
H(14)	3219	5767	4925	74
H(15)	2744	5216	5583	73
H(16)	2821	4241	5542	60
H(12A)	3654	4265	3978	72
H(13A)	3455	5223	3888	74
H(14A)	3019	5722	4603	76
H(15A)	2782	5263	5406	74
H(16A)	2981	4306	5496	65
H(18)	5747	6055	6389	43
H(22)	7165	6308	4049	58
H(23)	7813	7113	4066	84
H(24)	7925	7630	4840	120
H(25)	7390	7388	5621	96
H(26A)	4734	6439	6115	55
H(26B)	5294	6880	6133	55
H(26C)	4745	6458	6141	55
H(26D)	5310	6894	6096	55
H(28)	5716	7265	5302	62
H(29)	5596	7443	4385	68

H(30)	4823	6978	3897	73
H(31)	4170	6335	4325	69
H(32)	4290	6158	5242	62
H(28A)	5565	7298	5244	58
H(29A)	5409	7386	4319	63
H(30A)	4740	6770	3873	69
H(31A)	4227	6065	4352	70
H(32A)	4384	5977	5277	63
H(34)	6974	4636	3454	39
H(38)	4868	2665	3764	52
H(39)	5055	1944	3156	66
H(40)	5878	2002	2542	65
H(41)	6544	2769	2551	51
H(42A)	6428	5378	3005	48
H(42B)	6452	4815	2659	48
H(42C)	6475	5361	2982	48
H(42D)	6412	4787	2659	48
H(44)	5505	4284	2492	85
H(45)	4419	4218	2508	103
H(46)	3832	4883	2976	112
H(47)	4332	5614	3427	106
H(48)	5419	5680	3411	95
H(44A)	5351	4353	2559	90
H(45A)	4305	4585	2420	101
H(46A)	3911	5430	2750	107
H(47A)	4564	6043	3218	103
H(48A)	5610	5810	3356	93
H(50)	9203	2111	3119	36
H(54)	10573	3096	5259	44
H(55)	10059	3913	5546	50
H(56)	9230	4281	5056	51
H(57)	8893	3837	4288	45
H(58A)	9797	2361	2316	45
H(58B)	9433	2867	2594	45
H(60)	10985	2276	2564	54
H(61)	11897	2799	2673	62

H(62)	11837	3757	2855	74
H(63)	10865	4192	2929	86
H(64)	9953	3669	2821	65
H(66)	11742	1791	5688	36
H(70)	12872	-436	4496	39
H(71)	13592	-877	5065	49
H(72)	13833	-491	5899	45
H(73)	13367	331	6176	40
H(74A)	12211	2605	5358	40
H(74B)	12751	2182	5532	40
H(76)	13450	1794	4864	66
H(77)	13906	1889	4016	79
H(78)	13423	2438	3360	83
H(79)	12485	2893	3552	73
H(80)	12029	2799	4399	52
H(82)	8272	4350	1826	31
H(86)	10326	5953	3075	42
H(87)	10659	5507	3869	46
H(88)	10195	4662	4135	44
H(89)	9469	4229	3590	38
H(90A)	7317	4884	1952	35
H(90B)	7603	4656	2502	35
H(92)	7976	5281	3168	39
H(93)	8118	6189	3493	47
H(94)	7912	6957	2940	65
H(95)	7564	6817	2061	69
H(96)	7422	5908	1735	52
H(98)	2073	5912	5685	67
H(100)	2313	7133	6793	85
H(102)	574	6711	6307	73
H(106)	1007	4758	5176	44
H(108)	2173	5058	3931	42
H(110)	1493	6349	4806	39
H(114)	1496	5162	6217	54
H(116)	266	4115	6932	58
H(118)	-347	5289	5885	46

H(122)	371	6271	4604	46
H(124)	-1199	7108	5016	56
H(126)	-252	6223	6137	52
H(98A)	2090	5962	5623	67
H(200)	2269	7040	6868	86
H(202)	556	6515	6408	74
H(206)	1733	6098	4801	43
H(208)	805	6909	3618	45
H(210)	-139	6290	4894	45
H(214)	1134	5170	4601	45
H(216)	1733	3708	5242	58
H(218)	1197	4977	6215	52
H(222)	-99	6573	5775	52
H(224)	-1436	5640	6492	53
H(226)	100	4892	5879	45

Table 6. Torsion angles [°] for 16157ds.

I(1)-O(1)-C(1)-O(2)	-3.5(8)
I(1)-O(1)-C(1)-C(2)	172.9(5)
C(4)-N(1)-C(2)-C(1)	-168.0(6)
I(3)-N(1)-C(2)-C(1)	24.3(8)
C(4)-N(1)-C(2)-C(3)	70.2(8)
I(3)-N(1)-C(2)-C(3)	-97.5(6)
O(2)-C(1)-C(2)-N(1)	-14.4(10)
O(1)-C(1)-C(2)-N(1)	169.0(6)
O(2)-C(1)-C(2)-C(3)	108.7(8)
O(1)-C(1)-C(2)-C(3)	-67.9(9)
N(1)-C(2)-C(3)-C(11A)	59.7(16)
C(1)-C(2)-C(3)-C(11A)	-62.4(16)
N(1)-C(2)-C(3)-C(11)	49.9(9)
C(1)-C(2)-C(3)-C(11)	-72.2(9)
C(2)-N(1)-C(4)-O(3)	11.2(11)
I(3)-N(1)-C(4)-O(3)	179.7(6)
C(2)-N(1)-C(4)-C(5)	-168.5(7)
I(3)-N(1)-C(4)-C(5)	0.1(8)
O(3)-C(4)-C(5)-C(10)	1.5(12)
N(1)-C(4)-C(5)-C(10)	-178.8(7)
O(3)-C(4)-C(5)-C(6)	-175.5(7)
N(1)-C(4)-C(5)-C(6)	4.1(10)
C(10)-C(5)-C(6)-C(7)	-1.3(12)
C(4)-C(5)-C(6)-C(7)	175.9(7)
C(10)-C(5)-C(6)-I(3)	176.8(6)
C(4)-C(5)-C(6)-I(3)	-6.1(9)
C(5)-C(6)-C(7)-C(8)	0.9(12)
I(3)-C(6)-C(7)-C(8)	-176.9(6)
C(6)-C(7)-C(8)-C(9)	-0.2(13)
C(7)-C(8)-C(9)-C(10)	-0.1(15)
C(8)-C(9)-C(10)-C(5)	-0.3(14)
C(6)-C(5)-C(10)-C(9)	1.0(12)
C(4)-C(5)-C(10)-C(9)	-176.1(8)
C(2)-C(3)-C(11)-C(12)	85.2(10)

C(2)-C(3)-C(11)-C(16)	-88.3(8)
C(16)-C(11)-C(12)-C(13)	0.0
C(3)-C(11)-C(12)-C(13)	-173.4(11)
C(11)-C(12)-C(13)-C(14)	0.0
C(12)-C(13)-C(14)-C(15)	0.0
C(13)-C(14)-C(15)-C(16)	0.0
C(14)-C(15)-C(16)-C(11)	0.0
C(12)-C(11)-C(16)-C(15)	0.0
C(3)-C(11)-C(16)-C(15)	173.4(11)
C(2)-C(3)-C(11A)-C(12A)	97(2)
C(2)-C(3)-C(11A)-C(16A)	-82.0(15)
C(16A)-C(11A)-C(12A)-C(13A)	0.0
C(3)-C(11A)-C(12A)-C(13A)	-179(3)
C(11A)-C(12A)-C(13A)-C(14A)	0.0
C(12A)-C(13A)-C(14A)-C(15A)	0.0
C(13A)-C(14A)-C(15A)-C(16A)	0.0
C(14A)-C(15A)-C(16A)-C(11A)	0.0
C(12A)-C(11A)-C(16A)-C(15A)	0.0
C(3)-C(11A)-C(16A)-C(15A)	179(2)
I(3)-O(4)-C(17)-O(5)	-1.4(9)
I(3)-O(4)-C(17)-C(18)	178.1(5)
C(19)-N(2)-C(18)-C(17)	-170.0(7)
I(2)-N(2)-C(18)-C(17)	24.7(8)
C(19)-N(2)-C(18)-C(26)	67.0(9)
I(2)-N(2)-C(18)-C(26)	-98.2(7)
O(5)-C(17)-C(18)-N(2)	-12.8(10)
O(4)-C(17)-C(18)-N(2)	167.7(6)
O(5)-C(17)-C(18)-C(26)	110.5(8)
O(4)-C(17)-C(18)-C(26)	-69.0(9)
C(18)-N(2)-C(19)-O(6)	14.8(13)
I(2)-N(2)-C(19)-O(6)	-179.0(7)
C(18)-N(2)-C(19)-C(20)	-161.7(7)
I(2)-N(2)-C(19)-C(20)	4.5(10)
O(6)-C(19)-C(20)-C(21)	-177.0(9)
N(2)-C(19)-C(20)-C(21)	-0.5(13)
O(6)-C(19)-C(20)-C(25)	-3.2(16)

N(2)-C(19)-C(20)-C(25)	173.3(10)
C(25)-C(20)-C(21)-C(22)	0.5(15)
C(19)-C(20)-C(21)-C(22)	174.5(9)
C(25)-C(20)-C(21)-I(2)	-177.7(9)
C(19)-C(20)-C(21)-I(2)	-3.7(11)
C(20)-C(21)-C(22)-C(23)	-1.3(15)
I(2)-C(21)-C(22)-C(23)	176.6(8)
C(21)-C(22)-C(23)-C(24)	1.4(19)
C(22)-C(23)-C(24)-C(25)	-1(2)
C(23)-C(24)-C(25)-C(20)	0(3)
C(21)-C(20)-C(25)-C(24)	0(2)
C(19)-C(20)-C(25)-C(24)	-173.4(13)
N(2)-C(18)-C(26)-C(27)	46.0(12)
C(17)-C(18)-C(26)-C(27)	-76.3(11)
N(2)-C(18)-C(26)-C(27A)	52(2)
C(17)-C(18)-C(26)-C(27A)	-71(2)
C(18)-C(26)-C(27)-C(28)	-87.1(9)
C(18)-C(26)-C(27)-C(32)	87.7(12)
C(32)-C(27)-C(28)-C(29)	0.0
C(26)-C(27)-C(28)-C(29)	174.8(14)
C(27)-C(28)-C(29)-C(30)	0.0
C(28)-C(29)-C(30)-C(31)	0.0
C(29)-C(30)-C(31)-C(32)	0.0
C(30)-C(31)-C(32)-C(27)	0.0
C(28)-C(27)-C(32)-C(31)	0.0
C(26)-C(27)-C(32)-C(31)	-174.7(14)
C(18)-C(26)-C(27A)-C(28A)	-98.5(17)
C(18)-C(26)-C(27A)-C(32A)	77(3)
C(32A)-C(27A)-C(28A)-C(29A)	0.0
C(26)-C(27A)-C(28A)-C(29A)	175(3)
C(27A)-C(28A)-C(29A)-C(30A)	0.0
C(28A)-C(29A)-C(30A)-C(31A)	0.0
C(29A)-C(30A)-C(31A)-C(32A)	0.0
C(30A)-C(31A)-C(32A)-C(27A)	0.0
C(28A)-C(27A)-C(32A)-C(31A)	0.0
C(26)-C(27A)-C(32A)-C(31A)	-175(3)

I(2)-O(7)-C(33)-O(8)	2.9(8)
I(2)-O(7)-C(33)-C(34)	-178.9(5)
C(35)-N(3)-C(34)-C(33)	-163.6(6)
I(1)-N(3)-C(34)-C(33)	21.1(8)
C(35)-N(3)-C(34)-C(42)	71.8(8)
I(1)-N(3)-C(34)-C(42)	-103.4(6)
O(8)-C(33)-C(34)-N(3)	-9.2(10)
O(7)-C(33)-C(34)-N(3)	172.5(6)
O(8)-C(33)-C(34)-C(42)	115.7(8)
O(7)-C(33)-C(34)-C(42)	-62.6(9)
Na(1)-O(9)-C(35)-N(3)	96.7(8)
Na(1)-O(9)-C(35)-C(36)	-85.3(8)
C(34)-N(3)-C(35)-O(9)	8.4(11)
I(1)-N(3)-C(35)-O(9)	-176.1(6)
C(34)-N(3)-C(35)-C(36)	-169.8(6)
I(1)-N(3)-C(35)-C(36)	5.7(8)
O(9)-C(35)-C(36)-C(41)	-3.0(12)
N(3)-C(35)-C(36)-C(41)	175.2(7)
O(9)-C(35)-C(36)-C(37)	-178.5(7)
N(3)-C(35)-C(36)-C(37)	-0.3(10)
C(41)-C(36)-C(37)-C(38)	1.5(12)
C(35)-C(36)-C(37)-C(38)	177.2(7)
C(41)-C(36)-C(37)-I(1)	179.3(6)
C(35)-C(36)-C(37)-I(1)	-5.1(9)
C(36)-C(37)-C(38)-C(39)	-1.7(12)
I(1)-C(37)-C(38)-C(39)	-179.2(7)
C(37)-C(38)-C(39)-C(40)	0.4(14)
C(38)-C(39)-C(40)-C(41)	1.2(16)
C(39)-C(40)-C(41)-C(36)	-1.5(14)
C(37)-C(36)-C(41)-C(40)	0.1(12)
C(35)-C(36)-C(41)-C(40)	-175.4(8)
N(3)-C(34)-C(42)-C(43A)	62(2)
C(33)-C(34)-C(42)-C(43A)	-63(2)
N(3)-C(34)-C(42)-C(43)	54.4(11)
C(33)-C(34)-C(42)-C(43)	-70.2(10)
C(34)-C(42)-C(43)-C(44)	-88.5(10)

C(34)-C(42)-C(43)-C(48)	84.8(11)
C(48)-C(43)-C(44)-C(45)	0.0
C(42)-C(43)-C(44)-C(45)	173.3(14)
C(43)-C(44)-C(45)-C(46)	0.0
C(44)-C(45)-C(46)-C(47)	0.0
C(45)-C(46)-C(47)-C(48)	0.0
C(46)-C(47)-C(48)-C(43)	0.0
C(44)-C(43)-C(48)-C(47)	0.0
C(42)-C(43)-C(48)-C(47)	-173.2(15)
C(34)-C(42)-C(43A)-C(44A)	-95(2)
C(34)-C(42)-C(43A)-C(48A)	96(2)
C(48A)-C(43A)-C(44A)-C(45A)	0.0
C(42)-C(43A)-C(44A)-C(45A)	-169(4)
C(43A)-C(44A)-C(45A)-C(46A)	0.0
C(44A)-C(45A)-C(46A)-C(47A)	0.0
C(45A)-C(46A)-C(47A)-C(48A)	0.0
C(46A)-C(47A)-C(48A)-C(43A)	0.0
C(44A)-C(43A)-C(48A)-C(47A)	0.0
C(42)-C(43A)-C(48A)-C(47A)	169(3)
I(6)#3-O(11)-C(49)-O(10)	-0.4(8)
I(6)#3-O(11)-C(49)-C(50)	-179.3(5)
C(51)-N(4)-C(50)-C(49)	-170.3(6)
I(4)-N(4)-C(50)-C(49)	9.4(8)
C(51)-N(4)-C(50)-C(58)	65.2(8)
I(4)-N(4)-C(50)-C(58)	-115.0(6)
O(10)-C(49)-C(50)-N(4)	-3.7(9)
O(11)-C(49)-C(50)-N(4)	175.2(6)
O(10)-C(49)-C(50)-C(58)	122.8(7)
O(11)-C(49)-C(50)-C(58)	-58.3(9)
C(50)-N(4)-C(51)-O(12)	4.0(10)
I(4)-N(4)-C(51)-O(12)	-175.8(5)
C(50)-N(4)-C(51)-C(52)	-176.0(6)
I(4)-N(4)-C(51)-C(52)	4.2(7)
O(12)-C(51)-C(52)-C(53)	178.1(7)
N(4)-C(51)-C(52)-C(53)	-1.9(9)
O(12)-C(51)-C(52)-C(57)	-4.2(11)

N(4)-C(51)-C(52)-C(57)	175.8(7)
C(57)-C(52)-C(53)-C(54)	0.3(11)
C(51)-C(52)-C(53)-C(54)	178.1(7)
C(57)-C(52)-C(53)-I(4)	-179.1(5)
C(51)-C(52)-C(53)-I(4)	-1.3(8)
C(52)-C(53)-C(54)-C(55)	-1.4(11)
I(4)-C(53)-C(54)-C(55)	177.9(6)
C(53)-C(54)-C(55)-C(56)	1.0(12)
C(54)-C(55)-C(56)-C(57)	0.6(13)
C(55)-C(56)-C(57)-C(52)	-1.8(12)
C(53)-C(52)-C(57)-C(56)	1.4(11)
C(51)-C(52)-C(57)-C(56)	-176.4(7)
N(4)-C(50)-C(58)-C(59)	46.7(8)
C(49)-C(50)-C(58)-C(59)	-77.9(7)
C(50)-C(58)-C(59)-C(60)	72.1(7)
C(50)-C(58)-C(59)-C(64)	-105.3(6)
C(64)-C(59)-C(60)-C(61)	0.0
C(58)-C(59)-C(60)-C(61)	-177.3(6)
C(59)-C(60)-C(61)-C(62)	0.0
C(60)-C(61)-C(62)-C(63)	0.0
C(61)-C(62)-C(63)-C(64)	0.0
C(62)-C(63)-C(64)-C(59)	0.0
C(60)-C(59)-C(64)-C(63)	0.0
C(58)-C(59)-C(64)-C(63)	177.5(6)
I(4)-O(14)-C(65)-O(13)	-3.8(8)
I(4)-O(14)-C(65)-C(66)	173.4(5)
C(67)-N(5)-C(66)-C(65)	-165.2(6)
I(5)-N(5)-C(66)-C(65)	13.4(8)
C(67)-N(5)-C(66)-C(74)	71.4(8)
I(5)-N(5)-C(66)-C(74)	-110.0(6)
O(13)-C(65)-C(66)-N(5)	-12.4(9)
O(14)-C(65)-C(66)-N(5)	170.3(6)
O(13)-C(65)-C(66)-C(74)	112.6(7)
O(14)-C(65)-C(66)-C(74)	-64.7(8)
Na(1)#4-O(15)-C(67)-N(5)	-159(2)
Na(1)#4-O(15)-C(67)-C(68)	21(3)

C(66)-N(5)-C(67)-O(15)	4.8(10)
I(5)-N(5)-C(67)-O(15)	-173.9(5)
C(66)-N(5)-C(67)-C(68)	-175.2(6)
I(5)-N(5)-C(67)-C(68)	6.1(7)
O(15)-C(67)-C(68)-C(69)	178.8(7)
N(5)-C(67)-C(68)-C(69)	-1.2(9)
O(15)-C(67)-C(68)-C(73)	-2.8(10)
N(5)-C(67)-C(68)-C(73)	177.2(6)
C(73)-C(68)-C(69)-C(70)	-0.9(10)
C(67)-C(68)-C(69)-C(70)	177.5(6)
C(73)-C(68)-C(69)-I(5)	177.4(5)
C(67)-C(68)-C(69)-I(5)	-4.2(8)
C(68)-C(69)-C(70)-C(71)	-0.3(11)
I(5)-C(69)-C(70)-C(71)	-178.3(6)
C(69)-C(70)-C(71)-C(72)	0.8(12)
C(70)-C(71)-C(72)-C(73)	-0.1(12)
C(71)-C(72)-C(73)-C(68)	-1.1(11)
C(69)-C(68)-C(73)-C(72)	1.6(10)
C(67)-C(68)-C(73)-C(72)	-176.8(7)
N(5)-C(66)-C(74)-C(75)	65.6(8)
C(65)-C(66)-C(74)-C(75)	-58.4(8)
C(66)-C(74)-C(75)-C(76)	-94.1(6)
C(66)-C(74)-C(75)-C(80)	88.3(7)
C(80)-C(75)-C(76)-C(77)	0.0
C(74)-C(75)-C(76)-C(77)	-177.6(5)
C(75)-C(76)-C(77)-C(78)	0.0
C(76)-C(77)-C(78)-C(79)	0.0
C(77)-C(78)-C(79)-C(80)	0.0
C(78)-C(79)-C(80)-C(75)	0.0
C(76)-C(75)-C(80)-C(79)	0.0
C(74)-C(75)-C(80)-C(79)	177.6(5)
I(5)-O(16)-C(81)-O(17)	-10.1(8)
I(5)-O(16)-C(81)-C(82)#3	166.0(5)
C(83)-N(6)-C(82)-C(81)#1	-173.2(6)
I(6)-N(6)-C(82)-C(81)#1	26.1(7)
C(83)-N(6)-C(82)-C(90)	66.1(8)

I(6)-N(6)-C(82)-C(90)	-94.6(6)
C(82)-N(6)-C(83)-O(18)	11.0(10)
I(6)-N(6)-C(83)-O(18)	173.1(5)
C(82)-N(6)-C(83)-C(84)	-168.4(6)
I(6)-N(6)-C(83)-C(84)	-6.3(7)
O(18)-C(83)-C(84)-C(89)	4.8(10)
N(6)-C(83)-C(84)-C(89)	-175.8(6)
O(18)-C(83)-C(84)-C(85)	-172.3(6)
N(6)-C(83)-C(84)-C(85)	7.1(9)
C(89)-C(84)-C(85)-C(86)	0.0(10)
C(83)-C(84)-C(85)-C(86)	177.1(7)
C(89)-C(84)-C(85)-I(6)	178.7(5)
C(83)-C(84)-C(85)-I(6)	-4.2(8)
C(84)-C(85)-C(86)-C(87)	-0.1(11)
I(6)-C(85)-C(86)-C(87)	-178.6(6)
C(85)-C(86)-C(87)-C(88)	-1.1(12)
C(86)-C(87)-C(88)-C(89)	2.4(12)
C(87)-C(88)-C(89)-C(84)	-2.5(11)
C(85)-C(84)-C(89)-C(88)	1.3(10)
C(83)-C(84)-C(89)-C(88)	-175.7(7)
N(6)-C(82)-C(90)-C(91)	50.1(7)
C(81)#1-C(82)-C(90)-C(91)	-71.0(7)
C(82)-C(90)-C(91)-C(92)	-91.9(6)
C(82)-C(90)-C(91)-C(96)	80.9(6)
C(96)-C(91)-C(92)-C(93)	0.0
C(90)-C(91)-C(92)-C(93)	172.9(5)
C(91)-C(92)-C(93)-C(94)	0.0
C(92)-C(93)-C(94)-C(95)	0.0
C(93)-C(94)-C(95)-C(96)	0.0
C(94)-C(95)-C(96)-C(91)	0.0
C(92)-C(91)-C(96)-C(95)	0.0
C(90)-C(91)-C(96)-C(95)	-172.7(5)
C(102)-C(97)-C(98)-C(99)	0.0
B(1)-C(97)-C(98)-C(99)	-176.7(14)
C(97)-C(98)-C(99)-C(100)	0.0
C(97)-C(98)-C(99)-C(103)	-174.6(19)

C(98)-C(99)-C(100)-C(101)	0.0
C(103)-C(99)-C(100)-C(101)	174.7(19)
C(99)-C(100)-C(101)-C(102)	0.0
C(99)-C(100)-C(101)-C(104)	163.5(16)
C(100)-C(101)-C(102)-C(97)	0.0
C(104)-C(101)-C(102)-C(97)	-163.7(16)
C(98)-C(97)-C(102)-C(101)	0.0
B(1)-C(97)-C(102)-C(101)	176.6(14)
C(98)-C(99)-C(103)-F(2)	-66.9(16)
C(100)-C(99)-C(103)-F(2)	118.5(14)
C(98)-C(99)-C(103)-F(1)	50.6(16)
C(100)-C(99)-C(103)-F(1)	-124.0(15)
C(98)-C(99)-C(103)-F(3)	172.3(13)
C(100)-C(99)-C(103)-F(3)	-2.3(18)
C(100)-C(101)-C(104)-F(6)	121.3(14)
C(102)-C(101)-C(104)-F(6)	-74.9(15)
C(100)-C(101)-C(104)-F(4)	5.0(19)
C(102)-C(101)-C(104)-F(4)	168.8(13)
C(100)-C(101)-C(104)-F(5)	-119.8(15)
C(102)-C(101)-C(104)-F(5)	44.0(19)
C(110)-C(105)-C(106)-C(107)	0.0
B(1)-C(105)-C(106)-C(107)	-177.1(8)
C(105)-C(106)-C(107)-C(108)	0.0
C(105)-C(106)-C(107)-C(111)	179.9(9)
C(106)-C(107)-C(108)-C(109)	0.0
C(111)-C(107)-C(108)-C(109)	-179.9(9)
C(107)-C(108)-C(109)-C(110)	0.0
C(107)-C(108)-C(109)-C(112)	179.0(9)
C(108)-C(109)-C(110)-C(105)	0.0
C(112)-C(109)-C(110)-C(105)	-179.0(9)
C(106)-C(105)-C(110)-C(109)	0.0
B(1)-C(105)-C(110)-C(109)	177.2(8)
C(108)-C(107)-C(111)-F(9)	134.3(8)
C(106)-C(107)-C(111)-F(9)	-45.6(11)
C(108)-C(107)-C(111)-F(7)	12.4(12)
C(106)-C(107)-C(111)-F(7)	-167.5(8)

C(108)-C(107)-C(111)-F(8)	-106.7(10)
C(106)-C(107)-C(111)-F(8)	73.5(11)
C(108)-C(109)-C(112)-F(10)	-4.3(12)
C(110)-C(109)-C(112)-F(10)	174.7(7)
C(108)-C(109)-C(112)-F(11)	-124.3(8)
C(110)-C(109)-C(112)-F(11)	54.8(10)
C(108)-C(109)-C(112)-F(12)	117.8(9)
C(110)-C(109)-C(112)-F(12)	-63.2(10)
C(118)-C(113)-C(114)-C(115)	0.0
B(1)-C(113)-C(114)-C(115)	179.7(8)
C(113)-C(114)-C(115)-C(116)	0.0
C(113)-C(114)-C(115)-C(119)	176.3(11)
C(114)-C(115)-C(116)-C(117)	0.0
C(119)-C(115)-C(116)-C(117)	-176.3(11)
C(115)-C(116)-C(117)-C(118)	0.0
C(115)-C(116)-C(117)-C(120)	-177.4(9)
C(116)-C(117)-C(118)-C(113)	0.0
C(120)-C(117)-C(118)-C(113)	177.5(9)
C(114)-C(113)-C(118)-C(117)	0.0
B(1)-C(113)-C(118)-C(117)	-179.7(9)
C(116)-C(115)-C(119)-F(13)	98.3(12)
C(114)-C(115)-C(119)-F(13)	-78.0(12)
C(116)-C(115)-C(119)-F(15)	-22.0(15)
C(114)-C(115)-C(119)-F(15)	161.7(9)
C(116)-C(115)-C(119)-F(14)	-139.7(10)
C(114)-C(115)-C(119)-F(14)	44.0(13)
C(118)-C(117)-C(120)-F(18)	-54.9(11)
C(116)-C(117)-C(120)-F(18)	122.6(9)
C(118)-C(117)-C(120)-F(17)	63.5(11)
C(116)-C(117)-C(120)-F(17)	-119.1(10)
C(118)-C(117)-C(120)-F(16)	-173.7(8)
C(116)-C(117)-C(120)-F(16)	3.7(12)
C(126)-C(121)-C(122)-C(123)	0.0
B(1)-C(121)-C(122)-C(123)	-174.0(9)
C(121)-C(122)-C(123)-C(124)	0.0
C(121)-C(122)-C(123)-C(127)	-176.2(9)

C(122)-C(123)-C(124)-C(125)	0.0
C(127)-C(123)-C(124)-C(125)	176.1(9)
C(123)-C(124)-C(125)-C(126)	0.0
C(123)-C(124)-C(125)-C(128)	-177.6(10)
C(124)-C(125)-C(126)-C(121)	0.0
C(128)-C(125)-C(126)-C(121)	177.6(10)
C(122)-C(121)-C(126)-C(125)	0.0
B(1)-C(121)-C(126)-C(125)	174.1(9)
C(122)-C(123)-C(127)-F(20)	84.8(11)
C(124)-C(123)-C(127)-F(20)	-91.3(10)
C(122)-C(123)-C(127)-F(19)	-36.7(11)
C(124)-C(123)-C(127)-F(19)	147.1(8)
C(122)-C(123)-C(127)-F(21)	-158.5(7)
C(124)-C(123)-C(127)-F(21)	25.3(12)
C(126)-C(125)-C(128)-F(23)	147.2(9)
C(124)-C(125)-C(128)-F(23)	-35.2(13)
C(126)-C(125)-C(128)-F(24)	-92.3(11)
C(124)-C(125)-C(128)-F(24)	85.3(11)
C(126)-C(125)-C(128)-F(22)	26.6(13)
C(124)-C(125)-C(128)-F(22)	-155.8(10)
C(106)-C(105)-B(1)-C(121)	-112.3(8)
C(110)-C(105)-B(1)-C(121)	70.6(9)
C(106)-C(105)-B(1)-C(113)	12.4(10)
C(110)-C(105)-B(1)-C(113)	-164.7(6)
C(106)-C(105)-B(1)-C(97)	126.8(9)
C(110)-C(105)-B(1)-C(97)	-50.3(11)
C(122)-C(121)-B(1)-C(105)	-15.6(11)
C(126)-C(121)-B(1)-C(105)	170.4(6)
C(122)-C(121)-B(1)-C(113)	-139.3(7)
C(126)-C(121)-B(1)-C(113)	46.6(10)
C(122)-C(121)-B(1)-C(97)	104.5(9)
C(126)-C(121)-B(1)-C(97)	-69.5(10)
C(114)-C(113)-B(1)-C(105)	67.4(9)
C(118)-C(113)-B(1)-C(105)	-112.9(8)
C(114)-C(113)-B(1)-C(121)	-166.8(7)
C(118)-C(113)-B(1)-C(121)	12.9(11)

C(114)-C(113)-B(1)-C(97)	-48.4(10)
C(118)-C(113)-B(1)-C(97)	131.3(7)
C(98)-C(97)-B(1)-C(105)	-24.6(13)
C(102)-C(97)-B(1)-C(105)	158.8(11)
C(98)-C(97)-B(1)-C(121)	-147.9(10)
C(102)-C(97)-B(1)-C(121)	35.4(14)
C(98)-C(97)-B(1)-C(113)	92.4(11)
C(102)-C(97)-B(1)-C(113)	-84.2(13)
C(202)-C(97A)-C(98A)-C(99A)	0.0
B(1A)-C(97A)-C(98A)-C(99A)	177.7(16)
C(97A)-C(98A)-C(99A)-C(200)	0.0
C(97A)-C(98A)-C(99A)-C(203)	175(2)
C(98A)-C(99A)-C(200)-C(201)	0.0
C(203)-C(99A)-C(200)-C(201)	-175(2)
C(99A)-C(200)-C(201)-C(202)	0.0
C(99A)-C(200)-C(201)-C(204)	-163.9(19)
C(200)-C(201)-C(202)-C(97A)	0.0
C(204)-C(201)-C(202)-C(97A)	163.8(19)
C(98A)-C(97A)-C(202)-C(201)	0.0
B(1A)-C(97A)-C(202)-C(201)	-177.6(16)
C(200)-C(99A)-C(203)-F(2A)	86.3(19)
C(98A)-C(99A)-C(203)-F(2A)	-88.6(17)
C(200)-C(99A)-C(203)-F(1A)	-154.6(16)
C(98A)-C(99A)-C(203)-F(1A)	31(2)
C(200)-C(99A)-C(203)-F(3A)	-36(2)
C(98A)-C(99A)-C(203)-F(3A)	148.8(16)
C(200)-C(201)-C(204)-F(4A)	14(2)
C(202)-C(201)-C(204)-F(4A)	-149.5(15)
C(200)-C(201)-C(204)-F(5A)	148.7(16)
C(202)-C(201)-C(204)-F(5A)	-15(2)
C(200)-C(201)-C(204)-F(6A)	-95.7(16)
C(202)-C(201)-C(204)-F(6A)	100.3(16)
C(210)-C(205)-C(206)-C(207)	0.0
B(1A)-C(205)-C(206)-C(207)	-179.6(9)
C(205)-C(206)-C(207)-C(208)	0.0
C(205)-C(206)-C(207)-C(211)	-172.1(10)

C(206)-C(207)-C(208)-C(209)	0.0
C(211)-C(207)-C(208)-C(209)	171.8(10)
C(207)-C(208)-C(209)-C(210)	0.0
C(207)-C(208)-C(209)-C(212)	179.9(11)
C(208)-C(209)-C(210)-C(205)	0.0
C(212)-C(209)-C(210)-C(205)	-179.9(10)
C(206)-C(205)-C(210)-C(209)	0.0
B(1A)-C(205)-C(210)-C(209)	179.6(10)
C(206)-C(207)-C(211)-F(8A)	-170.7(9)
C(208)-C(207)-C(211)-F(8A)	17.3(14)
C(206)-C(207)-C(211)-F(9A)	-48.1(13)
C(208)-C(207)-C(211)-F(9A)	139.9(10)
C(206)-C(207)-C(211)-F(7A)	69.7(11)
C(208)-C(207)-C(211)-F(7A)	-102.3(10)
C(208)-C(209)-C(212)-F(11A)	104.1(12)
C(210)-C(209)-C(212)-F(11A)	-76.0(13)
C(208)-C(209)-C(212)-F(10A)	-15.4(14)
C(210)-C(209)-C(212)-F(10A)	164.5(9)
C(208)-C(209)-C(212)-F(12A)	-134.6(10)
C(210)-C(209)-C(212)-F(12A)	45.3(13)
C(218)-C(213)-C(214)-C(215)	0.0
B(1A)-C(213)-C(214)-C(215)	-176.5(10)
C(213)-C(214)-C(215)-C(216)	0.0
C(213)-C(214)-C(215)-C(219)	-179.3(12)
C(214)-C(215)-C(216)-C(217)	0.0
C(219)-C(215)-C(216)-C(217)	179.3(11)
C(215)-C(216)-C(217)-C(218)	0.0
C(215)-C(216)-C(217)-C(220)	178.2(13)
C(216)-C(217)-C(218)-C(213)	0.0
C(220)-C(217)-C(218)-C(213)	-178.3(12)
C(214)-C(213)-C(218)-C(217)	0.0
B(1A)-C(213)-C(218)-C(217)	176.6(10)
C(216)-C(215)-C(219)-F(13A)	-161.7(9)
C(214)-C(215)-C(219)-F(13A)	17.5(15)
C(216)-C(215)-C(219)-F(15A)	-40.6(14)
C(214)-C(215)-C(219)-F(15A)	138.7(10)

C(216)-C(215)-C(219)-F(14A)	79.6(12)
C(214)-C(215)-C(219)-F(14A)	-101.1(11)
C(216)-C(217)-C(220)-F(18A)	4.0(17)
C(218)-C(217)-C(220)-F(18A)	-177.8(11)
C(216)-C(217)-C(220)-F(17A)	-117.5(12)
C(218)-C(217)-C(220)-F(17A)	60.7(14)
C(216)-C(217)-C(220)-F(16A)	125.7(12)
C(218)-C(217)-C(220)-F(16A)	-56.1(14)
C(226)-C(221)-C(222)-C(223)	0.0
B(1A)-C(221)-C(222)-C(223)	-174.1(10)
C(221)-C(222)-C(223)-C(224)	0.0
C(221)-C(222)-C(223)-C(227)	177.7(12)
C(222)-C(223)-C(224)-C(225)	0.0
C(227)-C(223)-C(224)-C(225)	-177.6(13)
C(223)-C(224)-C(225)-C(226)	0.0
C(223)-C(224)-C(225)-C(228)	-177.9(11)
C(224)-C(225)-C(226)-C(221)	0.0
C(228)-C(225)-C(226)-C(221)	177.9(12)
C(222)-C(221)-C(226)-C(225)	0.0
B(1A)-C(221)-C(226)-C(225)	174.0(10)
C(222)-C(223)-C(227)-F(20A)	173.5(10)
C(224)-C(223)-C(227)-F(20A)	-8.9(16)
C(222)-C(223)-C(227)-F(19A)	50.4(13)
C(224)-C(223)-C(227)-F(19A)	-131.9(11)
C(222)-C(223)-C(227)-F(21A)	-63.2(13)
C(224)-C(223)-C(227)-F(21A)	114.4(12)
C(226)-C(225)-C(228)-F(24A)	145.7(9)
C(224)-C(225)-C(228)-F(24A)	-36.4(14)
C(226)-C(225)-C(228)-F(23A)	-92.9(11)
C(224)-C(225)-C(228)-F(23A)	85.0(11)
C(226)-C(225)-C(228)-F(22A)	25.6(13)
C(224)-C(225)-C(228)-F(22A)	-156.5(9)
C(206)-C(205)-B(1A)-C(221)	-179.5(7)
C(210)-C(205)-B(1A)-C(221)	1.0(12)
C(206)-C(205)-B(1A)-C(213)	-58.8(10)
C(210)-C(205)-B(1A)-C(213)	121.7(9)

C(206)-C(205)-B(1A)-C(97A)	61.7(10)
C(210)-C(205)-B(1A)-C(97A)	-117.9(9)
C(222)-C(221)-B(1A)-C(205)	-58.1(11)
C(226)-C(221)-B(1A)-C(205)	127.8(9)
C(222)-C(221)-B(1A)-C(213)	-178.5(7)
C(226)-C(221)-B(1A)-C(213)	7.4(12)
C(222)-C(221)-B(1A)-C(97A)	61.1(12)
C(226)-C(221)-B(1A)-C(97A)	-113.0(11)
C(214)-C(213)-B(1A)-C(205)	-11.3(11)
C(218)-C(213)-B(1A)-C(205)	172.2(7)
C(214)-C(213)-B(1A)-C(221)	110.8(9)
C(218)-C(213)-B(1A)-C(221)	-65.7(11)
C(214)-C(213)-B(1A)-C(97A)	-130.5(10)
C(218)-C(213)-B(1A)-C(97A)	53.0(13)
C(98A)-C(97A)-B(1A)-C(205)	-74.5(14)
C(202)-C(97A)-B(1A)-C(205)	103.2(15)
C(98A)-C(97A)-B(1A)-C(221)	164.4(12)
C(202)-C(97A)-B(1A)-C(221)	-18.0(18)
C(98A)-C(97A)-B(1A)-C(213)	44.9(15)
C(202)-C(97A)-B(1A)-C(213)	-137.5(13)

Symmetry transformations used to generate equivalent atoms:

#1 -x+2,y+1/2,-z+1/2   #2 x-1/2,-y+1/2,-z+1   #3 -x+2,y-1/2,-z+1/2

#4 x+1/2,-y+1/2,-z+1

Computational experiment

Seven systems were investigated via DFT experiment: HIM monomer, HIM **1** (onformer **I**), HIM **1** (conformer **II**), lithium complex **2**, lithium complex **3**, sodium complex **4** and sodium complex **5.** All six systems were optimized and vibrational frequencies were obtained using ground state DFT calculations, with B3LYP method, 6-31G(d,p) basis set for all atoms except iodine, which was handled with LanL2DZ basis set. All vibrational calculations of optimized structures showed no imaginary frequencies, meaning that the structure obtained by the optimization calculations was a minimum. All DFT calculations for each system were performed in gas phase (no solvent) and any solvent molecules or counter ions were removed to handle the charge during calculations. Electrostatic potential map was calculated for HIM **1** conformer I.

Electrostatic potential map of HIM **1** conformer I

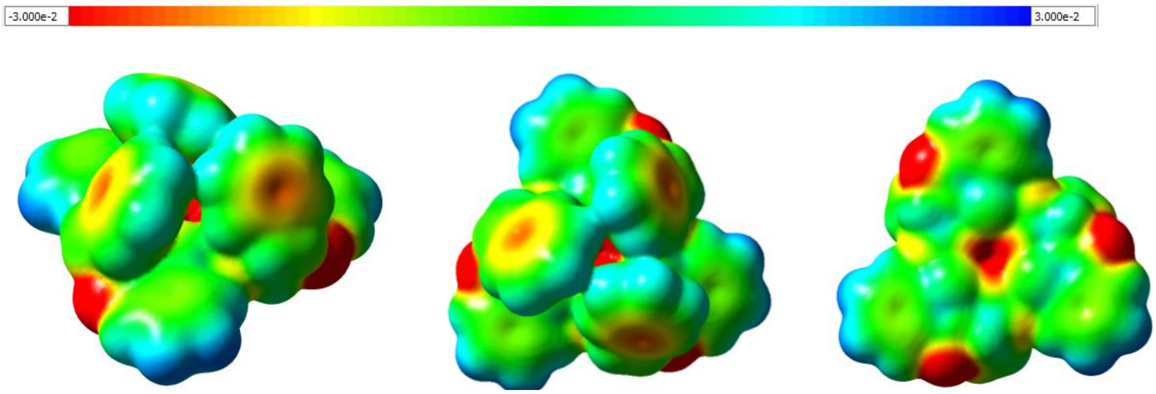

Figure S14. Calculated electrostatic potential map of HIM **1** conformer I. Left) side view, Middle) top view and right) bottom view. Calculated electrostatic potential map showing the electrophilic core highlighted by less intense red color surrounded by green color of **1**. Periphery oxygens (formerly the carbonyl of benzoic acid) are electron rich highlighted by more intense red color.

HIM monomer

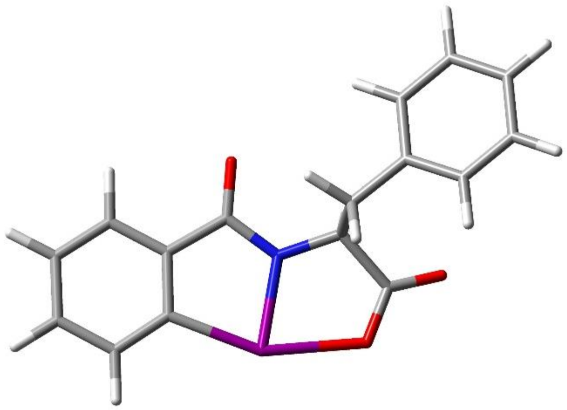

Figure S15. DFT image of HIM monomer (top view). Oxygen, nitrogen and the iodine atoms are denoted by red, blue and purple color respectively.

Coordinates for DFT calculated structure of hypervalent iodine macrocycle monomer

Job Type: geometry optimization and frequency calculation

Method: Ground state, Default spin, DFT, B3LYP

Basis Set: 6-31G(d,p) for all atoms excluding iodine, LanL2DZ for iodine

Solvent: none

Total Energy: -908.684607 Hartree

Number of Imaginary Frequencies: 0

Center Number	Atomic Number	X	Y	Z
1	53	1.604446	-1.452716	-0.122682
2	8	0.246205	2.402675	0.78083
3	8	-2.406277	-1.99831	1.266297
4	8	-0.398583	-2.438618	0.362116
5	7	0.344628	0.147412	0.480266
6	6	2.963842	0.219749	-0.197674
7	6	4.312078	0.146904	-0.52919
8	1	4.782097	-0.791721	-0.809413
9	6	5.062022	1.328695	-0.497398
10	1	6.116163	1.298368	-0.75707
11	6	4.465355	2.543513	-0.133854
12	1	5.061017	3.450811	-0.112351
13	6	3.112782	2.592764	0.201212
14	1	2.625957	3.519994	0.486111
15	6	2.355425	1.415154	0.171028
16	6	0.904222	1.400432	0.505134
17	6	-1.08166	-0.129317	0.555388

18	1	-1.459391	0.390669	1.441194
19	6	-1.345065	-1.640465	0.781402
20	6	-1.82146	0.370841	-0.725646
21	1	-1.355478	1.319781	-1.006501
22	1	-1.638371	-0.34165	-1.538714
23	6	-3.307326	0.588922	-0.539877
24	6	-3.766717	1.765358	0.073026
25	1	-3.047108	2.508777	0.408817
26	6	-5.13365	1.991075	0.249154
27	1	-5.472487	2.907517	0.724562
28	6	-6.062801	1.042705	-0.189774
29	1	-7.126647	1.216983	-0.054888
30	6	-5.614997	-0.130308	-0.803365
31	1	-6.329403	-0.874565	-1.143928
32	6	-4.246776	-0.354771	-0.97492
33	1	-3.906784	-1.276395	-1.439365

Conformer **I**

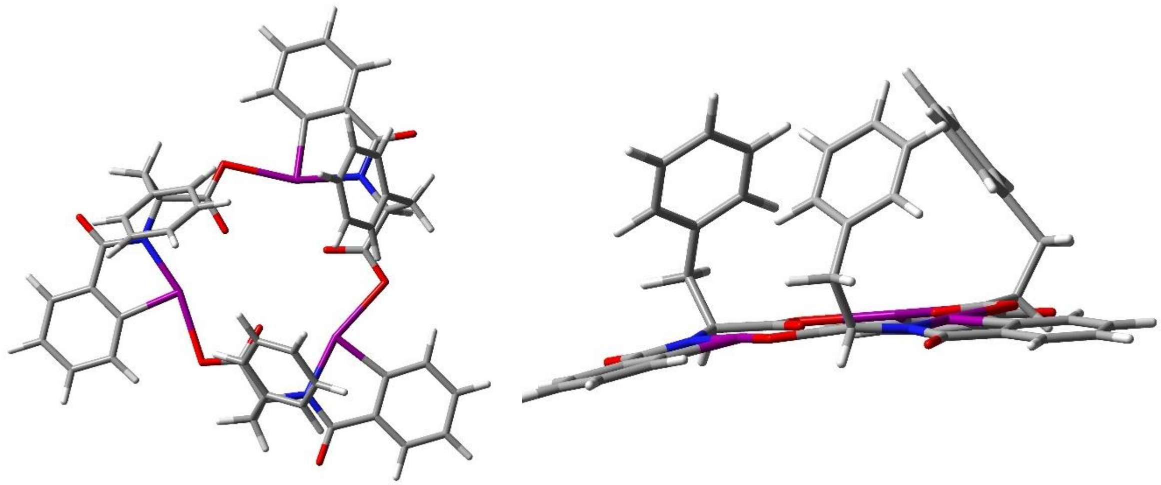

Figure S16. DFT image of Conformer **I** of HIM **1**. Left) top view. Right) side view. All three benzyl groups are facing towards the interior of the macrocycle. Oxygen, nitrogen and the iodine atoms are denoted by red, blue and purple color respectively.

Coordinates for DFT calculated structure of conformer I

Job Type: geometry optimization and frequency calculation

Method: Ground state, Default spin, DFT, B3LYP

Basis Set: 6-31G(d,p) for all atoms excluding iodine, LanL2DZ for iodine

Solvent: none

Total Energy: -2726.16696 Hartree

Number of Imaginary Frequencies: 0

Center Number	Atomic Number	X	Y	Z
1	53	-2.728482	-1.499153	-0.787294
2	53	0.065658	3.117924	-0.798232
3	53	2.658381	-1.617719	-0.792688
4	8	-6.585336	0.115706	-1.213798
5	8	-1.779635	0.867331	-0.726258
6	8	-2.398864	3.005984	-0.711331
7	8	3.384209	5.669636	-1.180423
8	8	3.798692	0.586862	-0.772671
9	8	1.637716	1.124011	-0.764865
10	8	3.209261	-5.767935	-1.167967
11	8	0.135609	-1.989054	-0.732187
12	8	-1.399426	-3.601539	-0.73509
13	7	-4.331986	-0.07836	-0.848768
14	7	2.095689	3.804171	-0.85136
15	7	2.239559	-3.71972	-0.83123

16	6	-4.412183	-2.788293	-1.051416
17	6	-4.352874	-4.175044	-1.117222
18	1	-3.408988	-4.696157	-1.012603
19	6	-5.552642	-4.863933	-1.332944
20	1	-5.536736	-5.948731	-1.387037
21	6	-6.762747	-4.172927	-1.482087
22	1	-7.682404	-4.724928	-1.651121
23	6	-6.789728	-2.780482	-1.416418
24	1	-7.709455	-2.21472	-1.528126
25	6	-5.598904	-2.075715	-1.197541
26	6	-5.577386	-0.586352	-1.100224
27	6	-4.144917	1.357948	-0.635589
28	1	-4.645072	1.878016	-1.461885
29	6	-2.661201	1.759185	-0.701649
30	6	-4.804109	1.86411	0.68683
31	1	-4.625697	2.94322	0.727847
32	1	-5.879494	1.708366	0.575529
33	6	-4.313463	1.19398	1.952242
34	6	-5.014919	0.107317	2.498647
35	1	-5.922929	-0.239101	2.01129
36	6	-4.573201	-0.520028	3.667404
37	1	-5.134923	-1.355064	4.077509
38	6	-3.418382	-0.067405	4.312066
39	1	-3.079375	-0.547322	5.226211
40	6	-2.711959	1.016494	3.781458
41	1	-1.814839	1.379493	4.274754
42	6	-3.158791	1.641961	2.614646
43	1	-2.611479	2.493134	2.218559
44	6	-0.215261	5.223107	-1.030454
45	6	-1.448598	5.859268	-1.093864
46	1	-2.366465	5.292033	-1.00182
47	6	-1.452109	7.245462	-1.29104
48	1	-2.402091	7.769673	-1.342968
49	6	-0.251416	7.95602	-1.424427
50	1	-0.274369	9.030558	-1.578994
51	6	0.971219	7.288508	-1.362865
52	1	1.918831	7.808253	-1.464629
53	6	0.99234	5.901962	-1.163259
54	6	2.273982	5.141896	-1.07724
55	6	3.248189	2.92242	-0.658938
56	1	3.942069	3.109002	-1.488046
57	6	2.851499	1.438923	-0.747465
58	6	4.02615	3.215906	0.663037
59	1	4.869957	2.519126	0.684692
60	1	4.430203	4.226136	0.567983

61	6	3.209697	3.101861	1.932663
62	6	2.639009	4.244465	2.515936
63	1	2.801083	5.215028	2.053578
64	6	1.884224	4.152738	3.689278
65	1	1.45712	5.050441	4.128237
66	6	1.688348	2.911221	4.301235
67	1	1.110408	2.83957	5.218703
68	6	2.254105	1.765282	3.733457
69	1	2.111956	0.795494	4.202041
70	6	3.009927	1.862263	2.562323
71	1	3.458399	0.968162	2.13758
72	6	4.619825	-2.424694	-1.053299
73	6	5.786133	-1.67414	-1.136727
74	1	5.758058	-0.595073	-1.047177
75	6	6.985906	-2.364628	-1.347014
76	1	7.913799	-1.803831	-1.414004
77	6	7.000212	-3.760302	-1.473913
78	1	7.940433	-4.277789	-1.638978
79	6	5.812414	-4.485873	-1.391668
80	1	5.788216	-5.56694	-1.487412
81	6	4.603532	-3.810771	-1.178145
82	6	3.306875	-4.54213	-1.069185
83	6	0.902761	-4.278982	-0.62327
84	1	0.711907	-4.979328	-1.445993
85	6	-0.190401	-3.199841	-0.710441
86	6	0.784626	-5.089496	0.706338
87	1	-0.240326	-5.471485	0.745279
88	1	1.456577	-5.945047	0.609843
89	6	1.112752	-4.312537	1.963423
90	6	2.397849	-4.379371	2.525197
91	1	3.151062	-5.008088	2.056796
92	6	2.714703	-3.664313	3.684128
93	1	3.713709	-3.734921	4.106024
94	6	1.746683	-2.868093	4.303132
95	1	1.988744	-2.318917	5.209066
96	6	0.461293	-2.79574	3.75728
97	1	-0.300705	-2.183042	4.23055
98	6	0.148445	-3.51439	2.600528
99	1	-0.857449	-3.46249	2.192579

Conformer **II**

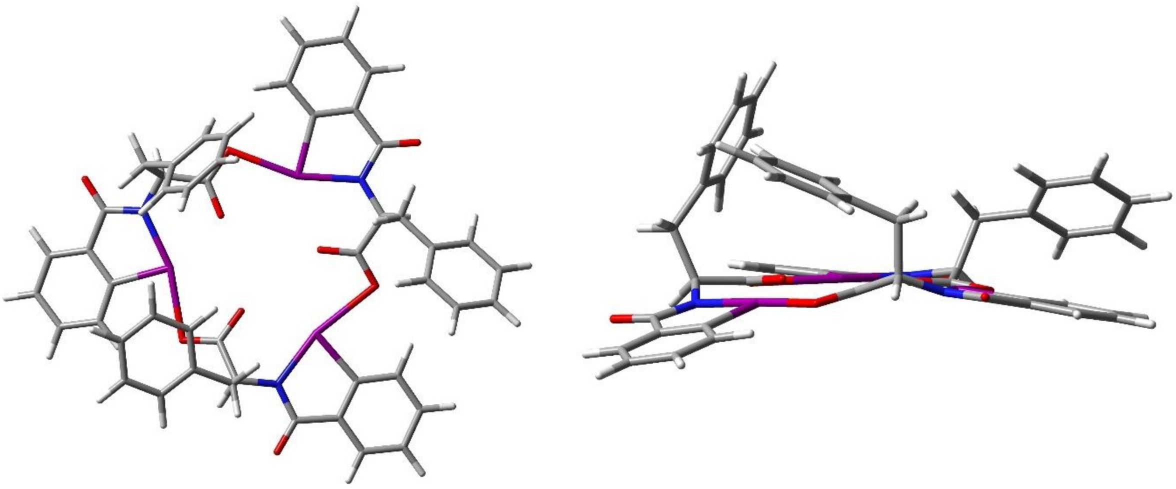

Figure S17. DFT image of conformer **II** of HIM **1**. Left) top view. Right) side view. Two benzyl groups are facing towards the interior of the macrocycle and one benzyl projected out. Oxygen, nitrogen and the iodine atoms are denoted by red, blue and purple color respectively.

Coordinates for DFT calculated structure of conformer II
Job Type: geometry optimization and frequency calculation
Method: Ground state, Default spin, DFT, B3LYP
Basis Set: 6-31G(d,p) for all atoms excluding iodine, LanL2DZ for iodine
Solvent: none
Total Energy: -2726.144207 Hartree
Number of Imaginary Frequencies: 0

Center Number	Atomic Number	X	Y	Z
1	53	-3.201781	-0.285166	-0.80361
2	8	-6.119285	2.690918	-1.362307
3	8	-1.22836	3.702926	-0.668506
4	8	-1.467844	1.503701	-0.391041
5	7	-4.12385	1.638562	-0.949935
6	6	-5.192511	-0.823018	-1.352018
7	6	-5.63932	-2.125016	-1.538819
8	1	-4.982435	-2.970643	-1.37197
9	6	-6.968284	-2.298293	-1.942971
10	1	-7.348071	-3.304849	-2.092592
11	6	-7.806376	-1.194776	-2.152364
12	1	-8.833926	-1.349583	-2.467335
13	6	-7.328576	0.100241	-1.955948
14	1	-7.954835	0.974632	-2.10325

15	6	-6.002131	0.29396	-1.551179
16	6	-5.449296	1.658304	-1.294556
17	6	-3.461912	2.840844	-0.442328
18	1	-3.729597	3.65563	-1.119382
19	6	-1.942955	2.655951	-0.51424
20	6	-3.958573	3.218816	1.011223
21	1	-4.436016	4.201098	0.937405
22	1	-4.760009	2.516466	1.253649
23	6	-2.975644	3.235563	2.168896
24	6	-1.906529	4.146763	2.215105
25	1	-1.683932	4.721891	1.322046
26	6	-1.077411	4.22409	3.335963
27	1	-0.257169	4.937223	3.349874
28	6	-1.303051	3.39297	4.438463
29	1	-0.665141	3.459405	5.315153
30	6	-2.34462	2.46301	4.394446
31	1	-2.51507	1.79411	5.233789
32	6	-3.171757	2.387369	3.268856
33	1	-3.957322	1.634053	3.23743
34	53	1.128488	2.831839	-0.612867
35	8	5.160101	3.977057	-0.964807
36	8	3.767761	-0.869343	-0.7994
37	8	1.905596	0.343747	-0.530913
38	7	3.272231	2.719221	-0.642988
39	6	1.658642	4.907425	-0.695357
40	6	0.770193	5.973955	-0.593561
41	1	-0.289864	5.819041	-0.429791
42	6	1.296685	7.267746	-0.693341
43	1	0.624959	8.11776	-0.614792
44	6	2.668022	7.474595	-0.888473
45	1	3.056408	8.485483	-0.967811
46	6	3.537395	6.38775	-0.96511
47	1	4.607998	6.513223	-1.09306
48	6	3.030282	5.086948	-0.860608
49	6	3.933656	3.897789	-0.850437
50	6	4.030705	1.481667	-0.446674
51	1	4.819941	1.454116	-1.203917
52	6	3.152904	0.229978	-0.625249
53	6	4.68386	1.473452	0.973872
54	1	5.089871	2.477895	1.125081
55	1	3.894254	1.320755	1.718443
56	6	5.791009	0.460251	1.163265
57	6	7.013033	0.616974	0.489775
58	1	7.153898	1.46935	-0.170979
59	6	8.050895	-0.299329	0.670882

60	1	8.99078	-0.160057	0.143448
61	6	7.88694	-1.386707	1.536065
62	1	8.696838	-2.096114	1.682559
63	6	6.67843	-1.546963	2.218969
64	1	6.542635	-2.383665	2.899095
65	6	5.640463	-0.629216	2.031297
66	1	4.702588	-0.761663	2.565678
67	53	1.758841	-2.481466	-0.535897
68	8	0.469281	-6.493469	-0.34669
69	8	-2.762624	-2.731586	-0.661499
70	8	-0.773425	-1.742084	-0.4553
71	7	0.473762	-4.20273	-0.393347
72	6	3.145951	-4.085969	-0.792171
73	6	4.503574	-3.908651	-1.00975
74	1	4.921571	-2.912798	-1.02523
75	6	5.286356	-5.05498	-1.188251
76	1	6.354135	-4.947173	-1.354499
77	6	4.705901	-6.329895	-1.151139
78	1	5.326313	-7.209588	-1.293461
79	6	3.336634	-6.475908	-0.930752
80	1	2.860537	-7.450757	-0.89135
81	6	2.540441	-5.338564	-0.749993
82	6	1.077272	-5.430062	-0.482091
83	6	-0.903236	-4.07326	0.079288
84	1	-1.473696	-4.897357	-0.355833
85	6	-1.516074	-2.752294	-0.394245
86	6	-0.9524	-4.153802	1.643729
87	1	-0.750887	-5.196943	1.90623
88	1	-0.11801	-3.552998	2.022435
89	6	-2.237588	-3.668496	2.274395
90	6	-3.336347	-4.521752	2.449948
91	1	-3.257914	-5.565559	2.154564
92	6	-4.529285	-4.045295	3.000096
93	1	-5.369985	-4.721296	3.130805
94	6	-4.643908	-2.704931	3.383193
95	1	-5.573156	-2.33745	3.809554
96	6	-3.553013	-1.846203	3.22053
97	1	-3.617556	-0.801392	3.517751
98	6	-2.360151	-2.328858	2.676489
99	1	-1.51653	-1.65358	2.554988

Lithium complex **2 (I & II)**

Lithium complex **2 (I & II)** represents the HIM/(Li)BARF complex consisting HIMs in two different molecular conformation (**I** & **II**)

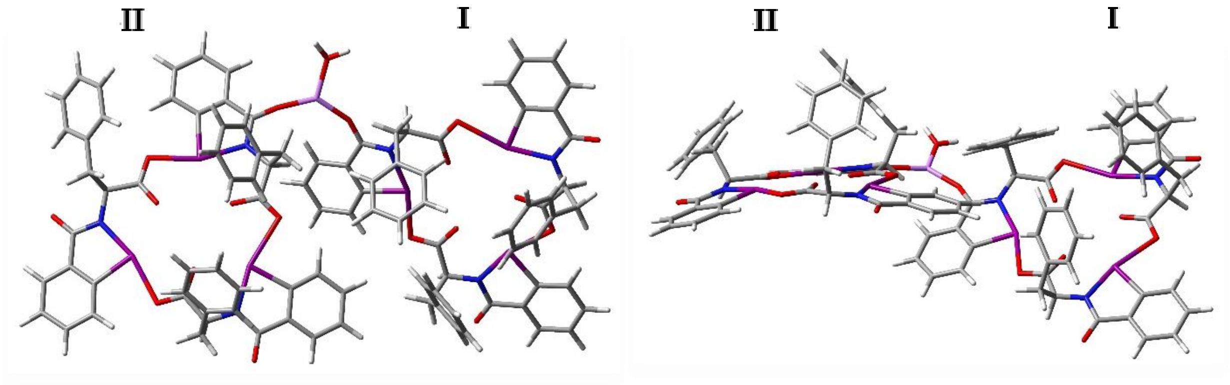

Figure S18. DFT image of lithium complex **2** (**I & II**) Left) top view. Right) side view. Two molecular conformers of HIM are present in Lithium complex **2** (**I & II**). Conformer **II** displays two benzyl groups are projected in and a benzyl group projected out. Conformer **I** displays all benzyl groups projected towards interior of the macrocycle. Lithium, Oxygen, nitrogen and the iodine atoms are denoted by lavender, red, blue and purple color respectively.

Coordinates for DFT calculated structure of lithium complex 2 (I & II)

Job Type: geometry optimization and frequency calculation

Method: Ground state, Default spin, DFT, B3LYP

Basis Set: 6-31G(d,p) for all atoms excluding iodine, LanL2DZ for iodine

Solvent: none

Total Energy: -5536.003845 Hartree

Number of Imaginary Frequencies: 0

Center Number	Atomic Number	X	Y	Z
1	53	-3.830464	-2.369209	0.88386
2	53	-5.883819	2.299414	-0.841653
3	53	-9.058812	-1.927444	-0.202836
4	53	3.749709	0.686584	-1.967777
5	53	8.342613	-2.057748	-1.781536
6	53	8.026243	2.724185	0.605642
7	8	-2.422377	-0.249571	0.614031
8	8	-4.497365	0.355696	0.062365
9	8	-2.257684	4.510955	-1.184644
10	8	-8.165424	2.327636	-1.44221
11	8	-7.920427	0.414808	-0.339873
12	8	-12.43173	-0.273397	-2.095321
13	8	-8.017257	-3.989041	0.624052
14	8	-6.358405	-2.504412	0.662564

15	8	-3.69279	-6.50917	1.539812
16	8	4.00373	-1.45017	-3.002329
17	8	5.980556	-1.021394	-2.088067
18	8	7.142447	-5.274127	-4.209093
19	8	10.20818	-1.035406	-0.550359
20	8	8.558882	0.448009	-0.371279
21	8	12.00737	3.091879	1.860023
22	8	5.752971	3.902279	0.490986
23	8	5.456583	2.019579	-0.667252
24	8	1.019703	3.900752	-1.424703
25	8	-0.039384	6.696956	-0.697227
26	1	-0.613507	7.363032	-0.301494
27	1	0.868847	7.005036	-0.59357
28	7	-3.767603	2.883912	-0.617846
29	7	-10.40381	-0.486506	-1.05682
30	7	-4.462051	-4.375317	1.245534
31	7	7.097405	-3.371112	-2.937131
32	7	10.05446	2.232376	1.036888
33	7	2.857128	2.558134	-1.241021
34	6	-3.284224	0.606365	0.249282
35	6	-2.788535	2.057213	0.087004
36	1	-1.88042	2.017307	-0.524777
37	6	-3.430766	4.049807	-1.175617
38	6	-4.562297	4.794976	-1.782334
39	6	-5.845033	4.246944	-1.733307
40	6	-6.957332	4.902507	-2.243766
41	1	-7.943335	4.459217	-2.186337
42	6	-6.760804	6.156383	-2.831785
43	1	-7.617699	6.688733	-3.232953
44	6	-5.484802	6.723799	-2.90685
45	1	-5.351007	7.694768	-3.373108
46	6	-4.386608	6.048053	-2.382805
47	1	-3.388387	6.470301	-2.427471
48	6	-2.405339	2.640083	1.481375
49	1	-1.616663	1.992643	1.877452
50	1	-1.968102	3.629122	1.315521
51	6	-3.548874	2.738356	2.467755
52	6	-4.249716	3.94015	2.636442
53	1	-3.951221	4.818554	2.068406
54	6	-5.316197	4.027239	3.533072
55	1	-5.843564	4.969015	3.655964
56	6	-5.698768	2.908494	4.275127
57	1	-6.526913	2.97537	4.974541
58	6	-5.005125	1.706306	4.120854
59	1	-5.293123	0.828876	4.692582

60	6	-3.936637	1.625198	3.228053
61	1	-3.394532	0.689833	3.120544
62	6	-8.62385	1.220944	-0.982071
63	6	-10.11078	0.938387	-1.201452
64	1	-10.37008	1.228811	-2.224378
65	6	-11.59017	-0.976696	-1.537578
66	6	-11.75828	-2.438906	-1.299066
67	6	-10.74853	-3.159877	-0.670608
68	6	-10.8323	-4.520082	-0.412194
69	1	-10.02104	-5.04898	0.072217
70	6	-12.00405	-5.174915	-0.807124
71	1	-12.10334	-6.239336	-0.616989
72	6	-13.03959	-4.478332	-1.439958
73	1	-13.93992	-5.005663	-1.738963
74	6	-12.92047	-3.113393	-1.688146
75	1	-13.70365	-2.543299	-2.177432
76	6	-10.96393	1.800978	-0.21399
77	1	-11.96044	1.352949	-0.213671
78	1	-10.55182	1.69903	0.795808
79	6	-11.06816	3.254557	-0.61623
80	6	-10.39322	4.261832	0.083456
81	1	-9.780503	3.998758	0.942281
82	6	-10.50747	5.59898	-0.303699
83	1	-9.987248	6.370072	0.257961
84	6	-11.29733	5.944809	-1.40173
85	1	-11.39445	6.98515	-1.699025
86	6	-11.97678	4.947128	-2.106008
87	1	-12.60308	5.209472	-2.95388
88	6	-11.86536	3.613575	-1.713823
89	1	-12.4059	2.838598	-2.252246
90	6	-6.800632	-3.664537	0.825047
91	6	-5.866777	-4.773102	1.327771
92	1	-6.000439	-5.635039	0.663872
93	6	-3.46831	-5.313149	1.368333
94	6	-2.098245	-4.731824	1.286338
95	6	-1.942717	-3.364958	1.088186
96	6	-0.699902	-2.754959	0.986833
97	1	-0.615598	-1.68935	0.813201
98	6	0.429699	-3.57147	1.106982
99	1	1.417938	-3.12817	1.033812
100	6	0.303489	-4.949893	1.314793
101	1	1.194842	-5.563074	1.402085
102	6	-0.957354	-5.533532	1.403257
103	1	-1.093255	-6.599082	1.558357
104	6	-6.288212	-5.223489	2.759904

105	1	-5.676424	-6.09524	3.001616
106	1	-7.327931	-5.55653	2.682897
107	6	-6.149328	-4.162659	3.82856
108	6	-4.979615	-4.072048	4.596931
109	1	-4.178785	-4.788554	4.433431
110	6	-4.843153	-3.092042	5.581182
111	1	-3.93375	-3.044828	6.173701
112	6	-5.880084	-2.186587	5.81692
113	1	-5.782962	-1.435821	6.596261
114	6	-7.052428	-2.268927	5.062058
115	1	-7.87033	-1.578658	5.248669
116	6	-7.183929	-3.24892	4.077351
117	1	-8.103944	-3.316785	3.502503
118	6	5.213024	-1.776773	-2.714423
119	6	5.663154	-3.178265	-3.139468
120	1	5.465584	-3.268657	-4.214676
121	6	7.722125	-4.455036	-3.497699
122	6	9.16651	-4.549878	-3.138039
123	6	9.741236	-3.588115	-2.31439
124	6	11.07401	-3.615062	-1.930082
125	1	11.48852	-2.843664	-1.293157
126	6	11.85479	-4.676163	-2.401041
127	1	12.9013	-4.728514	-2.116229
128	6	11.30586	-5.6613	-3.229599
129	1	11.9296	-6.475111	-3.585671
130	6	9.964919	-5.601735	-3.599835
131	1	9.507449	-6.34851	-4.240822
132	6	4.794821	-4.261477	-2.426359
133	1	3.752666	-4.038103	-2.676093
134	1	5.052761	-5.214818	-2.891324
135	6	4.980215	-4.338212	-0.927261
136	6	5.809596	-5.316859	-0.36239
137	1	6.30355	-6.034146	-1.012638
138	6	5.995288	-5.389806	1.019655
139	1	6.635206	-6.161522	1.437827
140	6	5.356268	-4.477938	1.861743
141	1	5.494557	-4.538716	2.937626
142	6	4.529416	-3.494342	1.312639
143	1	4.036552	-2.770962	1.956288
144	6	4.339961	-3.431275	-0.068624
145	1	3.684252	-2.672509	-0.487758
146	6	9.725214	0.063911	-0.123838
147	6	10.63616	0.917643	0.768982
148	1	11.56824	1.080165	0.214692
149	6	10.81488	3.228082	1.597042

150	6	10.04287	4.472324	1.874504
151	6	8.69152	4.528497	1.554106
152	6	7.905145	5.646759	1.793463
153	1	6.856105	5.658011	1.52538
154	6	8.522628	6.750403	2.391444
155	1	7.933913	7.639875	2.595697
156	6	9.881238	6.722114	2.726443
157	1	10.34137	7.590061	3.188108
158	6	10.64427	5.58625	2.469815
159	1	11.69951	5.529276	2.716855
160	6	11.00024	0.13927	2.069486
161	1	11.47169	-0.793525	1.74537
162	1	11.75631	0.733712	2.586279
163	6	9.831963	-0.147104	2.986457
164	6	9.549469	0.700028	4.067624
165	1	10.19264	1.556028	4.25498
166	6	8.471016	0.443697	4.915944
167	1	8.276325	1.104328	5.756161
168	6	7.655167	-0.668238	4.695161
169	1	6.82773	-0.879614	5.367192
170	6	7.924948	-1.52001	3.621266
171	1	7.30053	-2.39043	3.440713
172	6	9.00581	-1.261589	2.777851
173	1	9.220073	-1.937397	1.954382
174	6	5.038219	3.090935	-0.167846
175	6	3.544361	3.450679	-0.307783
176	1	3.500965	4.464261	-0.723831
177	6	1.63891	2.842785	-1.712921
178	6	1.062203	1.80208	-2.602697
179	6	1.806552	0.653039	-2.871567
180	6	1.333012	-0.367671	-3.685433
181	1	1.936972	-1.244281	-3.881315
182	6	0.058644	-0.223662	-4.242204
183	1	-0.332316	-1.010431	-4.880091
184	6	-0.708786	0.91738	-3.989166
185	1	-1.694279	1.015744	-4.433143
186	6	-0.211348	1.92978	-3.173329
187	1	-0.797754	2.81898	-2.967792
188	6	2.879666	3.488019	1.100702
189	1	3.443355	4.229153	1.675534
190	1	1.863079	3.867751	0.972973
191	6	2.847467	2.166673	1.838304
192	6	3.962426	1.718433	2.562081
193	1	4.8589	2.330755	2.594929
194	6	3.928383	0.505043	3.249468

195	1	4.805728	0.176976	3.799063
196	6	2.770613	-0.276646	3.233858
197	1	2.733534	-1.209656	3.789216
198	6	1.652468	0.15787	2.518633
199	1	0.742999	-0.435948	2.513896
200	6	1.695374	1.368292	1.824252
201	1	0.817383	1.705293	1.278171
202	3	-0.486978	4.896213	-1.160445

Lithium complex **3 (I & I)**

Lithium complex **3 (I & I)** represents the HIM/(Li)BARF complex consisting HIMs in same molecular conformation (**I** & **I**)

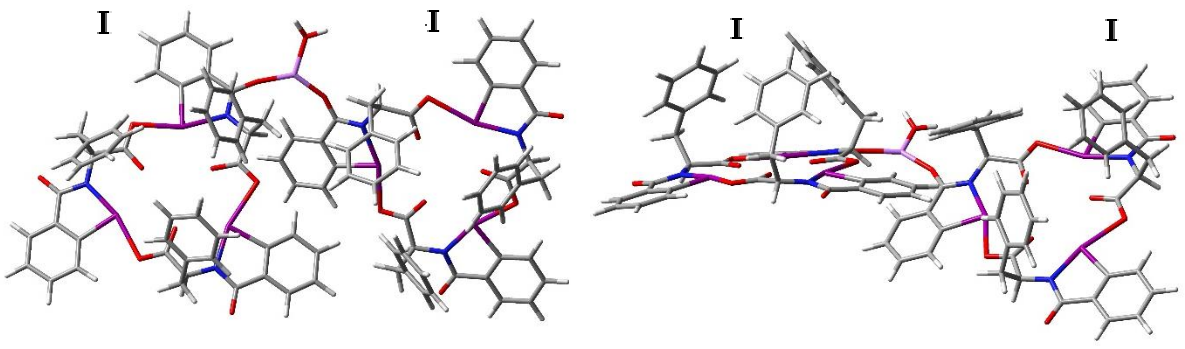

Figure S19. DFT image of lithium complex **3** (**I & I**) Left) top view. Right) side view. Both HIMs are present in same conformation, conformers **I** and **I**. All three benzyl groups are facing towards the interior of the macrocycle in both HIMs. Lithium, Oxygen, nitrogen and the iodine atoms are denoted by lavender, red, blue and purple color respectively.

Coordinates for DFT calculated structure of lithium complex 3 (I & I)
Job Type: geometry optimization and frequency calculation
Method: Ground state, Default spin, DFT, B3LYP
Basis Set: 6-31G(d,p) for all atoms excluding iodine, LanL2DZ for iodine
Solvent: none
Total Energy: -5536.004156 Hartree
Number of Imaginary Frequencies: 0

Center Number	Atomic Number	X	Y	Z
1	53	-3.948323	-2.109641	0.690666
2	53	-5.79046	2.539766	-1.324644
3	53	-9.122327	-1.571517	-0.571189
4	53	3.807807	0.532201	-2.028436
5	53	8.310236	-2.313211	-1.505742
6	53	8.050018	2.61898	0.562187
7	8	-2.462193	-0.048146	0.329593
8	8	-4.493168	0.592828	-0.334743
9	8	-2.094489	4.625702	-1.686054
10	8	-8.103883	2.667172	-1.86142
11	8	-7.82471	0.635103	-1.013791
12	8	-12.49767	0.161908	-2.377632
13	8	-8.170491	-3.622637	0.402848
14	8	-6.447336	-2.216855	0.303298

15	8	-3.963556	-6.167215	1.744662
16	8	4.033712	-1.671697	-2.914587
17	8	5.986581	-1.240288	-1.951761
18	8	7.100636	-5.645495	-3.766324
19	8	10.16206	-1.261639	-0.278898
20	8	8.552188	0.274711	-0.254223
21	8	11.99255	2.960593	1.939725
22	8	5.818104	3.851055	0.284967
23	8	5.507982	1.904262	-0.75811
24	8	1.163086	3.855071	-1.807928
25	8	0.218692	6.74359	-1.36898
26	1	-0.323089	7.469757	-1.038793
27	1	1.140886	7.012475	-1.279979
28	7	-3.663394	3.065916	-1.09167
29	7	-10.42085	-0.109872	-1.453947
30	7	-4.65317	-4.05676	1.196264
31	7	7.06749	-3.664448	-2.619348
32	7	10.04597	2.101043	1.101708
33	7	2.945505	2.471395	-1.457705
34	6	-3.279	0.812794	-0.113895
35	6	-2.722267	2.232597	-0.344312
36	1	-1.81783	2.119548	-0.952953
37	6	-3.281698	4.203857	-1.678695
38	6	-4.380298	4.964907	-2.323924
39	6	-5.679401	4.457742	-2.275438
40	6	-6.758384	5.122372	-2.84267
41	1	-7.754599	4.701619	-2.796413
42	6	-6.514154	6.345114	-3.476047
43	1	-7.343951	6.883865	-3.923225
44	6	-5.221179	6.874144	-3.541787
45	1	-5.049311	7.821495	-4.042704
46	6	-4.154909	6.187647	-2.968622
47	1	-3.14334	6.577045	-3.010514
48	6	-2.306978	2.866779	1.017206
49	1	-1.561719	2.192967	1.451004
50	1	-1.807573	3.815366	0.80037
51	6	-3.439239	3.093935	1.995322
52	6	-4.048417	4.351337	2.106525
53	1	-3.693219	5.174635	1.490639
54	6	-5.090561	4.564985	3.010339
55	1	-5.543703	5.548942	3.090941
56	6	-5.540295	3.518354	3.81767
57	1	-6.34089	3.687121	4.532208
58	6	-4.94372	2.259503	3.715078
59	1	-5.286788	1.436547	4.335032

60	6	-3.899043	2.052319	2.814489
61	1	-3.428603	1.075213	2.751015
62	6	-8.555741	1.518381	-1.500817
63	6	-10.0617	1.291768	-1.661374
64	1	-10.31967	1.547979	-2.696037
65	6	-11.663	-0.554393	-1.827675
66	6	-11.90303	-1.984438	-1.478271
67	6	-10.90695	-2.720836	-0.846526
68	6	-11.05993	-4.050817	-0.482659
69	1	-10.25818	-4.594465	0.001334
70	6	-12.28865	-4.655421	-0.769158
71	1	-12.44256	-5.694513	-0.494093
72	6	-13.31218	-3.941098	-1.402024
73	1	-14.25745	-4.429556	-1.616531
74	6	-13.12359	-2.608229	-1.757932
75	1	-13.89578	-2.025208	-2.249503
76	6	-10.85682	2.266989	-0.737814
77	1	-11.90755	2.158207	-1.014166
78	1	-10.54331	3.279988	-1.009667
79	6	-10.66929	2.029702	0.743623
80	6	-11.58322	1.244477	1.460757
81	1	-12.44568	0.828416	0.946573
82	6	-11.40879	1.009036	2.825428
83	1	-12.13282	0.40594	3.365866
84	6	-10.31358	1.557034	3.496484
85	1	-10.1845	1.384997	4.561465
86	6	-9.397561	2.343716	2.794747
87	1	-8.543342	2.778339	3.305608
88	6	-9.577672	2.57937	1.431572
89	1	-8.869418	3.206411	0.895996
90	6	-6.945864	-3.329299	0.593417
91	6	-6.072374	-4.404126	1.253954
92	1	-6.203503	-5.32721	0.677315
93	6	-3.695031	-5.000456	1.468218
94	6	-2.306324	-4.462686	1.405793
95	6	-2.102233	-3.124052	1.092645
96	6	-0.838657	-2.556449	1.000983
97	1	-0.715334	-1.51346	0.736798
98	6	0.259708	-3.385551	1.252682
99	1	1.262687	-2.974893	1.189234
100	6	0.083718	-4.735403	1.578577
101	1	0.951639	-5.359565	1.766375
102	6	-1.196312	-5.277449	1.655096
103	1	-1.370375	-6.320388	1.899827
104	6	-6.57191	-4.688452	2.702951

105	1	-5.995073	-5.540628	3.06847
106	1	-7.614722	-5.00721	2.609037
107	6	-6.459457	-3.520043	3.657038
108	6	-5.355252	-3.400177	4.512892
109	1	-4.58672	-4.168886	4.501091
110	6	-5.244604	-2.321943	5.39257
111	1	-4.3881	-2.253382	6.057453
112	6	-6.240049	-1.343004	5.429504
113	1	-6.163778	-0.513579	6.127419
114	6	-7.345448	-1.450019	4.582038
115	1	-8.128258	-0.69708	4.603046
116	6	-7.453582	-2.531389	3.707248
117	1	-8.324053	-2.617187	3.062518
118	6	5.221665	-2.012202	-2.560842
119	6	5.646282	-3.448589	-2.881848
120	1	5.48096	-3.602443	-3.955239
121	6	7.679646	-4.797789	-3.088774
122	6	9.108567	-4.906232	-2.675334
123	6	9.68288	-3.908918	-1.894822
124	6	11.00134	-3.945172	-1.464696
125	1	11.41617	-3.145665	-0.863634
126	6	11.76736	-5.053598	-1.8415
127	1	12.80233	-5.114341	-1.518731
128	6	11.21842	-6.07517	-2.624687
129	1	11.83065	-6.925583	-2.907706
130	6	9.892196	-6.005262	-3.043002
131	1	9.43518	-6.779486	-3.650894
132	6	4.725304	-4.46102	-2.131519
133	1	3.698314	-4.226445	-2.429395
134	1	4.970402	-5.448435	-2.527104
135	6	4.861024	-4.447584	-0.625196
136	6	5.63683	-5.415687	0.027185
137	1	6.125014	-6.189085	-0.560081
138	6	5.776667	-5.40672	1.4165
139	1	6.375181	-6.171412	1.903254
140	6	5.145057	-4.421758	2.177947
141	1	5.247877	-4.418128	3.259508
142	6	4.371566	-3.447712	1.541029
143	1	3.885959	-2.668047	2.121386
144	6	4.227277	-3.466564	0.15305
145	1	3.612053	-2.714486	-0.334059
146	6	9.697248	-0.124539	0.059708
147	6	10.6029	0.759974	0.927947
148	1	11.55363	0.867902	0.392051
149	6	10.81364	3.109957	1.627211

150	6	10.06674	4.387664	1.80208
151	6	8.730178	4.459277	1.427473
152	6	7.967066	5.609641	1.570893
153	1	6.929341	5.631988	1.262537
154	6	8.592765	6.730689	2.12646
155	1	8.021994	7.645559	2.255704
156	6	9.936895	6.687555	2.514127
157	1	10.40385	7.569335	2.941458
158	6	10.67685	5.519246	2.353608
159	1	11.72039	5.450028	2.643482
160	6	10.9129	0.052151	2.281672
161	1	11.3671	-0.909938	2.025864
162	1	11.67124	0.657262	2.782362
163	6	9.715074	-0.149222	3.182986
164	6	9.429135	0.768023	4.204352
165	1	10.09028	1.617174	4.357076
166	6	8.324224	0.589867	5.03856
167	1	8.127052	1.304148	5.833069
168	6	7.484739	-0.512539	4.863148
169	1	6.635967	-0.662574	5.524991
170	6	7.75773	-1.433603	3.848906
171	1	7.11497	-2.297375	3.704386
172	6	8.865163	-1.253384	3.019808
173	1	9.081705	-1.982666	2.243958
174	6	5.103292	3.016484	-0.343952
175	6	3.624908	3.403174	-0.557772
176	1	3.621243	4.389588	-1.036457
177	6	1.758355	2.761885	-1.999605
178	6	1.184141	1.682518	-2.84372
179	6	1.89976	0.495587	-3.004512
180	6	1.424665	-0.562683	-3.767974
181	1	2.006148	-1.468721	-3.879994
182	6	0.179029	-0.417438	-4.386077
183	1	-0.212701	-1.233288	-4.985796
184	6	-0.558619	0.76168	-4.242694
185	1	-1.521371	0.860458	-4.733934
186	6	-0.060011	1.810988	-3.475688
187	1	-0.62248	2.730865	-3.356117
188	6	2.914281	3.543075	0.821456
189	1	3.463803	4.32	1.361676
190	1	1.905436	3.918629	0.632985
191	6	2.848882	2.278128	1.650368
192	6	3.929284	1.891197	2.45726
193	1	4.821587	2.509791	2.485256
194	6	3.866228	0.731608	3.230032

195	1	4.717355	0.449215	3.842598
196	6	2.712516	-0.055961	3.217636
197	1	2.651386	-0.945473	3.838377
198	6	1.628756	0.317454	2.419821
199	1	0.722542	-0.281329	2.416626
200	6	1.701096	1.473556	1.640416
201	1	0.849616	1.762925	1.029031
202	3	-0.305417	4.928561	-1.666398

Sodium complex **4 (I & II)**

Sodium complex **4 (I & II)** represents the HIM/(Na)BARF complex consisting HIMs in two different molecular conformation (**I** & **II**)

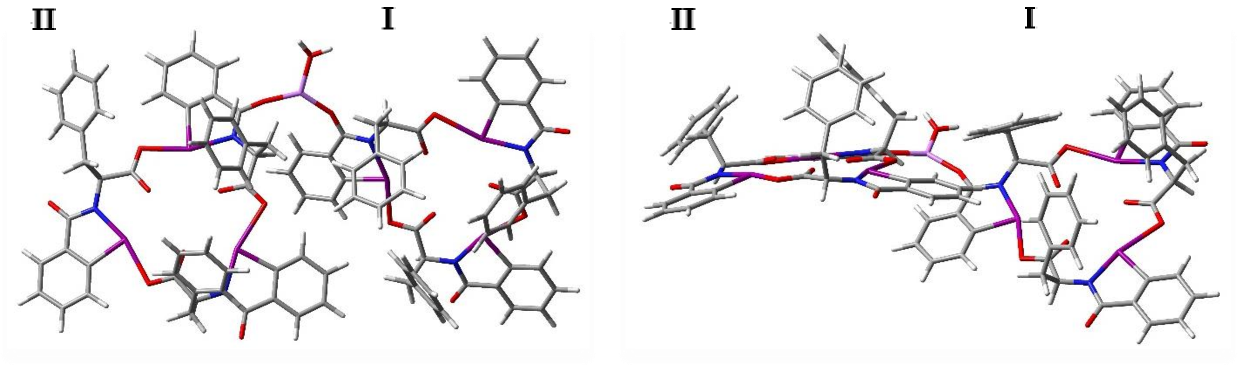

Figure S20. DFT image of sodium complex **4** (**I & II**) Left) top view. Right) side view. Two molecular conformers of HIM are present in sodium complex **4** (**I & II**). Conformer **II** displays two benzyl groups are projected in and a benzyl group projected out. Conformer **I** displays all benzyl groups projected towards interior of the macrocycle. Sodium, Oxygen, nitrogen and the iodine atoms are denoted by lavender, red, blue and purple color respectively.

Two benzyl groups are facing towards the interior of the macrocycle and one benzyl projected out. Lithium, Oxygen, nitrogen and the iodine atoms are denoted by lavender, red, blue and purple color respectively.

Coordinates for DFT calculated structure of sodium complex 4 (I & II)
Job Type: geometry optimization and frequency calculation
Method: Ground state, Default spin, DFT, B3LYP
Basis Set: 6-31G(d,p) for all atoms excluding iodine, LanL2DZ for iodine
Solvent: none
Total Energy: -5690.730237 Hartree
Number of Imaginary Frequencies: 0

Center Number	Atomic Number	X	Y	Z
1	53	-4.517301	-2.559772	0.830929
2	53	-6.21573	2.274266	-0.824547
3	53	-9.680844	-1.751094	-0.303835
4	53	4.241217	0.485144	-1.918864
5	53	9.000012	-2.004393	-1.69824
6	53	8.401254	2.753505	0.637851
7	8	-2.974762	-0.527886	0.618563
8	8	-4.997251	0.221498	0.048744
9	8	-2.446835	4.242447	-1.088046
10	8	-8.487467	2.458316	-1.446605
11	8	-8.383761	0.51168	-0.37918
12	8	-12.9094	0.165679	-2.199625

13	8	-8.792174	-3.895183	0.491802
14	8	-7.038629	-2.526357	0.580426
15	8	-4.650756	-6.712295	1.402413
16	8	4.636971	-1.64463	-2.934596
17	8	6.584031	-1.099376	-2.014846
18	8	7.982314	-5.29871	-4.103692
19	8	10.80414	-0.872993	-0.471425
20	8	9.07296	0.515254	-0.309667
21	8	12.34458	3.378229	1.911758
22	8	6.065758	3.782178	0.4948
23	8	5.883995	1.882353	-0.659788
24	8	1.332967	3.529169	-1.422048
25	8	0.121241	6.716355	-0.561247
26	1	-0.42967	7.400849	-0.159553
27	1	1.026866	7.041244	-0.47117
28	7	-4.086127	2.716581	-0.565991
29	7	-10.91392	-0.205567	-1.142823
30	7	-5.279016	-4.528124	1.144255
31	7	7.831047	-3.391658	-2.845901
32	7	10.45323	2.392369	1.085871
33	7	3.247434	2.275149	-1.236672
34	6	-3.774129	0.389374	0.259531
35	6	-3.181737	1.807998	0.137734
36	1	-2.267347	1.717642	-0.459438
37	6	-3.655311	3.86669	-1.09688
38	6	-4.731002	4.695306	-1.702242
39	6	-6.046718	4.231363	-1.676834
40	6	-7.109815	4.96566	-2.185218
41	1	-8.122452	4.585196	-2.145923
42	6	-6.827353	6.21484	-2.747276
43	1	-7.643919	6.808498	-3.146526
44	6	-5.51677	6.699979	-2.799249
45	1	-5.315966	7.668854	-3.245585
46	6	-4.469926	5.945217	-2.277528
47	1	-3.446721	6.304501	-2.304335
48	6	-2.787558	2.330651	1.553339
49	1	-2.052384	1.622382	1.94855
50	1	-2.282468	3.292346	1.421905
51	6	-3.940978	2.483484	2.520911
52	6	-4.562867	3.72556	2.706536
53	1	-4.19546	4.594978	2.165539
54	6	-5.638419	3.863337	3.585748
55	1	-6.103886	4.835355	3.722257
56	6	-6.109463	2.755788	4.292862
57	1	-6.944833	2.861907	4.978677

58	6	-5.495067	1.513494	4.121363
59	1	-5.85235	0.644282	4.665871
60	6	-4.417135	1.381404	3.246234
61	1	-3.936953	0.414362	3.125346
62	6	-9.023109	1.375877	-1.013044
63	6	-10.52297	1.198573	-1.254753
64	1	-10.74914	1.525849	-2.274385
65	6	-12.12459	-0.604185	-1.647038
66	6	-12.39456	-2.056007	-1.440915
67	6	-11.44408	-2.85637	-0.816204
68	6	-11.62315	-4.212652	-0.587186
69	1	-10.85582	-4.805156	-0.104826
70	6	-12.83146	-4.778232	-1.00893
71	1	-13.00508	-5.837038	-0.842185
72	6	-13.80929	-4.000289	-1.638783
73	1	-14.73942	-4.459067	-1.958901
74	6	-13.59476	-2.641926	-1.857112
75	1	-14.33119	-2.010184	-2.343279
76	6	-11.32844	2.09739	-0.259567
77	1	-12.35285	1.717956	-0.279597
78	1	-10.93692	1.948003	0.752512
79	6	-11.32944	3.562312	-0.63313
80	6	-10.59703	4.507723	0.094505
81	1	-10.01422	4.186994	0.954548
82	6	-10.61608	5.85688	-0.266484
83	1	-10.05215	6.579857	0.316588
84	6	-11.36701	6.276829	-1.36598
85	1	-11.39007	7.326994	-1.643027
86	6	-12.10323	5.341385	-2.098058
87	1	-12.69974	5.66207	-2.947358
88	6	-12.08681	3.995906	-1.731994
89	1	-12.67152	3.269961	-2.292173
90	6	-7.559325	-3.657331	0.713425
91	6	-6.707278	-4.835377	1.202982
92	1	-6.888858	-5.673061	0.519702
93	6	-4.348611	-5.529846	1.258265
94	6	-2.943509	-5.035393	1.203555
95	6	-2.699302	-3.67745	1.035725
96	6	-1.419269	-3.145631	0.961418
97	1	-1.265555	-2.084124	0.811477
98	6	-0.345163	-4.03446	1.077398
99	1	0.669997	-3.653428	1.024905
100	6	-0.56093	-5.406028	1.254717
101	1	0.288697	-6.076177	1.339316
102	6	-1.857187	-5.910133	1.316574

103	1	-2.062149	-6.967813	1.447851
104	6	-7.17488	-5.285963	2.620637
105	1	-6.624522	-6.200741	2.850706
106	1	-8.233147	-5.548311	2.52524
107	6	-6.980006	-4.258413	3.712825
108	6	-5.816469	-4.260543	4.495783
109	1	-5.06236	-5.02472	4.326082
110	6	-5.628283	-3.311826	5.501922
111	1	-4.725159	-3.336602	6.105318
112	6	-6.606495	-2.345109	5.745248
113	1	-6.470071	-1.61836	6.541301
114	6	-7.772276	-2.334813	4.97592
115	1	-8.545451	-1.596187	5.168008
116	6	-7.955497	-3.283826	3.969378
117	1	-8.870782	-3.279343	3.383107
118	6	5.861257	-1.899982	-2.638591
119	6	6.389077	-3.277865	-3.052495
120	1	6.199839	-3.38633	-4.127511
121	6	8.514952	-4.444133	-3.397247
122	6	9.96156	-4.457801	-3.034219
123	6	10.48121	-3.460152	-2.216751
124	6	11.81263	-3.411668	-1.829777
125	1	12.18307	-2.614072	-1.197946
126	6	12.65106	-4.432163	-2.291282
127	1	13.69823	-4.425379	-2.00416
128	6	12.1584	-5.45182	-3.113434
129	1	12.8263	-6.233097	-3.46223
130	6	10.81704	-5.468046	-3.486624
131	1	10.40235	-6.243325	-4.122861
132	6	5.579162	-4.401894	-2.333966
133	1	4.527092	-4.237279	-2.587712
134	1	5.889875	-5.342984	-2.791588
135	6	5.764413	-4.458179	-0.833969
136	6	6.644359	-5.386248	-0.260311
137	1	7.178472	-6.079936	-0.90439
138	6	6.829974	-5.439526	1.12265
139	1	7.509877	-6.172315	1.547704
140	6	6.139858	-4.558157	1.956849
141	1	6.278295	-4.603957	3.033465
142	6	5.262134	-3.624895	1.398907
143	1	4.7288	-2.925085	2.036332
144	6	5.073333	-3.581659	0.016787
145	1	4.378484	-2.862665	-0.409305
146	6	10.25845	0.200532	-0.055395
147	6	11.11447	1.112952	0.833283

148	1	12.03799	1.325537	0.281617
149	6	11.14761	3.438022	1.640664
150	6	10.29816	4.634333	1.902155
151	6	8.94803	4.603213	1.573511
152	6	8.091782	5.67173	1.798364
153	1	7.046015	5.613293	1.524085
154	6	8.634931	6.817031	2.390087
155	1	7.990313	7.669478	2.583177
156	6	9.990615	6.87685	2.732911
157	1	10.39258	7.775838	3.189472
158	6	10.82495	5.78887	2.490796
159	1	11.8801	5.800238	2.744464
160	6	11.51752	0.368769	2.142261
161	1	12.04485	-0.537272	1.828528
162	1	12.23457	1.011296	2.657115
163	6	10.36367	0.021648	3.056511
164	6	10.02616	0.859751	4.128913
165	1	10.61662	1.753894	4.311612
166	6	8.960553	0.547246	4.974634
167	1	8.722947	1.202316	5.808147
168	6	8.213019	-0.61288	4.759988
169	1	7.396212	-0.867163	5.43017
170	6	8.538061	-1.4563	3.694836
171	1	7.967144	-2.363669	3.519139
172	6	9.605931	-1.141521	2.854026
173	1	9.863908	-1.810428	2.037489
174	6	5.401972	2.928177	-0.164031
175	6	3.889605	3.201704	-0.304514
176	1	3.789606	4.211344	-0.720707
177	6	2.013504	2.504252	-1.707807
178	6	1.501044	1.425966	-2.595368
179	6	2.315548	0.321843	-2.845758
180	6	1.91406	-0.73378	-3.654253
181	1	2.572141	-1.573584	-3.835273
182	6	0.639639	-0.673972	-4.225519
183	1	0.304269	-1.489118	-4.859374
184	6	-0.1986	0.419964	-3.9915
185	1	-1.183664	0.453505	-4.44621
186	6	0.227835	1.468212	-3.180679
187	1	-0.418067	2.318865	-2.993565
188	6	3.230409	3.204066	1.10726
189	1	3.755593	3.975499	1.678912
190	1	2.193919	3.527516	0.985239
191	6	3.273981	1.884299	1.846982
192	6	4.41562	1.499345	2.565493

193	1	5.277383	2.159883	2.592264
194	6	4.451696	0.287412	3.255314
195	1	5.348644	0.009081	3.800669
196	6	3.338423	-0.556545	3.247592
197	1	3.355475	-1.488969	3.804875
198	6	2.194358	-0.185534	2.537695
199	1	1.31875	-0.82825	2.538986
200	6	2.167391	1.023885	1.840824
201	1	1.26922	1.310907	1.298988
202	11	-0.398799	4.684948	-1.091766

Sodium complex **5 (I & I)**

Sodium complex **5 (I & I)** represents the HIM/(Na)BARF complex consisting HIMs in same molecular conformation (**I** & **I**)

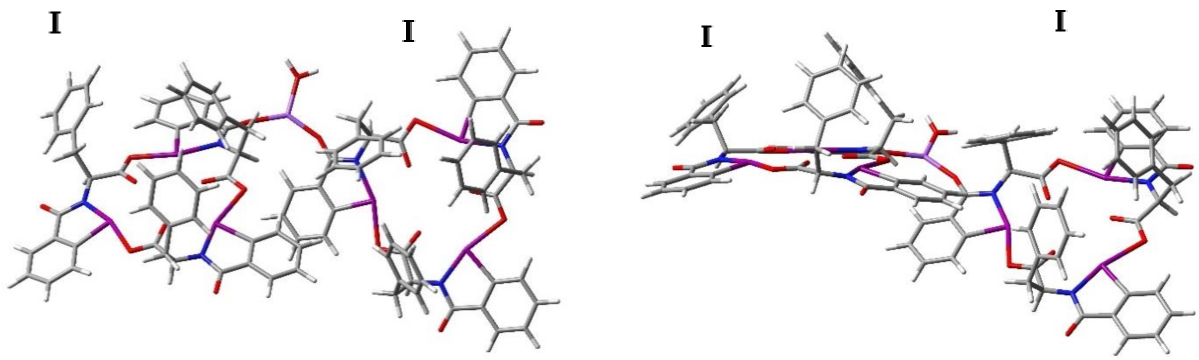

Figure S21. DFT image of sodium complex **5** (**I, I**) Left) top view. Right) side view. Both HIMs are present in same conformation, Conformers **I**. All three benzyl groups are facing towards the interior of the macrocycle in both HIMs. Sodium, Oxygen, nitrogen and the iodine atoms are denoted by lavender, red, blue and purple color respectively.

Coordinates for DFT calculated structure of sodium complex 10 (6-I, 6-I)

Job Type: geometry optimization and frequency calculation

Method: Ground state, Default spin, DFT, B3LYP

Basis Set: 6-31G(d,p) for all atoms excluding iodine, LanL2DZ for iodine

Solvent: none

Total Energy: -5690.730763 Hartree

Number of Imaginary Frequencies: 0

Center Number	Atomic Number	X	Y	Z
1	53	-4.629142	-2.295272	0.655869
2	53	-6.12672	2.505665	-1.289042
3	53	-9.729188	-1.411728	-0.662932
4	53	4.296276	0.334219	-1.964807
5	53	8.982282	-2.242106	-1.432565
6	53	8.433207	2.672205	0.566985
7	8	-3.019034	-0.316464	0.356531
8	8	-4.994881	0.460052	-0.329494
9	8	-2.303825	4.356802	-1.570805
10	8	-8.424874	2.779705	-1.84928
11	8	-8.286129	0.716879	-1.039278
12	8	-12.95904	0.571069	-2.478201
13	8	-8.925053	-3.539673	0.279481
14	8	-7.116094	-2.24347	0.234741

15	8	-4.898653	-6.36511	1.624365
16	8	4.667051	-1.866591	-2.822707
17	8	6.596194	-1.308379	-1.873744
18	8	7.952319	-5.665473	-3.644603
19	8	10.77796	-1.070987	-0.231433
20	8	9.079114	0.365699	-0.216145
21	8	12.34897	3.290714	1.926053
22	8	6.129451	3.745908	0.276686
23	8	5.936541	1.778033	-0.755828
24	8	1.457798	3.477735	-1.778869
25	8	0.355821	6.767389	-1.247548
26	1	-0.165467	7.508578	-0.912525
27	1	1.2748	7.058095	-1.177724
28	7	-3.993702	2.898098	-1.020287
29	7	-10.91815	0.147785	-1.53163
30	7	-5.4544	-4.206738	1.111343
31	7	7.812671	-3.675626	-2.520931
32	7	10.46036	2.294266	1.106528
33	7	3.327699	2.188627	-1.436923
34	6	-3.774344	0.601324	-0.08283
35	6	-3.126705	1.988736	-0.271991
36	1	-2.219019	1.828786	-0.86541
37	6	-3.522634	4.018382	-1.581125
38	6	-4.566	4.856817	-2.227058
39	6	-5.893739	4.429617	-2.202795
40	6	-6.924531	5.166919	-2.770195
41	1	-7.944435	4.8057	-2.742769
42	6	-6.599741	6.383122	-3.379172
43	1	-7.389992	6.978491	-3.826196
44	6	-5.27643	6.833728	-3.421167
45	1	-5.041712	7.77735	-3.903436
46	6	-4.260502	6.074215	-2.848233
47	1	-3.227257	6.403661	-2.871937
48	6	-2.70228	2.563343	1.113481
49	1	-2.011197	1.833687	1.546898
50	1	-2.139994	3.484031	0.932691
51	6	-3.839265	2.839778	2.073035
52	6	-4.37019	4.130444	2.202851
53	1	-3.950511	4.943859	1.61486
54	6	-5.416545	4.388565	3.089982
55	1	-5.808471	5.397133	3.185594
56	6	-5.949164	3.353825	3.861383
57	1	-6.75325	3.556205	4.56313
58	6	-5.431131	2.062327	3.73968
59	1	-5.83919	1.248401	4.331514

60	6	-4.381863	1.810349	2.856075
61	1	-3.972856	0.806996	2.777805
62	6	-8.952223	1.65462	-1.517799
63	6	-10.4671	1.527476	-1.703781
64	1	-10.69241	1.820522	-2.736285
65	6	-12.1802	-0.208434	-1.932279
66	6	-12.51624	-1.627142	-1.617525
67	6	-11.57888	-2.438742	-0.988129
68	6	-11.82179	-3.763554	-0.655596
69	1	-11.0638	-4.367411	-0.172456
70	6	-13.08197	-4.282208	-0.972407
71	1	-13.30591	-5.314856	-0.722305
72	6	-14.04811	-3.490791	-1.60377
73	1	-15.01915	-3.913188	-1.842112
74	6	-13.7696	-2.165571	-1.927802
75	1	-14.49545	-1.524192	-2.417296
76	6	-11.21283	2.532539	-0.771082
77	1	-12.264	2.496778	-1.064355
78	1	-10.83135	3.52871	-1.016957
79	6	-11.06388	2.253911	0.707509
80	6	-12.03701	1.514401	1.39474
81	1	-12.916	1.164735	0.859553
82	6	-11.89932	1.240837	2.756507
83	1	-12.66863	0.674479	3.273653
84	6	-10.78213	1.704095	3.454622
85	1	-10.68095	1.502753	4.517486
86	6	-9.807066	2.444584	2.782809
87	1	-8.93504	2.813343	3.314944
88	6	-9.950412	2.718697	1.422462
89	1	-9.195425	3.309804	0.910384
90	6	-7.687584	-3.328244	0.493461
91	6	-6.892465	-4.468698	1.142584
92	1	-7.071008	-5.369943	0.544536
93	6	-4.557792	-5.210967	1.376136
94	6	-3.139089	-4.754998	1.343889
95	6	-2.852184	-3.424647	1.061842
96	6	-1.556355	-2.930606	0.999765
97	1	-1.368087	-1.891421	0.759648
98	6	-0.512357	-3.827872	1.249163
99	1	0.513818	-3.475807	1.208381
100	6	-0.772015	-5.171388	1.5439
101	1	0.054937	-5.849225	1.730491
102	6	-2.082596	-5.638605	1.59122
103	1	-2.321117	-6.674217	1.811509
104	6	-7.430039	-4.751594	2.578153

105	1	-6.911826	-5.644847	2.933263
106	1	-8.488827	-5.003804	2.463118
107	6	-7.260921	-3.612073	3.558432
108	6	-6.164484	-3.577518	4.4318
109	1	-5.444102	-4.39142	4.414246
110	6	-6.001692	-2.526271	5.335718
111	1	-5.152748	-2.523865	6.013705
112	6	-6.936138	-1.489204	5.379746
113	1	-6.820074	-0.680449	6.096204
114	6	-8.033095	-1.511043	4.515002
115	1	-8.76886	-0.712226	4.541209
116	6	-8.193638	-2.56568	3.61596
117	1	-9.057884	-2.584902	2.957579
118	6	5.874431	-2.130635	-2.469799
119	6	6.380012	-3.54414	-2.776273
120	1	6.217792	-3.720264	-3.846694
121	6	8.48586	-4.777717	-2.981117
122	6	9.921292	-4.79898	-2.576577
123	6	10.44219	-3.760794	-1.812115
124	6	11.76329	-3.715909	-1.390921
125	1	12.13516	-2.886509	-0.802356
126	6	12.58943	-4.783021	-1.759892
127	1	13.62822	-4.780214	-1.443755
128	6	12.0952	-5.84402	-2.526945
129	1	12.75355	-6.661261	-2.804134
130	6	10.7645	-5.855603	-2.93667
131	1	10.34893	-6.662227	-3.532052
132	6	5.52317	-4.598541	-2.008079
133	1	4.482711	-4.426868	-2.302025
134	1	5.82223	-5.575104	-2.39338
135	6	5.666757	-4.558829	-0.502938
136	6	6.500757	-5.472724	0.155812
137	1	7.029101	-6.223948	-0.425451
138	6	6.648112	-5.438723	1.543974
139	1	7.292476	-6.161775	2.03581
140	6	5.965517	-4.482355	2.29782
141	1	6.074413	-4.459547	3.378559
142	6	5.133555	-3.562222	1.654458
143	1	4.607429	-2.804611	2.228679
144	6	4.982303	-3.60633	0.267793
145	1	4.322016	-2.896784	-0.224086
146	6	10.24785	0.040397	0.095875
147	6	11.10125	0.989588	0.948088
148	1	12.04124	1.149886	0.406386
149	6	11.16195	3.358108	1.615725

150	6	10.33272	4.585855	1.776888
151	6	8.993347	4.563727	1.406258
152	6	8.155702	5.662125	1.538121
153	1	7.118085	5.609818	1.233314
154	6	8.706564	6.829369	2.077542
155	1	8.076317	7.705636	2.197657
156	6	10.05177	6.880865	2.46061
157	1	10.46006	7.797135	2.875275
158	6	10.86762	5.762529	2.311912
159	1	11.91425	5.766678	2.598909
160	6	11.46013	0.320075	2.309158
161	1	11.97269	-0.614922	2.063375
162	1	12.18096	0.977789	2.798799
163	6	10.28053	0.05568	3.218165
164	6	9.941361	0.966121	4.229278
165	1	10.54862	1.856812	4.368576
166	6	8.852696	0.729832	5.070384
167	1	8.614034	1.440401	5.856807
168	6	8.083177	-0.425011	4.91226
169	1	7.247779	-0.619419	5.579559
170	6	8.409669	-1.339975	3.908353
171	1	7.821668	-2.243976	3.77729
172	6	9.500771	-1.101425	3.072358
173	1	9.759726	-1.825475	2.304638
174	6	5.465071	2.864347	-0.343998
175	6	3.963494	3.158664	-0.54573
176	1	3.896158	4.142026	-1.026257
177	6	2.11929	2.418529	-1.96934
178	6	1.608406	1.29649	-2.801647
179	6	2.398559	0.156264	-2.945399
180	6	1.994642	-0.941946	-3.693727
181	1	2.633599	-1.80982	-3.791874
182	6	0.7435	-0.887727	-4.314884
183	1	0.406548	-1.73593	-4.902838
184	6	-0.069445	0.242532	-4.188896
185	1	-1.036051	0.27115	-4.681912
186	6	0.359068	1.332692	-3.436543
187	1	-0.266165	2.212721	-3.333849
188	6	3.26451	3.260398	0.842908
189	1	3.774655	4.069761	1.374281
190	1	2.232654	3.575233	0.668749
191	6	3.285275	1.996305	1.675302
192	6	4.398097	1.676365	2.467269
193	1	5.252584	2.346657	2.480901
194	6	4.414819	0.517354	3.243279

195	1	5.289937	0.287587	3.843913
196	6	3.309681	-0.337098	3.249374
197	1	3.310238	-1.226831	3.872774
198	6	2.194374	-0.030846	2.466626
199	1	1.325224	-0.68217	2.477792
200	6	2.18715	1.125177	1.68382
201	1	1.311281	1.361742	1.08426
202	11	-0.237825	4.71481	-1.586263

References:

1. Pandey K, Arafin S, Jones E, Du Y, Kulkarni GC, Uddin A, et al. *J. Org. Chem.* **2024,** *89*, 7437-7445.
